# Supplementary material for: Patterns of healthcare utilisation in children and young people: a retrospective cohort study using routinely collected healthcare data in Northwest London
Source: BMJ Open. 2021 Dec 17;11(12):e050847. doi: 10.1136/bmjopen-2021-050847 (PMC8685945; doi:10.1136/bmjopen-2021-050847)
Supplement: Supplementary data [file bmjopen-2021-050847supp001.pdf]

**Patterns of healthcare utilisation in children and young people: a retrospective cohort study using routinely collected healthcare data in Northwest London**

Thomas Beaney[1,2]

Jonathan M Clarke[3]

Thomas Woodcock[1,2]

Rachel McCarthy[4]

Kavitha Saravanakumar[4]

Mauricio Barahona[3]

Mitch Blair[1,2]

Dougal Hargreaves[1,2]

1. Department of Primary Care and Public Health, Imperial College London, London, W6 8RP, UK.
2. National Institute for Health Research Applied Research Collaboration Northwest London, Imperial College, London, W6 8RP, UK.
3. Department of Mathematics and Centre for Mathematics of Precision Healthcare, Imperial College London, London, SW7 2AZ, UK.
4. North West London Collaboration of Clinical Commissioning Groups, London, NW1 5JD, UK

**Corresponding author:**

Thomas Beaney

Department of Primary Care and Public Health, Imperial College London, London, W6 8RP, UK.

Email: Thomas.beaney@imperial.ac.uk

## Appendix

## Patterns of healthcare utilisation in children and young people: a retrospective cohort study using routinely collected healthcare data in Northwest London

Note: some of the text in the appendix is repeated from the manuscript to provide further detail.

### Healthcare utilisation

Seven healthcare utilisation measures were selected for inclusion:

1. GP attendances
2. A&E attendances
3. Outpatient attendances
4. Elective hospital admissions
5. Total length of stay from elective admissions in days
6. Emergency hospital admissions
7. Total length of stay from emergency admissions in days

Numbers of attendances or admissions were summed across the year. GP attendances included all consultation types collectively (eg. face to face, telephone, home visits) and staff types (eg. GP, nurse) due to variable recording of these factors in the Discover dataset, with exclusion of events linked only to administrative Read codes, and includes visits for preventive reasons. Where more than one event of the same type occurred on a single date, only one attendance was counted. Total length of stay was calculated as the total number of inpatient days across the year, separately for elective and emergency admissions. Where a patient was admitted and discharged on the same day, the length of stay for that admission is recorded as zero days.



Beaney T, *et al. BMJ Open* 2021; 11:e050847. doi: 10.1136/bmjopen-2021-050847

**Long-term conditions**

Discover includes a long-term condition (LTC) table, listing 41 chronic conditions based on Read V2 codes. A sub-set of 17 of these were selected for inclusion based on Aitchison *et al* (2020), who identified relevant LTCs from Discover applicable to children (Table A1).[1] Certain important diagnostic categories, such as congenital heart disease are not included in the Discover LTC list and so additional diagnosis codes were sourced. Raw Read codes are translated into SNOMED in the GP events table on Discover. Additional SNOMED code clusters were extracted for asthma, obesity, congenital heart disease, chronic kidney disease, diabetes, immunosuppression, learning disability, liver disease, neurological disease and chronic respiratory disease based on available code clusters listed by NHS as risk factors for influenza vaccination.[2] Chronic heart disease codes were combined with coronary heart disease and heart failure, and chronic neurological disease was combined with cerebral palsy, epilepsy and spina bifida (Table A2). Number of LTCs were counted as the number of categories of LTC from Table A3. Relevant codes were identified from the entirety of the patient primary care record and secondary care diagnostic codes were not included in order not to bias number of conditions in favour of those presenting to secondary care.



Beaney T, *et al. BMJ Open* 2021; 11:e050847. doi: 10.1136/bmjopen-2021-050847

**Table S1: 41 Long-term conditions identified in Discover (included conditions highlighted)**

| | |
|---|---|
| **Asthma** * | **Hypothyroidism** * |
| Atrial Fibrillation | **Anxiety** * |
| **Cancer** * | Charcot Marie Tooth Disease |
| **CKD** * | Dystonia Primary Idiopathic |
| COPD | Essential Tremor |
| Dementia | Huntingtons Disease |
| **Depression** * | Motor Neurone Disease |
| **Diabetes** * | Multiple Sclerosis |
| **Epilepsy** * | Multiple System Atrophy |
| **Heart Failure** * | Muscular Dystrophy |
| Hypertension | Myalgic Encephalopmyelitis |
| **Learning Disability** * | Myasthenia Gravis |
| **Mental Health** * | Parkinsons Disease |
| **Obesity** * | Progressive Supranuclear Palsy |
| Osteoporosis | Transverse Myelitis |
| Peripheral Arterial Disease | **Cerebal Palsy** * |
| **Palliative care** * | **Spina Bifida and Hydrocephalus** * |
| **Rheumatoid Arthritis** * | Ataxia |
| **Coronary Heart Disease** * | Ataxia-Telangiectasia |
| Stroke/TIA | Encephalitis |
| Ischaemic Heart Disease | |

* Included in analysis

**Table S2: Defined long-term conditions and source of codes from Discover and SNOMED**

| **Long-term condition (LTC)** | **Discover LTC table** | **Additional SNOMED clusters**[2] |
|---|---|---|
| Asthma | Asthma | Asthma |
| Obesity | Obesity | Obesity |
| Mental health condition | Anxiety; depression; mental health | - |
| Learning Disability | Learning disability | Learning disability |
| Chronic heart disease | Coronary heart disease; heart failure | Chronic heart disease |
| Chronic neurological disease & epilepsy | Cerebral palsy; epilepsy; spina bifida | Chronic neurological disease |
| Hypothyroidism | Hypothyroidism | - |
| Cancer & immunosuppression | Cancer | Immunosuppression |
| Diabetes | Diabetes | Diabetes |
| Chronic respiratory disease (non-asthma) | - | Chronic respiratory disease |
| Chronic kidney disease | Chronic kidney disease | Chronic kidney disease |
| Palliative care | Palliative care | - |
| Rheumatoid arthritis | Rheumatoid arthritis | - |



Beaney T, *et al. BMJ Open* 2021; 11:e050847. doi: 10.1136/bmjopen-2021-050847

**Selection of number of clusters**

Calinski-Harabasz and Davies-Bouldin scores were calculated for *k* in the range 2-14.[3, 4] The former calculates the ratio of between-cluster sum of squared distances to the within-cluster sum of squared distances, with larger values indicating better dispersion. The latter calculates the average similarity of clusters, based on the average distance of each point in the cluster to the centroid, and the distance between cluster centroids, with smaller values indicating better separation. Table S3 displays both scores by value of *k*.

**Table S3: Calinski-Harabasz and Davies-Bouldin scores for values of *k* clusters**

| k | Calinski-Harabasz | Davies-Bouldin |
|---|---|---|
| 2 | 237582 | 1.09368 |
| 3 | 221031 | 1.15472 |
| 4 | 228535 | 0.99956 |
| 5 | 212661 | 1.07835 |
| 6 | 211027 | 0.99648 |
| 7 | 205075 | 1.00265 |
| 8 | 199811 | 1.03196 |
| 9 | 193744 | 1.02996 |
| 10 | 187409 | 1.07746 |
| 11 | 184038 | 1.04232 |
| 12 | 180593 | 1.01880 |
| 13 | 176750 | 1.02698 |
| 14 | 173582 | 1.02538 |

The Calinski-Harabasz score was highest for two clusters and declined gradually from four clusters, indicating better dispersion with a smaller number of clusters. The Davies-Bouldin score was lowest for six clusters, with similarly low scores for four, seven and eight clusters. The range from four to eight were selected as a focus for further description. Figures S1-S5 show the distributions of mean activity across the seven healthcare utilisation variables for each of the five models. Confusion matrices (Figures S6-S9) show the changes in assignment between consecutive increases in *k* clusters.



Beaney T, *et al. BMJ Open* 2021; 11:e050847. doi: 10.1136/bmjopen-2021-050847

Utilisation within each cluster was assigned a descriptor based on total utilisation (low if mean attendances below average for all attendance types; medium if above average for at least one and fewer than four attendance types; and high if above average for four or more attendance types) and predominant attendance types accounting for utilisation.

In a 4-cluster model (Figures S1a and S1b), a low utilisation group and a moderate, GP predominant segment were formed. Two high utilisation segments were also formed, one utilising predominantly GP and emergency care, and the other using all services. The 5-cluster model (Figures S2a and S2b) retained the first two clusters, but split the remaining higher utilisation clusters into a moderate; GP, outpatient and elective care segment, and a moderate utilisation; GP and emergency care group, retaining in addition a smaller high utilisation cluster. The 6-cluster (Figures S3a and S3b) model split the very low and the moderate; GP clusters into a new low utilisation; GP cluster.

The 7-cluster model (Figures S4a and S4b) split three segments (very low; low utilisation, GP; and moderate utilisation, GP and emergency care) into an additional low utilisation group characterised by lower than average GP use, but higher emergency care use. The 8-cluster model (Figures S5a and S5b) split the moderate utilisation GP, outpatient and elective care group into an additional moderate utilisation cluster characterised by lower than average GP use, but higher elective care use. Participants in other segments from the 7-cluster model were also reassigned on increasing to 8-clusters, suggesting inherently less stable clusters. The additional clusters produced by the 7- and 8-cluster models were decided to be less useful clinically as descriptors given the added complexity of the model, and the 6-cluster model was selected as the optimal number of segments.

**Hierarchical clustering**

Hierarchical agglomerative clustering using Ward's method was computationally feasible on sub-samples of up to 50,000 participants (13.2% of the total sample). In order to compare the similarity of cluster assignment between different random draws, a fixed sub-sample of 5,000 participants was randomly selected ($R_F$). The clustering algorithm was then iterated 10 times. On each iteration, a new

sub-sample of 45,000 participants ($R_V$) was randomly selected, added to $R_F$ to create a total sub-sample of 50,000 participants ($R_F + R_V$). Pair-wise comparisons of cluster membership for participants in $R_F$ from each iteration compared to the preceding iteration were made using V-measure scores, the harmonic mean of homogeneity and completeness scores.[5] A range of V-measure scores from 0.765 – 0.918 were calculated, with a mean of 0.847, suggesting that cluster assignment was sensitive the sub-sample of data used.



Beaney T, *et al. BMJ Open* 2021; 11:e050847. doi: 10.1136/bmjopen-2021-050847

**Figure S1a: Mean number of attendances to outpatients, GP and A&E from the 4 cluster model**

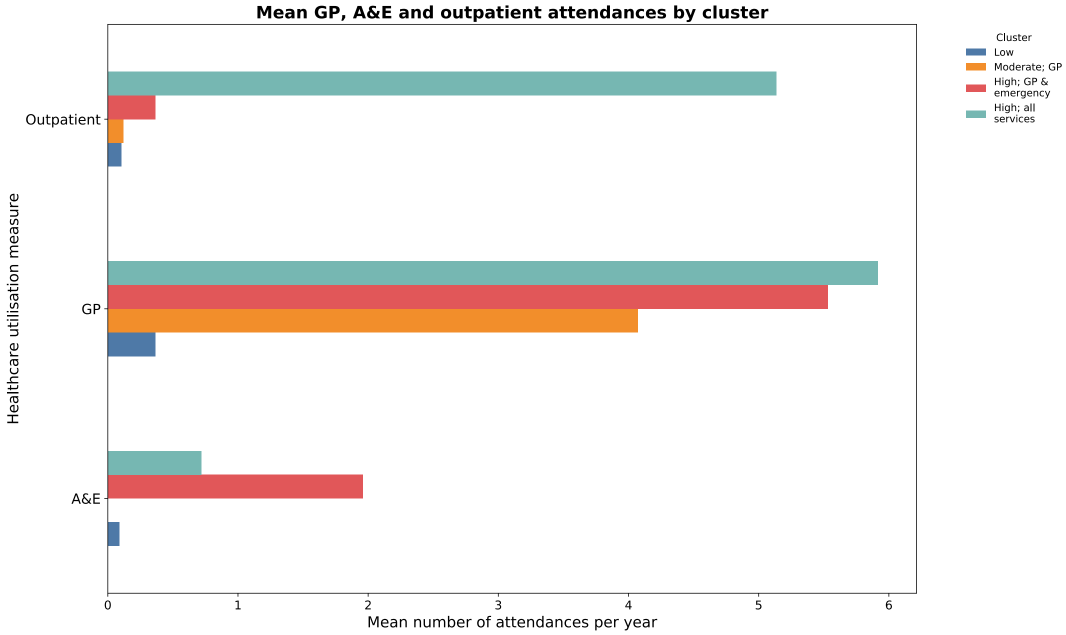


**Figure S1b: Mean number of elective and emergency admission and yearly length of stay from the 4 cluster model**

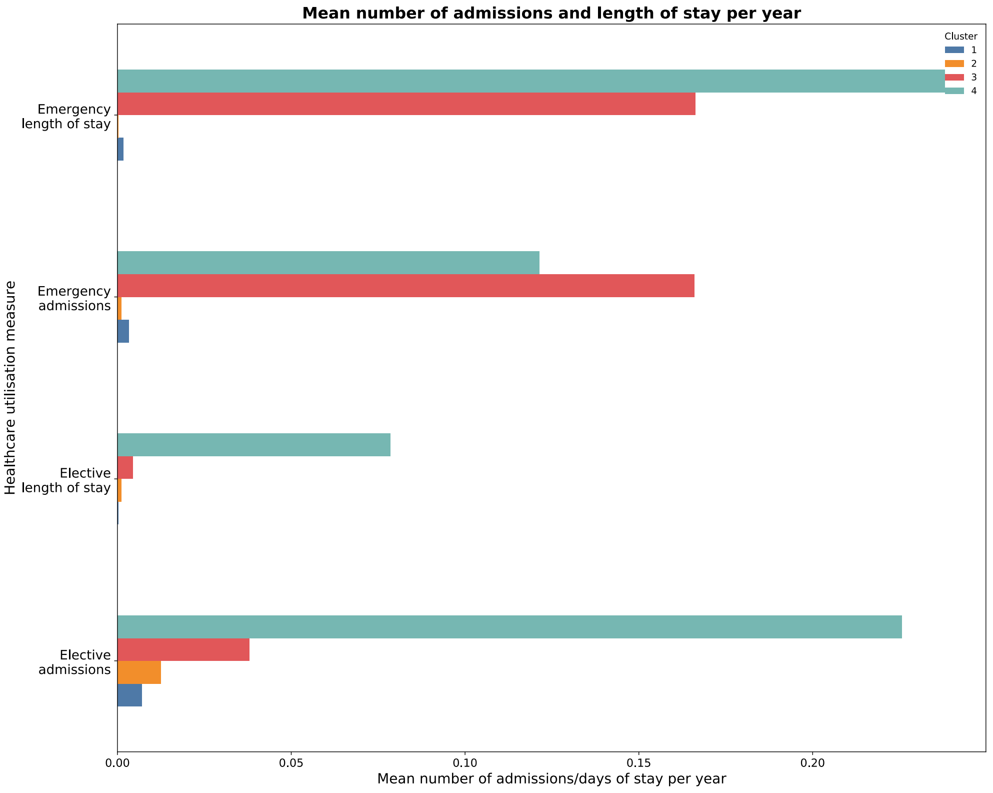

**Figure S2a: Mean number of attendances to outpatients, GP and A&E from the 5 cluster model**

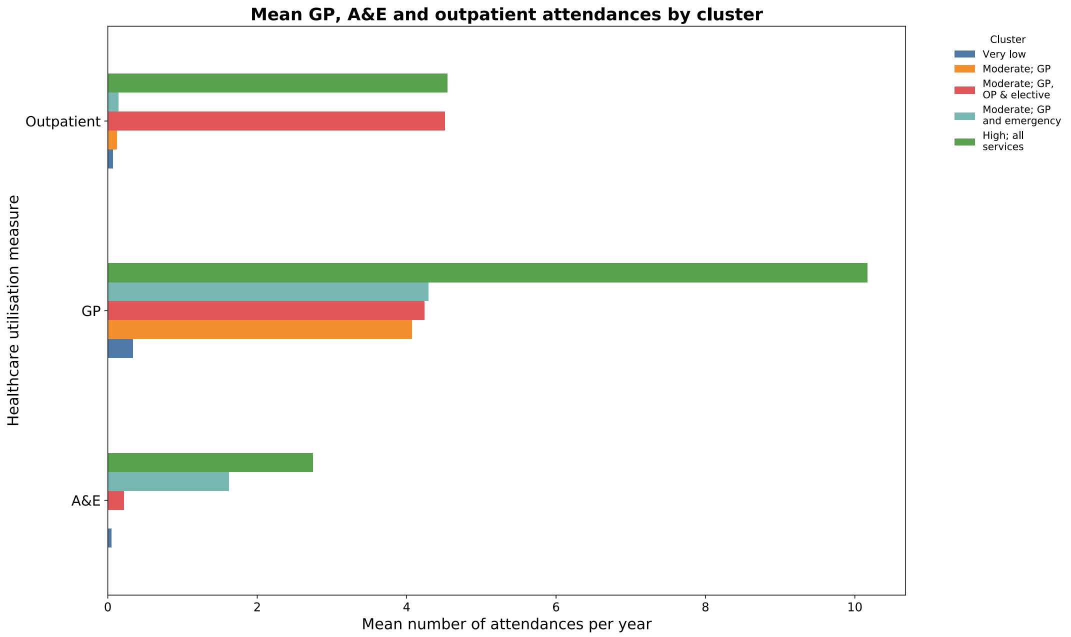


**Figure S2b: Mean number of elective and emergency admission and yearly length of stay from the 5 cluster model**

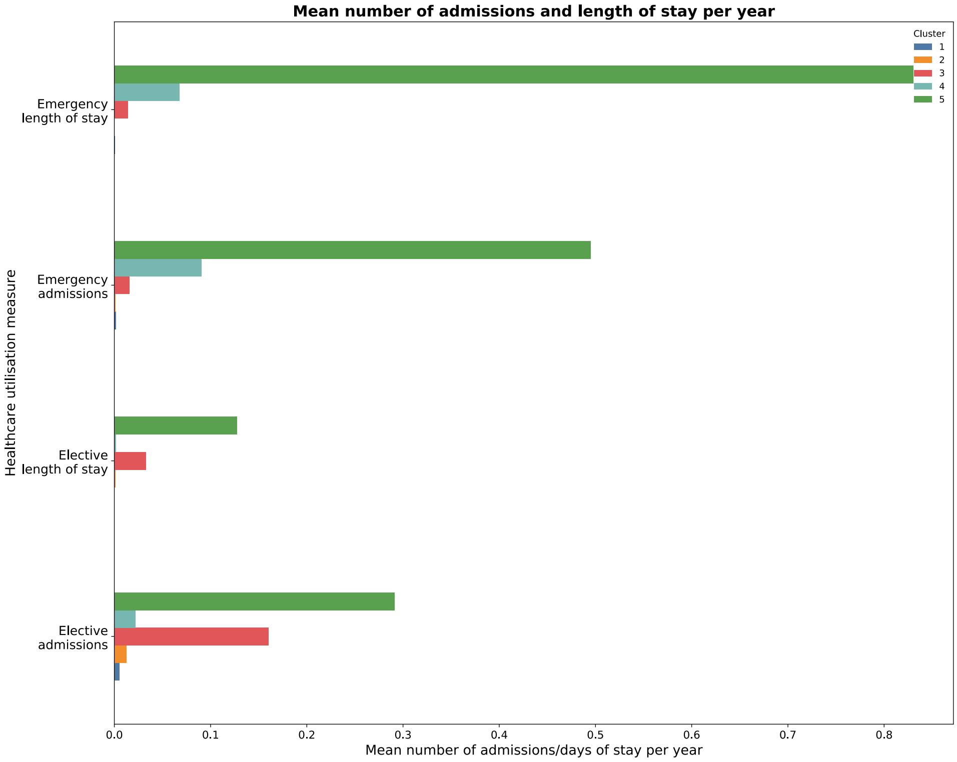


9

**Figure S3a: Mean number of attendances to outpatients, GP and A&E from the 6 cluster model**

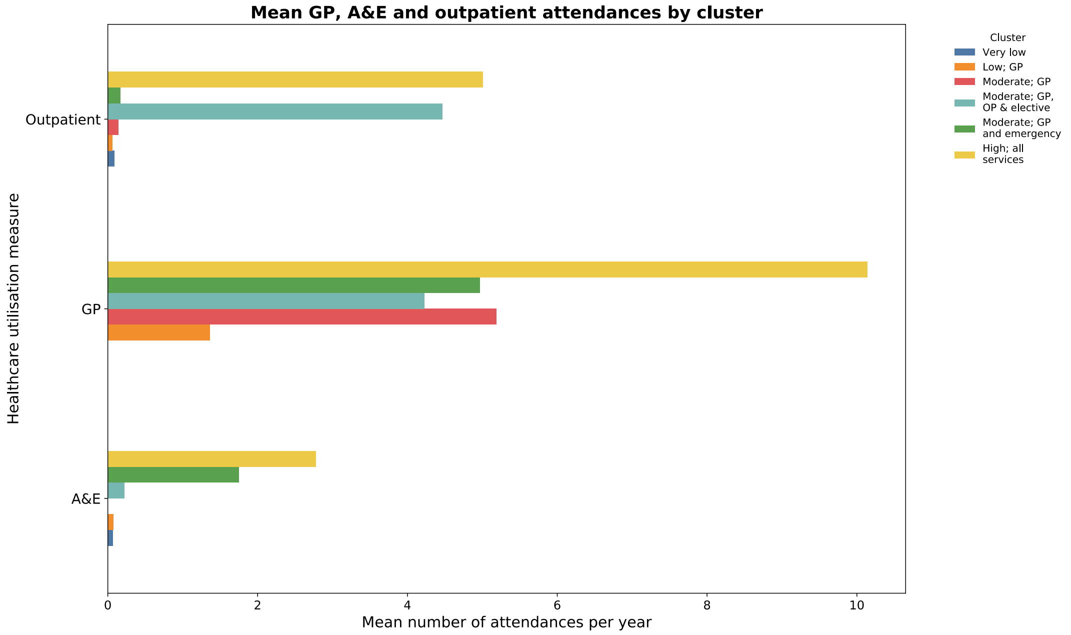


**Figure S3b: Mean number of elective and emergency admission and yearly length of stay from the 6 cluster model**

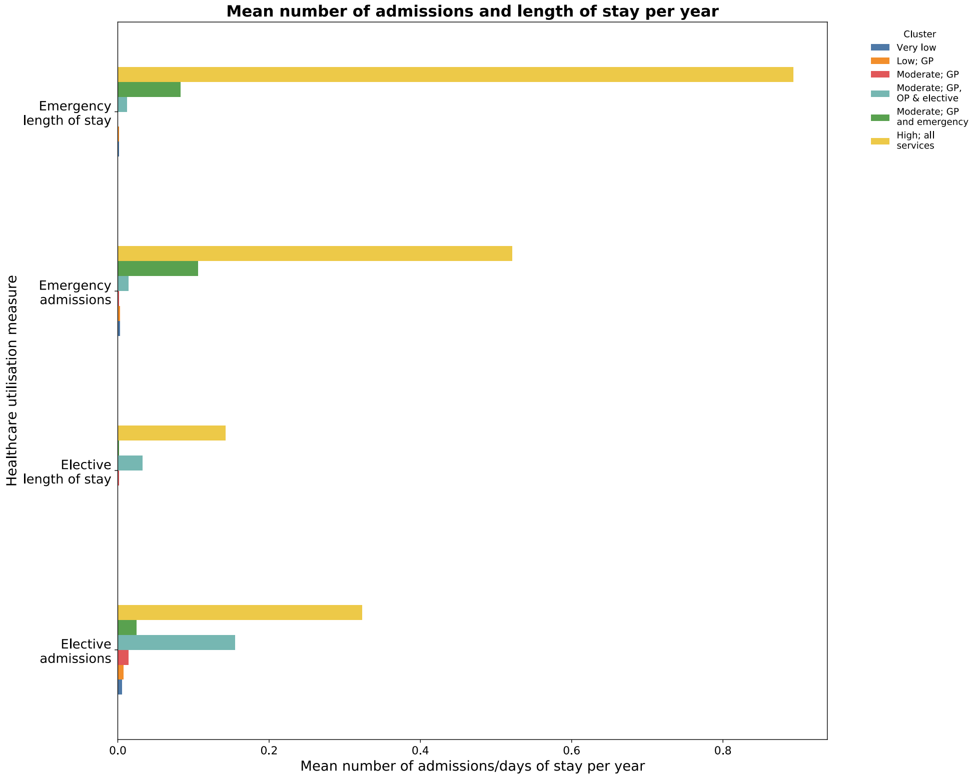

**Figure S4a: Mean number of attendances to outpatients, GP and A&E from the 7 cluster model**

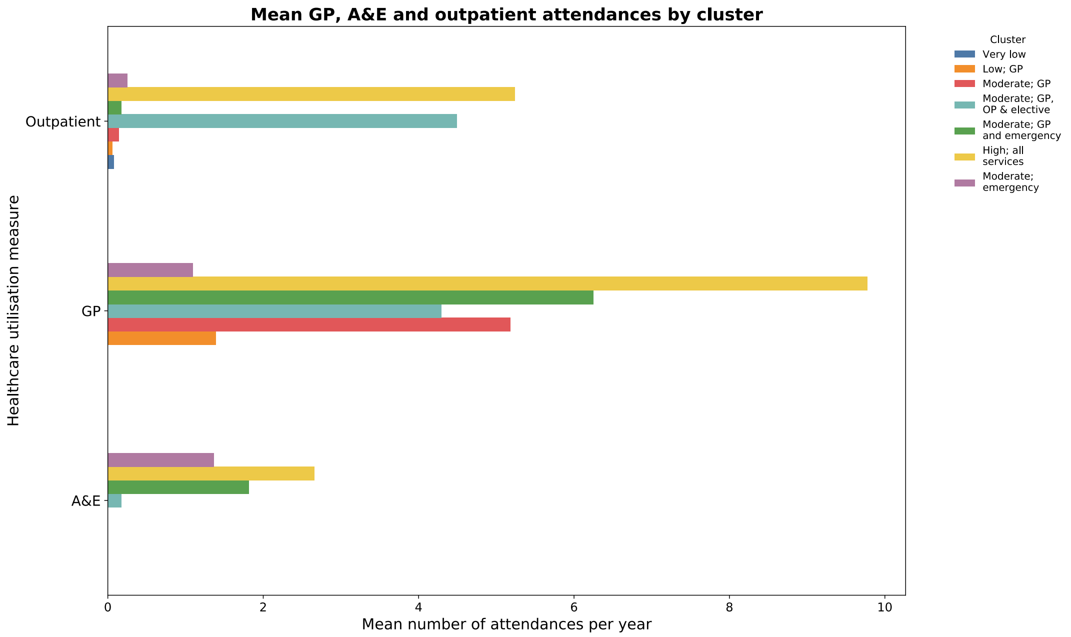


**Figure S4b: Mean number of elective and emergency admission and yearly length of stay from the 7 cluster model**

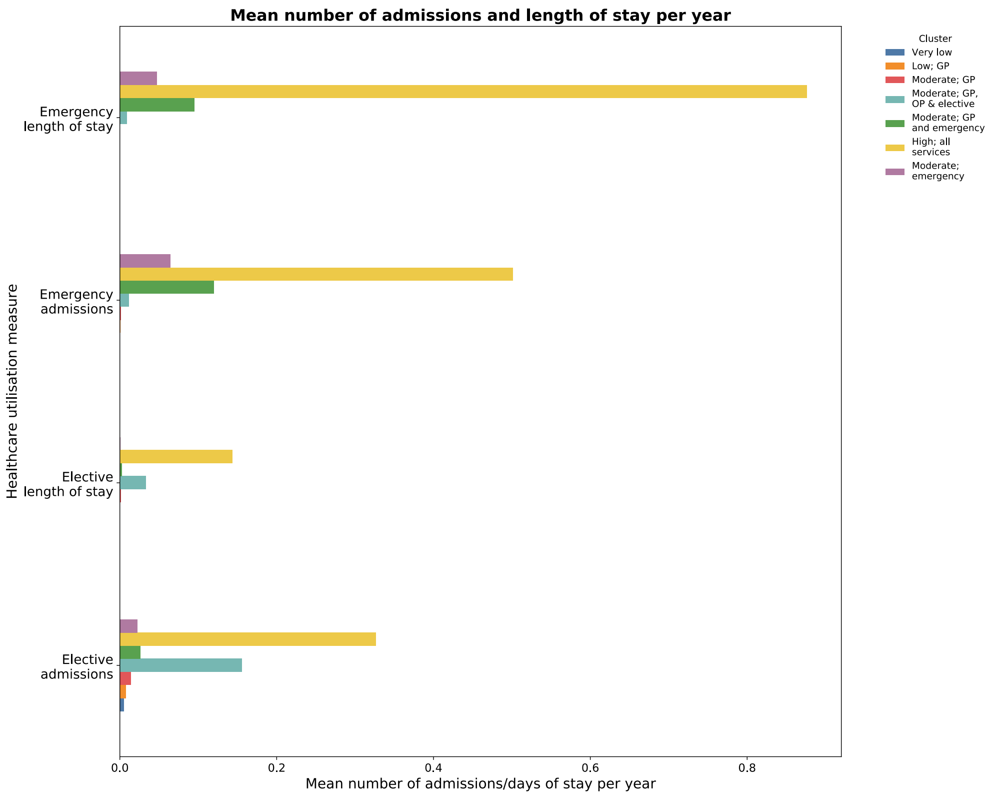

**Figure S5a: Mean number of attendances to outpatients, GP and A&E from the 8 cluster model**

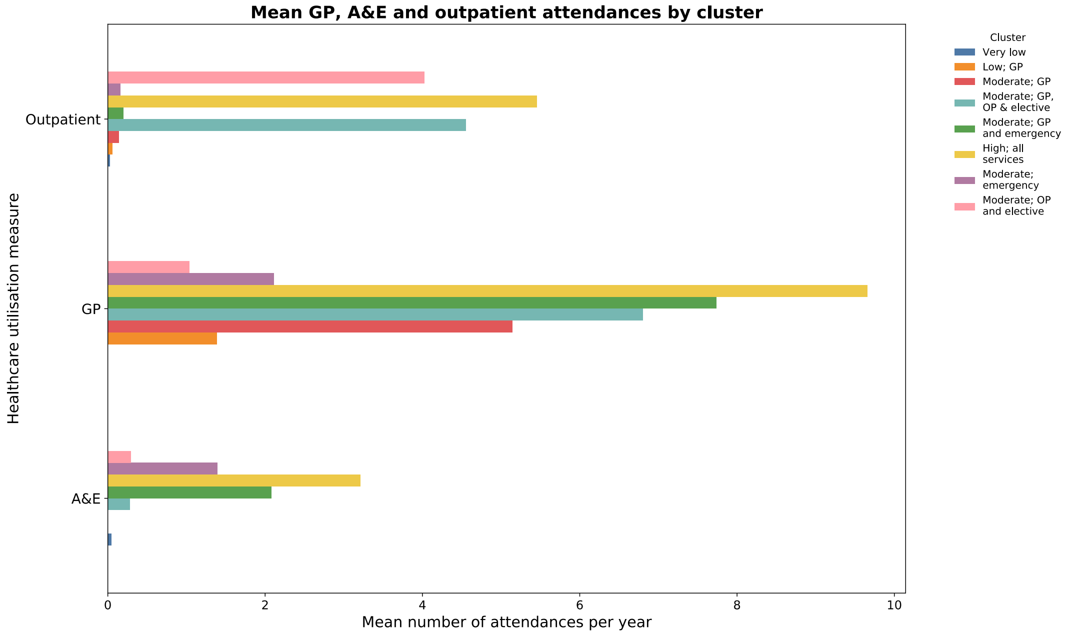


**Figure S5b: Mean number of elective and emergency admission and yearly length of stay from the 8 cluster model**

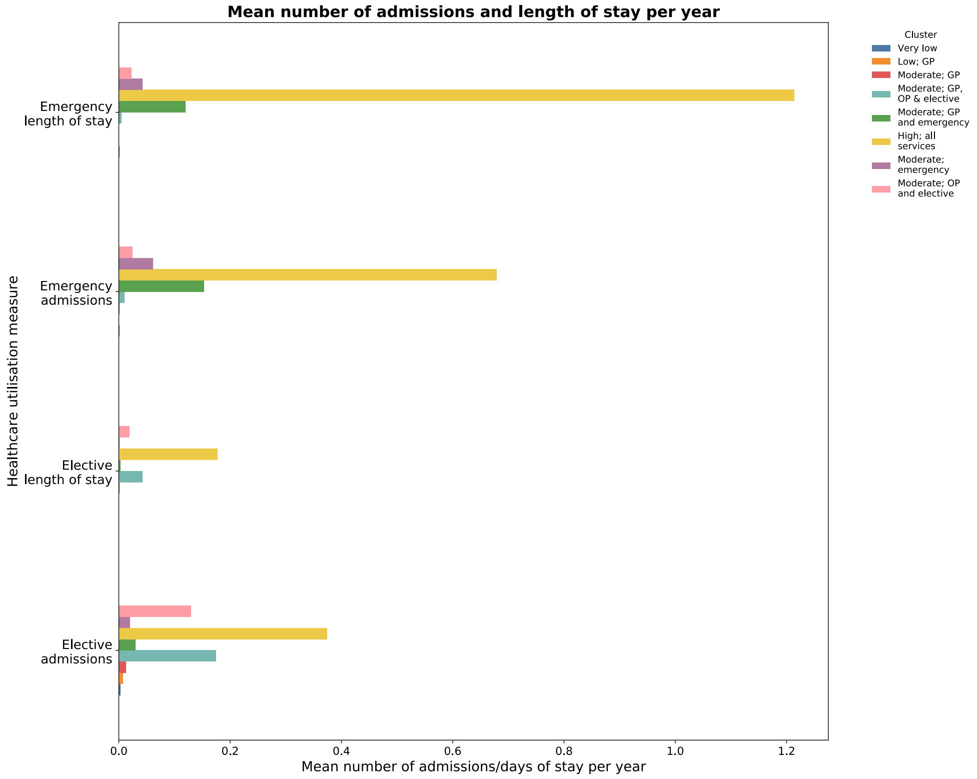

**Figure S6: Confusion matrix for cluster assignment comparing 4 vs 5 clusters from k means**

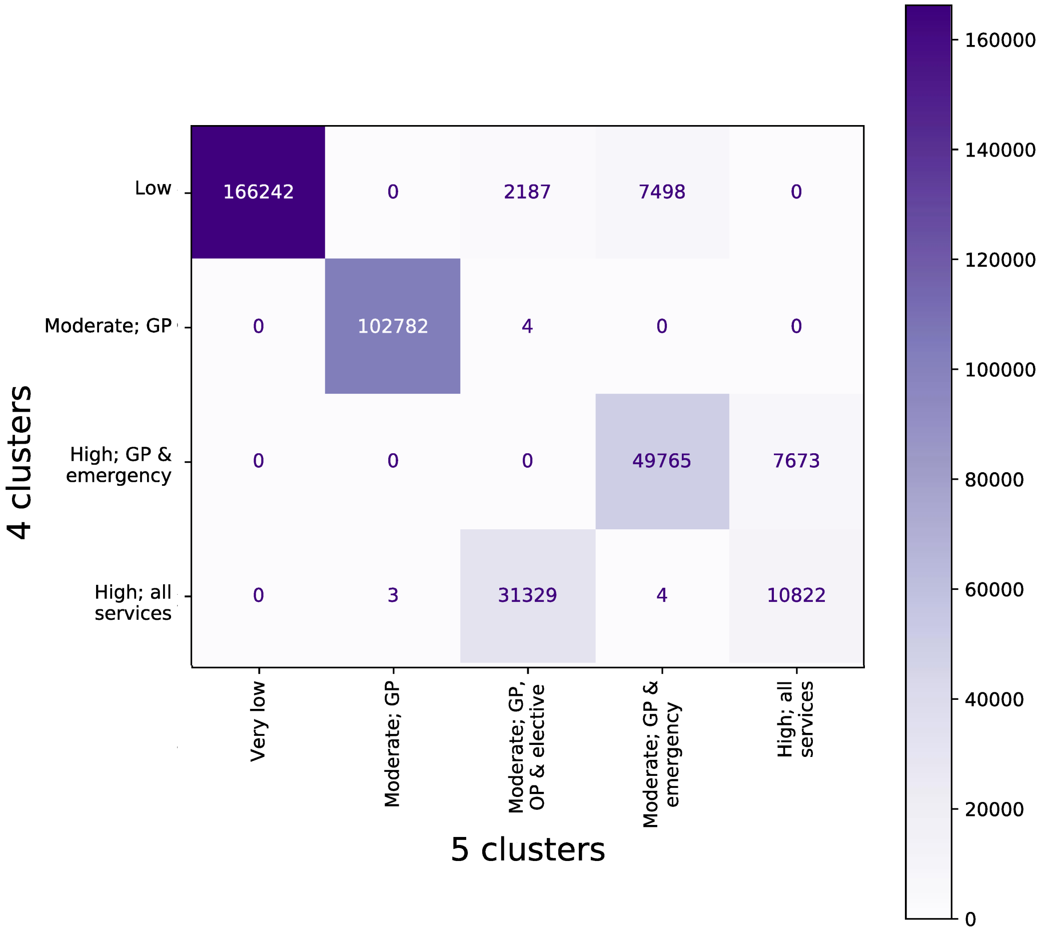

**Figure S7: Confusion matrix for cluster assignment comparing 5 vs 6 clusters from k means**

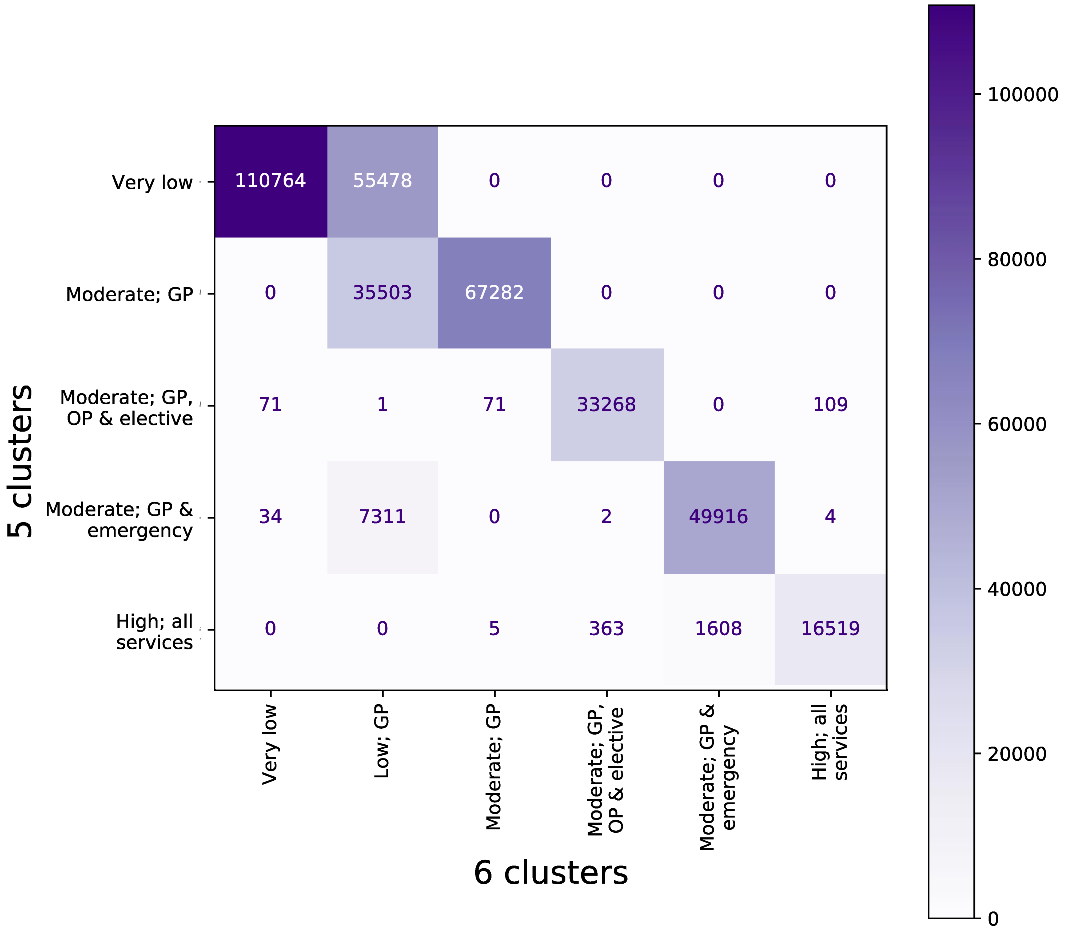

**Figure S8: Confusion matrix for cluster assignment comparing 6 vs 7 clusters from k means**

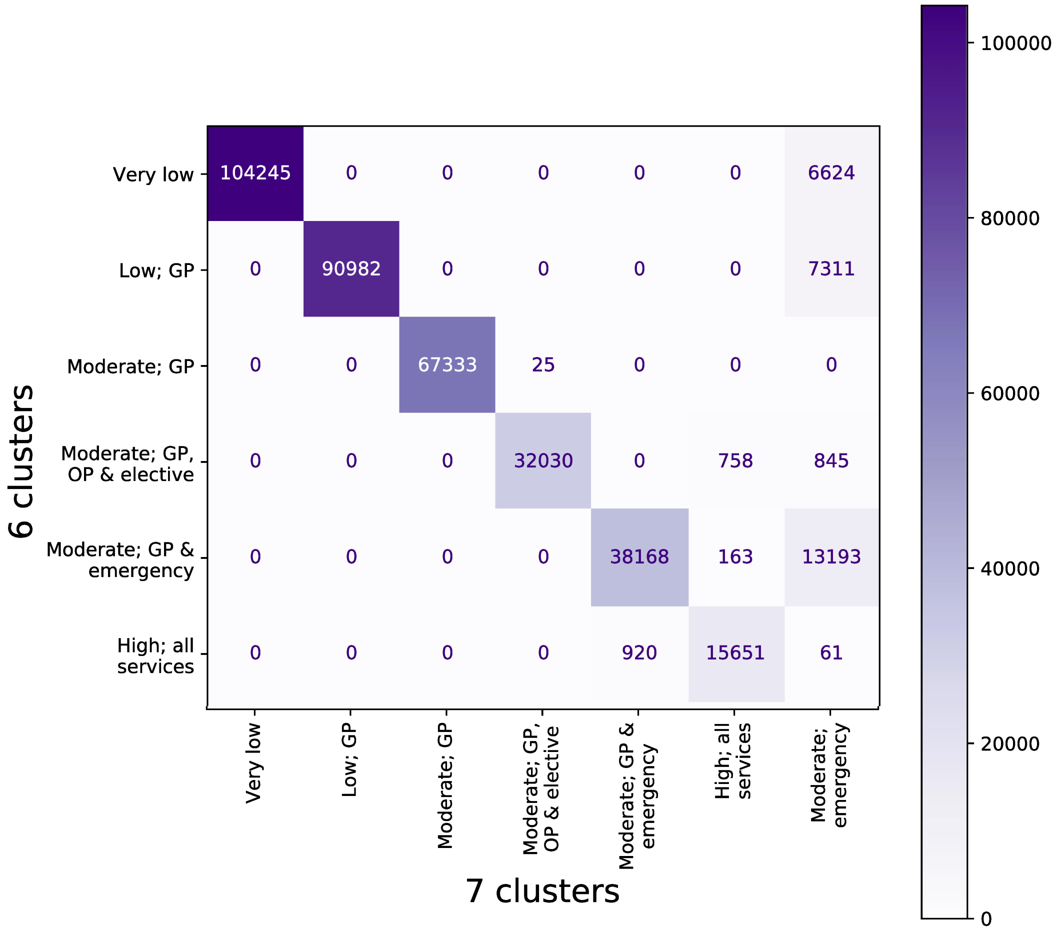




Beaney T, *et al. BMJ Open* 2021; 11:e050847. doi: 10.1136/bmjopen-2021-050847

**Figure S9: Confusion matrix for cluster assignment comparing 7 vs 8 clusters from k means**

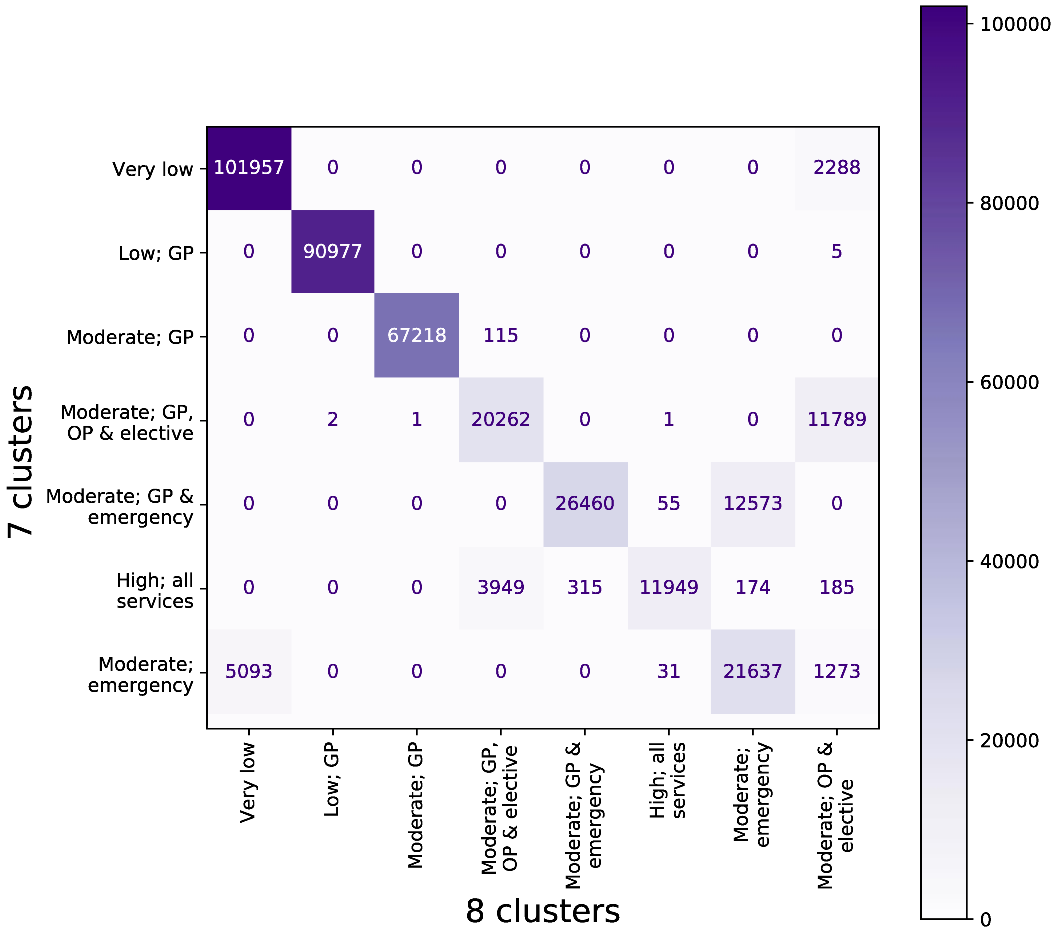

**Figure S10: Confusion matrix for cluster assignment 6 clusters comparing models from k means and equivalent hierarchical clustering using Ward's method**

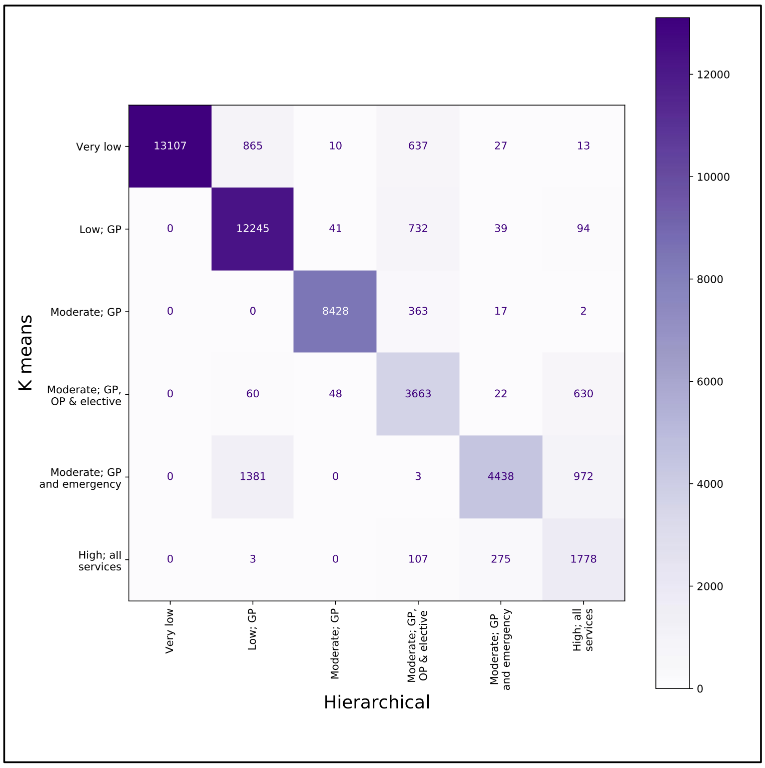




Beaney T, *et al. BMJ Open* 2021; 11:e050847. doi: 10.1136/bmjopen-2021-050847

**Table S4: Mean utilisation across each segment by utilisation variable from 6 cluster hierarchical model on subsample of 50,000 participants (green indicates lower than average utilisation, red indicates higher than average utilisation)**

| Segment | Size | Percentage | GP | Outpatient | A&E | Elective admissions | Elective length of stay | Emergency admissions | Emergency length of stay |
|---|---|---|---|---|---|---|---|---|---|
| I: No health service utilisation | 12893 | 25.8% | 0.00 | 0.00 | 0.00 | 0.00 | 0.00 | 0.00 | 0.00 |
| II: Low; GP | 13860 | 27.7% | 1.70 | 0.00 | 0.00 | 0.00 | 0.00 | 0.00 | 0.00 |
| III: Moderate; GP | 5056 | 10.1% | 6.27 | 0.01 | 0.00 | 0.02 | 0.00 | 0.00 | 0.00 |
| IV: Moderate; GP, OP & elective | 7497 | 15.0% | 3.61 | 3.05 | 0.26 | 0.11 | 0.03 | 0.01 | 0.01 |
| V: Moderate; GP & emergency care | 7648 | 15.3% | 4.20 | 0.03 | 1.71 | 0.02 | 0.00 | 0.11 | 0.08 |
| VI: High; all services | 3046 | 6.1% | 8.61 | 4.15 | 2.22 | 0.24 | 0.07 | 0.38 | 0.67 |
| **Total** | **50000** | | **2.81** | **0.72** | **0.43** | **0.04** | **0.01** | **0.04** | **0.06** |

**Table S5: Median and interquartile range of presentations/length of stay over one year for each segment from 6 cluster k-means model (green indicates lower than average utilisation, red indicates higher than average utilisation)**

| k | Segment | Size | Percentage | GP | Outpatient | A&E | Elective admissions | Elective length of stay | Emergency admissions | Emergency length of stay |
|---|---|---|---|---|---|---|---|---|---|---|
| 1 | Very low | 110869 | 29.3% | 0 (0-0) | 0 (0-0) | 0 (0-0) | 0 (0-0) | 0 (0-0) | 0 (0-0) | 0 (0-0) |
| 2 | Low: GP | 98293 | 26.0% | 1 (1-2) | 0 (0-0) | 0 (0-0) | 0 (0-0) | 0 (0-0) | 0 (0-0) | 0 (0-0) |
| 3 | Moderate: GP | 67358 | 17.8% | 4 (3-6) | 0 (0-0) | 0 (0-0) | 0 (0-0) | 0 (0-0) | 0 (0-0) | 0 (0-0) |
| 4 | Moderate: GP, OP & elective | 33633 | 8.9% | 3 (2-6) | 3 (2-5) | 0 (0-0) | 0 (0-0) | 0 (0-0) | 0 (0-0) | 0 (0-0) |
| 5 | Moderate: GP & emergency | 51524 | 13.6% | 4 (2-6) | 0 (0-0) | 1 (1-2) | 0 (0-0) | 0 (0-0) | 0 (0-0) | 0 (0-0) |
| 6 | High: all services | 16632 | 4.4% | 8 (6-12) | 3 (2-6) | 2 (1-3) | 0 (0-0) | 0 (0-0) | 0 (0-1) | 0 (0-0) |
| | **Total** | **378309** | | **2 (0-4)** | **0 (0-0)** | **0 (0-0)** | **0 (0-0)** | **0 (0-0)** | **0 (0-0)** | **0 (0-0)** |



Beaney T, *et al. BMJ Open* 2021; 11:e050847. doi: 10.1136/bmjopen-2021-050847

**Table S6: Total healthcare associated costs for each segment from the 6-cluster k-means model**

| Segment | Size | Percentage | GP | | Outpatient | | A&E | | Elective admissions | | Emergency admissions | | Total | | Mean cost per capita (£) |
|---|---|---|---|---|---|---|---|---|---|---|---|---|---|---|---|
| | | | Total (£) | Percentage | Total (£) | Percentage | Total (£) | Percentage | Total (£) | Percentage | Total (£) | Percentage | Total (£) | Percentage | |
| I: Very low | 110869 | 29.3% | 0 | 0.0% | 1677749 | 3.4% | 846374 | 4.6% | 700875 | 3.6% | 315633 | 1.7% | 3540631 | 2.5% | 32 |
| II: Low; GP | 98293 | 26.0% | 4415301 | 12.7% | 1141363 | 2.3% | 768704 | 4.2% | 807133 | 4.1% | 272191 | 1.4% | 7404692 | 5.3% | 75 |
| III: Moderate; GP | 67358 | 17.8% | 11531157 | 33.3% | 1862688 | 3.8% | 0 | 0.0% | 1095476 | 5.6% | 71574 | 0.4% | 14560895 | 10.4% | 216 |
| IV: Moderate; GP, OP & elective | 33633 | 8.9% | 4689465 | 13.5% | 27200957 | 55.3% | 823913 | 4.5% | 7729103 | 39.5% | 664487 | 3.5% | 41107925 | 29.2% | 1222 |
| V: Moderate; GP & emergency care | 51524 | 13.6% | 8449386 | 24.4% | 1691922 | 3.4% | 10173249 | 55.4% | 1406429 | 7.2% | 5550515 | 29.6% | 27271501 | 19.4% | 529 |
| VI: High; all services | 16632 | 4.4% | 5567562 | 16.1% | 15639629 | 31.8% | 5748710 | 31.3% | 7832472 | 40.0% | 11904047 | 63.4% | 46692420 | 33.2% | 2807 |
| **Total** | **378309** | | **34652871** | | **49214308** | | **18360950** | | **19571488** | | **18778447** | | **140578064** | | |



Beaney T, *et al. BMJ Open* 2021; 11:e050847. doi: 10.1136/bmjopen-2021-050847

**Table S7: Population characteristics for each segment from 6-cluster k-means model**

| Participant characteristic | | I: Very low | | II: Low; GP | | III: Moderate; GP | | IV: Moderate; GP, OP & elective | | V: Moderate; GP & emergency | | VI: High; all services | |
|---|---|---|---|---|---|---|---|---|---|---|---|---|---|
| | | Total | Proportion | Total | Proportion | Total | Proportion | Total | Proportion | Total | Proportion | Total | Proportion |
| Gender | Female | 53,785 | 48.5% | 49,476 | 50.3% | 34,224 | 50.8% | 15,485 | 46.0% | 24,128 | 46.8% | 7,310 | 44.0% |
| | Male | 57,083 | 51.5% | 48,816 | 49.7% | 33,132 | 49.2% | 18,147 | 54.0% | 27,396 | 53.2% | 9,321 | 56.0% |
| | Unknown | 1 | 0.0% | 1 | 0.0% | 2 | 0.0% | 1 | 0.0% | 0 | 0.0% | 1 | 0.0% |
| Age (years) + female | Mean (SD) | 8.7 (4.0) | | 7.5 (4.3) | | 6.3 (4.8) | | 8.2 (4.6) | | 5.4 (4.7) | | 6.3 (5.1) | |
| | Median (IQR) | 9 (6-12) | | 8 (4-11) | | 6 (2-10) | | 9 (4-12) | | 4 (1-9) | | 5 (1-11) | |
| | Under 1 | 1,064 | 1.0% | 2,304 | 2.3% | 4,106 | 6.1% | 864 | 2.6% | 4,211 | 8.2% | 1,222 | 7.3% |
| | 1-2 | 2,997 | 2.7% | 5,422 | 5.5% | 5,958 | 8.8% | 1,386 | 4.1% | 4,708 | 9.1% | 1,198 | 7.2% |
| | 3-4 | 4,553 | 4.1% | 6,395 | 6.5% | 5,144 | 7.6% | 1,730 | 5.1% | 3,550 | 6.9% | 873 | 5.2% |
| | 5-10 | 25454 | 23.0% | 21398 | 21.8% | 10461 | 15.5% | 5643 | 16.8% | 7176 | 13.9% | 2076 | 12.5% |
| | 11-15 | 19,717 | 17.8% | 13,957 | 14.2% | 8,555 | 12.7% | 5,862 | 17.4% | 4,483 | 8.7% | 1,941 | 11.7% |
| Age (years) + male | Mean (SD) | 8.9 (4.0) | | 7.7 (4.3) | | 6.1 (4.6) | | 7.8 (4.6) | | 5.4 (4.6) | | 5.5 (4.8) | |
| | Median (IQR) | 9 (6-12) | | 8 (4-11) | | 5 (2-10) | | 8 (4-12) | | 4 (1-9) | | 4 (1-10) | |
| | Under 1 | 1,021 | 0.9% | 2,036 | 2.1% | 3,975 | 5.9% | 1,084 | 3.2% | 4,723 | 9.2% | 1,694 | 10.2% |
| | 1-2 | 3,038 | 2.7% | 5,075 | 5.2% | 5,698 | 8.5% | 1,921 | 5.7% | 5,398 | 10.5% | 1,790 | 10.8% |
| | 3-4 | 4,542 | 4.1% | 5,976 | 6.1% | 5,184 | 7.7% | 2,203 | 6.6% | 4,067 | 7.9% | 1,342 | 8.1% |
| | 5-10 | 25916 | 23.4% | 21542 | 21.9% | 11012 | 16.3% | 6757 | 20.1% | 8101 | 15.7% | 2486 | 14.9% |
| | 11-15 | 22,566 | 20.4% | 14,187 | 14.4% | 7,263 | 10.8% | 6,182 | 18.4% | 5,107 | 9.9% | 2,009 | 12.1% |
| Age (years) | Mean (SD) | 8.8 (4.0) | | 7.6 (4.3) | | 6.2 (4.7) | | 8.0 (4.6) | | 5.4 (4.6) | | 5.9 (4.9) | |
| | Median (IQR) | 9 (6-12) | | 8 (4-11) | | 5 (2-10) | | 8 (4-12) | | 4 (1-9) | | 5 (1-10) | |
| | 0 | 2,085 | 1.9% | 4,340 | 4.4% | 8,081 | 12.0% | 1,948 | 5.8% | 8,934 | 17.3% | 2,916 | 17.5% |
| | 1-2 | 6,035 | 5.4% | 10,498 | 10.7% | 11,656 | 17.3% | 3,307 | 9.8% | 10,106 | 19.6% | 2,988 | 18.0% |
| | 3-4 | 9,095 | 8.2% | 12,371 | 12.6% | 10,328 | 15.3% | 3,933 | 11.7% | 7,617 | 14.8% | 2,215 | 13.3% |



Beaney T, *et al. BMJ Open* 2021; 11:e050847. doi: 10.1136/bmjopen-2021-050847

| | | | | | | | | | | | | | |
|---|---|---|---|---|---|---|---|---|---|---|---|---|---|
| | 5-10 | 51371 | 46.3% | 42940 | 43.7% | 21475 | 31.9% | 12400 | 36.9% | 15277 | 29.7% | 4562 | 27.4% |
| | 11-15 | 42,283 | 38.1% | 28,144 | 28.6% | 15,818 | 23.5% | 12,045 | 35.8% | 9,590 | 18.6% | 3,951 | 23.8% |
| **Ethnicity** | Asian or Asian British | 23,778 | 21.4% | 24,124 | 24.5% | 20,215 | 30.0% | 7,749 | 23.0% | 12,893 | 25.0% | 3,945 | 23.7% |
| | White | 27,950 | 25.2% | 21,714 | 22.1% | 12,481 | 18.5% | 7,151 | 21.3% | 10,726 | 20.8% | 3,384 | 20.3% |
| | Mixed/multiple ethnic groups | 10,425 | 9.4% | 11,941 | 12.1% | 7,060 | 10.5% | 3,924 | 11.7% | 6,376 | 12.4% | 2,001 | 12.0% |
| | Other ethnic groups | 10,566 | 9.5% | 9,566 | 9.7% | 6,889 | 10.2% | 3,674 | 10.9% | 5,581 | 10.8% | 2,003 | 12.0% |
| | Black or Black British | 7,749 | 7.0% | 6,834 | 7.0% | 4,225 | 6.3% | 2,773 | 8.2% | 3,138 | 6.1% | 1,240 | 7.5% |
| | Unknown | 30401 | 27.4% | 24114 | 24.5% | 16488 | 24.5% | 8362 | 24.9% | 12810 | 24.9% | 4059 | 24.4% |
| **IMD quintile** | 1 (most deprived) | 15,282 | 13.8% | 15,462 | 15.7% | 10,670 | 15.8% | 6,600 | 19.6% | 9,101 | 17.7% | 3,391 | 20.4% |
| | 2 | 34,072 | 30.7% | 31,174 | 31.7% | 22,292 | 33.1% | 10,792 | 32.1% | 17,178 | 33.3% | 5,666 | 34.1% |
| | 3 | 29,050 | 26.2% | 25,125 | 25.6% | 17,163 | 25.5% | 7,936 | 23.6% | 13,028 | 25.3% | 3,911 | 23.5% |
| | 4 | 19,216 | 17.3% | 15,457 | 15.7% | 9,525 | 14.1% | 4,453 | 13.2% | 6,888 | 13.4% | 1,906 | 11.5% |
| | 5 (least deprived) | 8,362 | 7.5% | 7,054 | 7.2% | 4,940 | 7.3% | 2,385 | 7.1% | 3,041 | 5.9% | 931 | 5.6% |
| | Unknown | 4,887 | 4.4% | 4,021 | 4.1% | 2,768 | 4.1% | 1,467 | 4.4% | 2,288 | 4.4% | 827 | 5.0% |
| **Number of long-term conditions** | 0 | 106,464 | 96.0% | 91,248 | 92.8% | 58,453 | 86.8% | 27,083 | 80.5% | 46,206 | 89.7% | 12,457 | 74.9% |
| | One or more | 4,015 | 3.6% | 7,026 | 7.1% | 8,890 | 13.2% | 6,544 | 19.5% | 5,308 | 10.3% | 4,163 | 25.0% |
| | Missing | 390 | 0.4% | 19 | 0.0% | 15 | 0.0% | 6 | 0.0% | 10 | 0.0% | 12 | 0.1% |
| **Chronic condition*** | Asthma | 2,288 | 2.1% | 4,469 | 4.5% | 6,053 | 9.0% | 3,128 | 9.3% | 3,560 | 6.9% | 2,161 | 13.0% |
| | Obesity | 938 | 0.8% | 1605 | 1.6% | 1726 | 2.6% | 993 | 3.0% | 1139 | 2.2% | 594 | 3.6% |
| | Mental health problem | 276 | 0.2% | 435 | 0.4% | 631 | 0.9% | 756 | 2.2% | 398 | 0.8% | 397 | 2.4% |
| | Learning Disability | 84 | 0.1% | 148 | 0.2% | 394 | 0.6% | 527 | 1.6% | 133 | 0.3% | 321 | 1.9% |
| | Chronic heart disease | 245 | 0.2% | 279 | 0.3% | 237 | 0.4% | 344 | 1.0% | 186 | 0.4% | 232 | 1.4% |
| | Chronic neurological disease & epilepsy | 45 | 0.0% | 93 | 0.1% | 135 | 0.2% | 508 | 1.5% | 67 | 0.1% | 461 | 2.8% |
| | Hypothyroidism | 108 | 0.1% | 125 | 0.1% | 229 | 0.3% | 396 | 1.2% | 76 | 0.1% | 154 | 0.9% |



Beaney T, *et al. BMJ Open* 2021; 11:e050847. doi: 10.1136/bmjopen-2021-050847

| | | | | | | | | | | | | |
|---|---|---|---|---|---|---|---|---|---|---|---|---|
| | Cancer & immunosuppression | 124 | 0.1% | 152 | 0.2% | 146 | 0.2% | 315 | 0.9% | 95 | 0.2% | 229 | 1.4% |
| | Diabetes | 18 | 0.0% | 16 | 0.0% | 20 | 0.0% | 440 | 1.3% | 14 | 0.0% | 267 | 1.6% |
| **Total participants** | | **110,869** | **29.3%** | **98,293** | **26.0%** | **67,358** | **17.8%** | **33,633** | **8.9%** | **51,524** | **13.6%** | **16,632** | **4.4%** |

* For those with ≥500 with condition. Proportion of those with non-missing data on LTC

**References**

1. Aitchison K, McGeown H, Holden B, Watson M, Klaber RE, Hargreaves D. Population child health: understanding and addressing complex health needs. Arch Dis Child. 2020. doi:10.1136/archdischild-2019-317373.

2. NHS England, British Medical Association. Seasonal influenza vaccination programme clinical used for payment. 2018. https://www.google.com/url?sa=t&rct=j&q=&esrc=s&source=web&cd=&cad=rja&uact=8&ved=2ahUKEwjBybDg5PXtAhUiVBUIHR34BxcQFjAJegQIAxAC&url=https%3A%2F%2Fwww.england.nhs.uk%2Fwp-content%2Fuploads%2F2019%2F07%2Fqsr-sfl-2019-20-codes-list.xlsm&usg=AOvVaw1Mt9mxgXhT_. Accessed 30 Dec 2020.

3. Davies DL, Bouldin DW. A Cluster Separation Measure. IEEE Trans Pattern Anal Mach Intell. 1979;PAMI-1:224–7.

4. Caliński T, Harabasz J. A dendrite method for cluster analysis. Commun Stat. 1974;3:1–27. doi:10.1080/03610927408827101.

5. Rosenberg A, Hirschberg J. V-Measure: A conditional entropy-based external cluster evaluation measure. EMNLP-CoNLL 2007 - Proc 2007 Jt Conf Empir Methods Nat Lang Process Comput Nat Lang Learn. 2007; June:410–20.



Beaney T, *et al. BMJ Open* 2021; 11:e050847. doi: 10.1136/bmjopen-2021-050847
