# Supplementary material for: Patterns of healthcare utilisation in children and young people: a retrospective cohort study using routinely collected healthcare data in Northwest London
Source: BMJ Open. 2021 Dec 17;11(12):e050847. doi: 10.1136/bmjopen-2021-050847 (PMC8685945; doi:10.1136/bmjopen-2021-050847)
Supplement: Author's manuscript [file bmjopen-2021-050847.draft_revisions.pdf]

BMJ Open is committed to open peer review. As part of this commitment we make the peer review history of every article we publish publicly available.

When an article is published we post the peer reviewers' comments and the authors' responses online. We also post the versions of the paper that were used during peer review. These are the versions that the peer review comments apply to.

The versions of the paper that follow are the versions that were submitted during the peer review process. They are not the versions of record or the final published versions. They should not be cited or distributed as the published version of this manuscript.

BMJ Open is an open access journal and the full, final, typeset and author-corrected version of record of the manuscript is available on our site with no access controls, subscription charges or pay-per-view fees (http://bmjopen.bmj.com).

If you have any questions on BMJ Open's open peer review process please email info.bmjopen@bmj.com

BMJ Open

Patterns of healthcare utilisation in children and young people: a retrospective cohort study using routinely collected healthcare data in North West London

Journal:	*BMJ Open*
Manuscript ID	bmjopen-2021-050847
Article Type:	Original research
Date Submitted by the Author:	04-Mar-2021
Complete List of Authors:	Beaney, Thomas; Imperial College London, Department of Primary Care and Public Health; Imperial College London, National Institute for Health Research Applied Research Collaboration North West London<br>Clarke, Jonathan; Imperial College of Science Technology and Medicine, Department of Mathematics and Centre for Mathematics of Precision Healthcare<br>Woodcock, Thomas; Imperial College London, Department of Primary Care and Public Health; Imperial College London, National Institute for Health Research Applied Research Collaboration North West London<br>McCarthy, Rachel; North West London Collaboration of Clinical Commissioning Groups<br>Saravanakumar, Kavitha; orth West London Collaboration of Clinical Commissioning Groups<br>Barahona, Mauricio; Imperial College London, Centre for Mathematics of Precision Healthcare; Imperial College London, Department of Mathematics<br>Blair, Mitch; Imperial College London, Department of Primary Care and Public Health; Imperial College London, National Institute for Health Research Applied Research Collaboration North West London<br>Hargreaves, Dougal; Imperial College London, Primary Care and Public Health; Imperial College London, National Institute for Health Research Applied Research Collaboration North West London
Keywords:	Health informatics < BIOTECHNOLOGY & BIOINFORMATICS, HEALTH SERVICES ADMINISTRATION & MANAGEMENT, PAEDIATRICS, PUBLIC HEALTH, STATISTICS & RESEARCH METHODS

SCHOLARONE™ Manuscripts

I, the Submitting Author has the right to grant and does grant on behalf of all authors of the Work (as defined in the below author licence), an exclusive licence and/or a non-exclusive licence for contributions from authors who are: i) UK Crown employees; ii) where BMJ has agreed a CC-BY licence shall apply, and/or iii) in accordance with the terms applicable for US Federal Government officers or employees acting as part of their official duties; on a worldwide, perpetual, irrevocable, royalty-free basis to BMJ Publishing Group Ltd ("BMJ") its licensees and where the relevant Journal is co-owned by BMJ to the co-owners of the Journal, to publish the Work in this journal and any other BMJ products and to exploit all rights, as set out in our licence.

The Submitting Author accepts and understands that any supply made under these terms is made by BMJ to the Submitting Author unless you are acting as an employee on behalf of your employer or a postgraduate student of an affiliated institution which is paying any applicable article publishing charge ("APC") for Open Access articles. Where the Submitting Author wishes to make the Work available on an Open Access basis (and intends to pay the relevant APC), the terms of reuse of such Open Access shall be governed by a Creative Commons licence – details of these licences and which Creative Commons licence will apply to this Work are set out in our licence referred to above.

Other than as permitted in any relevant BMJ Author's Self Archiving Policies, I confirm this Work has not been accepted for publication elsewhere, is not being considered for publication elsewhere and does not duplicate material already published. I confirm all authors consent to publication of this Work and authorise the granting of this licence.

For peer review only

Patterns of healthcare utilisation in children and young people: a retrospective cohort study using routinely collected healthcare data in North West London

Thomas Beaney[1,2]

Jonathan M Clarke[3]

Thomas Woodcock[1,2]

Rachel McCarthy[4]

Kavitha Saravanakumar[4]

Mauricio Barahona[3]

Mitch Blair[1,2]

Dougal Hargreaves[1,2]

1. Department of Primary Care and Public Health, Imperial College London, London, W6 8RP, UK.
2. National Institute for Health Research Applied Research Collaboration North West London, Imperial College, London, W6 8RP, UK.
3. Department of Mathematics and Centre for Mathematics of Precision Healthcare, Imperial College London, London, SW7 2AZ, UK.
4. North West London Collaboration of Clinical Commissioning Groups, London, NW1 5JD, UK.

Corresponding author:

Thomas Beaney

Department of Primary Care and Public Health, Imperial College London, London, W6 8RP, UK.

Email: Thomas.beaney@imperial.ac.uk

For peer review only

Abstract (word count 300)

Objectives

With a growing role for health services in managing population health, there is a need for early identification of populations with high need. Segmentation approaches partition the population based on demographics, long-term conditions (LTCs) or healthcare utilisation but have mostly been applied to adults. Our study uses segmentation methods to distinguish patterns of healthcare utilisation in children and young people (CYP) and to explore predictors of segment membership.

Design

Retrospective cohort study.

Setting

Routinely collected primary and secondary healthcare data in North West London from the Discover database.

Participants

Individuals aged 0-15 years registered to a general practice in NW London with one full year of follow-up.

Primary and secondary outcome measures

Assignment of each participant to a segment defined by seven healthcare utilisation variables, and description of utilisation patterns by segment. Characterisation of segments and predictors of membership by age, sex, ethnicity, deprivation and presence of LTCs.

Results

378,309 children were grouped into six segments. Three segments predominantly used primary care, with varying mean utilisation. A further two moderate utilisation segments differed in use of emergency or elective care, and a high utilisation segment, representing 16,632 (4.4%) children accounted for the highest mean presentations across all activity types. The two smallest segments, together representing 13.3% of the population, accounted for 62.5% of total costs. Factors associated with membership of higher utilisation segments included younger age, residence in areas

For peer review only

of higher deprivation, and presence of one or more LTCs, but a significant proportion of variation remained unexplained.

Conclusions

This article identifies distinct patterns and predictors of healthcare utilisation in CYP. A large amount of the variation in utilisation patterns is not explained by demographics and LTCs, which may limit the use of routine data in predicting utilisation and suggests children have less well-defined trajectories of service use than adults.

Strengths and limitations of this study

- Segmentation approaches have been widely used in the adult population for the purposes of population health management, but are less widely used in children.
- This study uses routinely collected primary and secondary care data, including all eligible patients registered to general practices in North West London region.
- Using a data-driven approach with k-means clustering, we defined six distinct segments of the child population in North West London based on healthcare utilisation patterns.
- Age, sex, ethnicity, deprivation and number of long-term conditions were strong predictors of utilisation segment, but the majority of variation remained unexplained.
- Demographics and co-morbidities may not capture the variation in healthcare utilisation and further research is needed to identify whether additional factors in the electronic health record can predict utilisation in children.

For peer review only

Introduction

There is growing recognition, both internationally and within the UK, of the role of the health service in managing population health.[1–3] Population health encompasses the activities that can be taken at a system level to improve physical and mental health and wellbeing, and reduce health inequalities for a whole population, rather than focussing solely on provision of services for individual patients. Within England, the 2019 NHS Long Term Plan sets out the responsibilities expected of NHS organisations, in the form of integrated care systems, to manage and improve the health of populations.[4]

Critical to population health activities is the ability to identify and understand the needs of those for whom early intervention may improve outcomes, and thus reduce the need for more costly hospital-based care. The life course approach to prevention suggests that the long-term benefits of early intervention are greatest in children and young people (CYP), particularly in the first 1,000 days of life.[5]. Population 'segmentation' approaches have been used widely in the adult population, with the aim of identifying common care needs and designing services tailored to those in each segment to improve health outcomes and optimise healthcare utilisation.[6, 7] However, existing work on segmentation has focussed on adults, which may represent a very different population to that of CYP in terms of chronic disease burden and patterns of healthcare utilisation. A variety of approaches to population segmentation in a health setting have been described, based mostly on demographic and medical factors and including both expert-driven and data-driven designs, or a mix of the two.[8, 9] Segmenting on healthcare utilisation is an alternative, and included in some approaches, but only one previous segmentation method applied to the adult population, was based solely on utilisation.[10]

In the adult population, health care utilisation and expenditure are closely associated with increasing age and co-morbidities: in a recent study of healthcare costs in England, adults accounting for the

For peer review only

top 5% of costs were significantly older and 85.5% had at least one long-term condition (LTC).[11] In contrast, in the CYP population, a higher proportion of under-5s accounted for the top 5% of costs (compared with those aged 5-9 and 10-14 years), with only half of those in the high-cost group having no defined LTC (Punjabi, N. et al. Categorising high-cost high-need children and young people. Unpublished, under review at Archives of Diseases in Childhood). These findings suggest that approaches to segmentation using age and LTC will only partially account for the utilisation and costs associated with care in CYP.

Exploration of the link between modifiable and non-modifiable risk factors in relation to an individual's use of health services is key to developing our understanding of the utility and limitations of segmentations approaches, and the extent to which health need can be identified and intervened upon pre-emptively. The aim of this article is to use a data-driven method to characterise different patterns of healthcare utilisation over the course of a year in the CYP population for North West (NW) London, using routinely available healthcare data. We then describe the population characteristics of the resulting segments, by age, sex, ethnicity, LTC burden and deprivation and identify factors associated with membership of each segment.

Methods

Data source and cohort design

Data for this study used the Discover platform to access the Whole Systems Integrated Care (WSIC) database. WSIC holds the records of 2.4 million patients registered to general practices (GPs) within NW London, representing 95% of the registered population and comprising both primary and secondary care data.[12] Primary care data are extracted directly from the electronic health record, and are linked to secondary care data from the Secondary Uses Service (SUS) dataset of accident and emergency (A&E) attendances, inpatient admissions (including both elective and emergency admissions) and outpatient attendances covering all hospital providers in England.

For peer review only

A retrospective cohort was constructed of all currently-registered CYP with a full year (365 days) of follow-up from 01/03/2019 to 29/02/2020, inclusive, aged 0-15 years at the start of follow-up. These dates were chosen to provide contemporaneous data while minimising the overlap with the COVID-19 pandemic, due to expected changes in usual healthcare utilisation patterns. For children who had died over the study time period, 365 days of follow-up were included prior to the date of death. Further details of the cohort design and analysis methods are given in the supplementary appendix.

Healthcare utilisation, cost and LTCs

Seven healthcare utilisation variables were chosen, for which attendance is robustly recorded in the clinical record and summed across the year

1. GP attendances (including all consultation and staff types)
2. A&E attendances
3. Outpatient attendances
4. Elective hospital admissions
5. Total length of stay from elective admissions (days)
6. Emergency hospital admissions
7. Total length of stay from emergency admissions (days)

Estimated healthcare costs for primary care were derived from the Unit Costs of Health and Social Care 2019, assuming average consultation times and including direct care staff costs.[13] Secondary care costs are included directly in Discover based on the total National Tariff Payment System cost associated with each attendance or admission.[14] Where these were missing, costs were estimated based on the Unit Costs of Health and Social Care 2019 (for paediatric outpatient attendances) or the National Schedule of NHS Costs (A&E attendances and emergency/elective admissions).[15] Co-morbidities were selected from among 41 LTCs defined in Discover. A sub-set of seventeen of these

For peer review only

chronic conditions identified in previous literature as relevant to children were included [16] (appendix, Table A1), supplemented by additional SNOMED code sets for relevant conditions not included in Discover (appendix, table A2) [17] identified from the entirety of the patient primary care record. Secondary care diagnostic codes were not included in order not to bias number of conditions in favour of those presenting to secondary care, although they may be coded in the primary care record.

Cluster analysis

Two methods were considered for cluster analysis, which have been widely used in the segmentation literature: k-means and hierarchical agglomerative clustering, following the approach used by Vuik *et al* (2016).[9, 10] Given our population approach, use of the entire dataset was preferrable. Hierarchical methods are computationally intensive and may not run on datasets with several hundreds of thousands of samples and so k-means was chosen as the primary clustering method with a sensitivity compared to Ward's hierarchical agglomerative clustering on a sub-set of data.[10] Distributions of each utilisation measure were examined using histograms, were log-transformed to reduce skew and rescaled using min-max scaling (appendix, Figures 1 and 2). K-means clustering was performed, partitioning the cohort using Euclidean distance on the seven transformed and scaled utilisation variables. The algorithm was iterated 10,000 times with different randomly placed initial cluster centroids, and the iteration was selected which minimised the within-cluster sum of squares.[18] A range of models with a number of *k* clusters from 2 to 14 were compared using Davies-Bouldin (D-B) and Calinski-Harabasz (C-H) scores.[19, 20] Models with low D-B scores and high C-H scores were examined further: i) confusion matrices were constructed to compare cluster assignment in the model with *k* clusters against the model with *k+1* clusters and ii) mean values for each utilisation variable were calculated for each cluster in each model.

Statistical analysis

For the chosen clustering model, the resulting population segments were described by mean utilisation and characterised by age, sex, ethnicity, deprivation and LTC. Deprivation was calculated using quintiles of the Index of Multiple Deprivation (IMD) linked to patient postcode, with 1 representing the most deprived and 5 indicating the least deprived quintile.[21] Univariable associations were compared using chi squared tests. Adjusted multinomial logistic regression was used to identify characteristics associated with assignment to a particular segment relative to the lowest utilisation segment, including age, sex, ethnicity, deprivation and LTC in the model. The association between death and segment assignment was examined using univariable logistic regression. Missing data were low for gender (<0.1%) and IMD quintile (4.3%), but ethnicity was missing in 25.4% of cases. A sensitivity analysis of the multinomial model was carried out with the exclusion of ethnicity as a predictor.

Data were extracted from Discover using Microsoft SQL Server Management Studio 2012. Stata version 16.1 (StataCorp) was used for data manipulation, characterisation of segments and multinomial regression. Cluster analysis used Python version 3.6.8, Pandas version 1.2.0 and Scikit-learn version 0.23.2.[22]

Patient and Public Involvement

Patients or the public were not involved in this study.

Results

Population characteristics

A total of 429,496 CYP age 0-15 years registered to GPs in North West London were identified in Discover. Of these, 378,266 (88.1%) participants were currently registered and had a full year of follow-up between 01/03/2019 and 29/02/2020. An additional 43 children (0.01%) died during this

For peer review only

time period, and a full year of follow-up was included preceding the date of death. A total of 378,309 participants were therefore included in the cohort.

Characteristics of the population are shown in Table 1. 51.3% of children were male, and 48.7% female with a mean (SD) age of 7.4 (4.6) years. Ethnicity was documented for 74.6% of participants, with the most common ethnic category being Asian or Asian British (24.5%), followed by 22.0% white, 11.0% mixed, 10.1% other ethnic groups and 6.9% black. There was a higher proportion of the population falling into more deprived areas by IMD quintile, with 16.0% in the most deprived, 32.0% in the second most deprived, and only 7.1% in the least deprived quintile. 35,946 (9.5%) were recorded as having one or more LTCs, with 32,963 (91.7%) of these having one LTC and 2,983 (8.3%) having more than one LTC. Asthma was the most commonly recorded chronic condition, with 21,659 prevalent cases (5.7%), followed by obesity (6,995; 1.9%), mental health problems (2,893; 0.8%), learning disabilities (1,607; 0.4%) and chronic heart conditions (1523; 0.4%).

Healthcare utilisation

264,755 (70.0%) CYP had at least one attendance to their GP over the year, with a mean (SD) number of attendances of 2.8 (3.9), and 19,990 (5.3%) children presenting ten or more times (Table 2). A lower proportion of children attended A&E (23.5%) and outpatients (21.2%) at least once over the year. 9,461 (2.5%) children had one or more elective admissions, with a mean of length of stay of 0.3 days per admission. 87.2% of those with at least one elective admission had no overnight admissions over the year. 12,078 (3.2%) children had at least one emergency admission, with a mean length of stay of 1.6 days per admission. Of those children admitted at least once as an emergency, 41.9% had no overnight admissions over the year.

For peer review only

Model selection

Comparison of D-B and C-H scores for a range of values of *k* clusters from 2 to 14 from k-means clustering identified an optimal range from 4 to 8 clusters for further exploration (appendix, figure A3 and Table A3). Mean utilisation measures were compared for each of the clusters in the 4- to 8-cluster models (appendix figures A4-A8) and confusion matrices compared assignment to segments between clusters with increasing number of *k* clusters (appendix figures A9-A12). Membership of clusters within the 6-cluster model were relatively consistent with respect to membership of clusters in the 5- and 7-cluster models. Additional increases in number of clusters to 7 or 8 did not add to the clinical utility given the additional complexity of interpretation.

Hierarchical agglomerative clustering using Ward's method was feasible on sub-samples of up to 50,000 participants. Evaluation of cluster membership on ten different random draws of data produced a mean V-measure score (a combination of homogeneity and completeness of cluster assignment) of 0.85 suggesting clustering was sensitive to sampling (see appendix, p10).[23] Clusters were characterised for the 6-cluster model using Ward's method and were broadly similar to those from the 6-cluster k-means model (appendix, Figure A13 and Table A4). Descriptively, the both the 6-cluster k-means model and the 6-cluster hierarchical model partitioned according to the same variables, differing only in the magnitude of mean presentations, while the 'very low' utilisation group became a 'no-utilisation' group. Given the focus on describing the whole population, rather than a sub-sample, the 6-cluster k-means model was selected for further description.

Utilisation by segment

For the chosen 6-cluster model, the resulting population segments were described. Most patients fell into either a very low utilisation segment (I; 29.3%) with almost no healthcare utilisation, or into a low utilisation segment (II; 26.0%) attending a GP a mean (SD) of 1.4 (0.5) times in a year with very little additional utilisation (Table 3; medians (IQR) given in appendix Table A5). Three smaller

For peer review only

moderate utilisation segments were identified, with above average primary care utilisation. The largest of these (III), used primary care a mean (SD) of 5.2 (3.1) times per year, but had little other service use. The two remaining moderate utilisation segments were characterised by either above average elective care (IV; outpatient and elective admissions) or above average emergency care (V; A&E attendances and emergency admissions), attending primary care a mean (SD) of 4.2 (3.7) , or 5.0 (3.7) times, respectively.

A high utilisation segment (VI) was also identified, accounting for 16,632 children (4.4%), with the highest mean utilisation across all services (Table 2). Over 50% of all emergency admissions in the population, and total length of stay for elective and emergency admissions were accounted for by this group. Figure 1A shows the distribution of total activity across the seven utilisation variables for each segment. Figure 1B shows the distribution of total activity if all segments were of equal size, denoting the relative share of activity for a given individual within each segment.

Figure 1: a) Proportion of total activity for each healthcare utilisation variables accounted for by each segment: b) Relative contribution to total activity for each healthcare utilisation variable accounted for by a single individual in each segment assuming equal segment size

For peer review only

Costs per segment

Total healthcare costs for the cohort amounted to £140 million over the year. Higher utilisation segments accounted for larger per capita costs and overall costs (appendix, Table A6). Mean per capita costs ranged from £32 in segment I to £1,222 in segment IV and £2,807 in segment VI. The lowest utilisation segment (I) represented 29.3% of the population but only 2.5% of total costs, and the highest utilisation segment (VI) for 4.4% of the population but 33.2% of total costs. Segment IV also accounted for 29.2% of total costs. Costs were not evenly distributed across care type: segment V accounted for 55.4% of A&E costs but only 19.4% of total costs and segment III represented the largest proportion of GP costs (33.3%) but only 10.4% of total costs.

Predictors of segment membership

Characteristics of each segment were described by age, sex, ethnicity, IMD quintile, and LTCs (appendix Table A7). The gender balance differed across segments (p<0.001 from chi-squared) with segments IV-VI containing a higher proportion of males than females (54.0%, 53.2% and 56.0%, respectively). Higher utilisation segments tended to include younger children (p<0.001), with 17.3% and 17.5% aged under 1 year in segments V and VI, respectively, compared to only 1.9% under 1 in segment I. There was a strong association with deprivation (p<0.001) with 20.4% in the most deprived quintile and 5.6% in the least deprived in segment VI, compared to 13.8% in the most deprived and 7.5% in the least deprived in segment I. The proportion of the population with one or more LTCs increased across segments with increased utilisation (p<0.001), with only 3.6% in segment I, and 25.0% in segment VI. Children in the highest utilisation segment were significantly more likely to have died than those in the lowest utilisation segment (OR 21.4, 95% CI: 7.8 – 58.3), although 62.8% of all deaths still occurred outside the highest utilisation segment and 34.9% of deaths occurred in segments I-III.

For peer review only

Adjusted multinomial logistic regression was conducted to identify the predictors of segment membership. Relative risk ratios (RRR) are shown in Table 4. The relative risk of males being in segment VI compared to segment I, was 17% (95% CI: 12-21%) higher compared to females after adjusting for age, ethnicity, deprivation and LTC. Age was a strong predictor of segment membership, with those in higher age groups less likely to be in higher utilisation segments. Those aged 5-10 or 11-15 years had a 96% lower relative risk of being in the highest utilisation segment compared to the lowest, than those aged 0 years after adjustment (RRR 95% CI: 4-5% and 3-4%, respectively). After adjustment, those of white ethnicity were significantly less likely to be in each of the five higher utilisation segments compared to those of Asian or Asian British ethnicity.

A strong association was seen with higher levels of deprivation and membership of higher utilisation segments. Those in the least deprived quintile were 50% (RRR 95% CI: 0.46-0.55) less likely to be in segment VI compared to segment I and 41% (RRR 95% CI: 0.56-0.63) less likely to be in segment V than those in the most deprived quintile. Those with at least one LTC were 14.2 (RRR 95% CI: 13.4-15.1) times more likely to be in segment VI than segment I, 7.0 (RRR 95% CI: 6.7-7.4) times more likely to be in segment IV and 4.8 (RRR 95% CI: 4.6-5.1) times more likely to be in segment V, compared to those without any LTC. However, the majority of CYP in all segments had no documented LTC, with the highest proportion (25.0%) seen in segment VI. The pseudo-R^2 value for the model was 0.052, suggesting significant unexplained variation in segment assignment. A sensitivity analysis excluding ethnicity (due to 25.4% missing data) showed no marked differences in coefficients for the remaining variables, indicating that ethnicity was not a confounder of these relationships.

Discussion

Using a data-driven approach, we defined six segments of the CYP population in NW London each with distinct patterns of healthcare utilisation. To our knowledge, this is the first application of

For peer review only

segmentation approaches exclusive to the CYP population, including the whole population for a single geographic region. The highest utilisation group, representing less than 5% of the total population, accounted for over 50% of total emergency admissions and length of stay for the whole population. Age and deprivation were strong predictors of segment membership, with a general tendency towards lower utilisation patterns in older age groups and higher utilisation patterns in children living in areas of higher deprivation. Increased utilisation segments were associated with higher total costs, with the two smallest segments, representing 13% of the population, accounting for almost two thirds of costs.

Those with one or more LTCs were significantly more likely to be in higher-utilisation segments than those without an LTC, but only one quarter of those in the high utilisation group had an LTC. Existing segmentation approaches applied to the adult population have focussed on defining segments based on demographics and LTCs, with some incorporating healthcare utilisation.[8] Although the two approaches are closely linked in adult populations, our findings suggest they are less tightly bound in the CYP population, with three quarters in the high utilisation segment not having a recorded LTC. As a result, segmentation based on demographic factors and clinical co-morbidities may fail to identify patients with higher rates of healthcare utilisation and vice versa, indicating a less well-defined trajectory of service use in children than in adults. Although this may make identification of high-need individuals using demographics and clinical factors more difficult, it suggests the potential for a greater impact of early interventions on healthcare utilisation and long-term outcomes.

Implications for segmentation approaches in children

There are broader implications of these findings on the value of segmentation approaches in children. A limitation to the use of healthcare utilisation is that it may not accurately reflect healthcare need. A service may be indicated, but remain under-used, or may not be indicated, but

For peer review only

used frequently, influenced by factors including proximity and access to services, to the requirement and ability to pay.[24] In children, healthcare utilisation is impacted by psychosocial and mental health problems, school absence and parental anxiety and depression – factors which could not be incorporated in this study.[25, 26] Lack of access to one service can lead to a corresponding increase in utilisation of other services, with recent work indicating a higher ratio of GP and outpatient care vs emergency and inpatient care in less deprived areas (Coughlan, C *et al*. Social and ethnic group differences in healthcare use by children aged 0-14 years: a population-based cohort study in England from 2007 to 2017. Unpublished, under review at Archives of Diseases in Childhood). Furthermore, extending from describing utilisation patterns to prediction of those with higher needs in order to intervene can unintentionally reinforce pre-existing inequities in access to healthcare. Obermeyer *et al* (2019) identified significant racial bias in an algorithm identifying need based on health costs, with Black patients showing higher rates of uncontrolled comorbid illness than White patients for a given risk score, due to misattribution of reduced access to care implying lower need.[27] Many of the factors related to utilisation patterns are unlikely to be captured in quantitative data, and further qualitative evaluations exploring child and parental factors related to utilisation patterns would be beneficial.[28]

Unlike with LTC-based segmentation, in which the conditions necessary for segment assignment are by definition 'chronic', healthcare utilisation will fluctuate over time. Identifying the trajectories of movement between segments might allow for early recognition and targeted intervention to reduce the risk of transition into a higher utilisation segment. In CYP, healthcare utilisation patterns are unlikely to be static, resulting in frequent movement between utilisation segments. This is supported by our finding that assignment to lower utilisation segments is more likely with increasing age and previous research showing that fewer than one third of children accounting for the top 5% of healthcare costs in England were in the same group the following year (Punjabi, N. *et al*. Categorising high-cost high-need children and young people. Unpublished, under review at Archives of Diseases

For peer review only

in Childhood). The COVID-19 pandemic is likely to alter utilisation patterns both in the short and long term, with rates of primary and secondary care emergency attendance and PICU admissions found to have fallen significantly in Scotland.[29] Further research is needed to understand the transitions between utilisation segments and the extent to which these transitions can be predicted.

A core ambition of segmentation approaches is to identify those at greatest risk and intervene pre-emptively, but evidence for the success of such interventions is mixed, and focussed on adult populations. An intervention based around improving care coordination in the US for adults with heart failure at high risk of secondary care utilisation, demonstrated a reduction in A&E attendances and primary care visits, but not hospitalizations.[30] There is a risk in observational studies that by sampling high utilisers and following up over time, regression to the mean occurs, with over-estimation of the intervention effect. A recent randomized, controlled trial in the US targeting 'superutilizers' with care-coordination following discharge found no significant difference in readmission rates compared to usual care.[31] Within the child population, evidence is more limited. A population health management programme in the US targeted at under-21s in the top 10% of Medicaid expenditure showed a reduction in costs for those enrolled, of $378 per month per participant in the first year, but critically, showed no significant difference to a control group.[32]

In the NW London context, we are currently utilising segmentation techniques to target interventions for two specific groups of children: infants, where ED attendances and primary care utilisation have increased significantly in the last 10 years[33] and those pre-schoolers who are frequent ED attenders who make up approximately 7% of attendees but account for 40% of the workload.[34]

Limitations

For peer review only

There are a number of limitations to the generalisability of the findings presented here. The use of Discover data represents a population in a single, localised geography and the findings may not be representative of the CYP population at a national or international level. However, the population of NW London includes four of the ten most ethnically diverse local authorities in England.[35] Discover also provides comprehensive population coverage: 98% of the UK population are estimated to be registered to a GP, and 95% of those registered in NW London are captured in Discover.[12] However, it is likely that some of the most vulnerable groups, such as asylum seekers, refugees, travellers and homeless people, who may be disproportionate users of services such as emergency care, are not represented in these data.[36]

Use of linked routinely collected primary and secondary care datasets such as Discover, designed primarily for commissioning purposes and for direct clinical care, are still in their infancy for research purposes when compared with existing datasets such as the UK-based Clinical Practice Research Datalink (CPRD) and Hospital Episode Statistics (HES). Data on LTCs were collected from the primary care record, which relies on routine input from GPs. The validity of diagnostic coding in CPRD has been extensively investigated, with chronic diseases shown to be generally well-recorded.[37] Although no direct research has investigated coding validity in Discover, previous work has suggested a comparable prevalence of disease in Discover with national rates for those diseases defined by the Quality and Outcomes Framework (QOF).[12] However, many of the conditions relevant to LTCs in children do not form part of QOF, and so may be more likely to be incomplete, with evidence suggesting neurodevelopmental disorders in children, for example, are under-reported in the primary care record.[38] Obesity was prevalent in only 1.9% in our study, compared with a London average of 10.0% in reception children and 23.7% in Year 6 children, suggesting LTCs in our analysis may be under-estimated.[39]

For peer review only

The segmentation method used here was primarily data-driven; however, when using unsupervised clustering, there are no well-established criteria for choosing the optimal number of segments and a degree of subjectivity is inevitable.[9] Although metrics such as the Calinski-Harabasz and Davies-Bouldin scores, used here, give an indication as to the separation of clusters, there is no single metric which can determine the 'best' model, and different metrics may provide conflicting optima. Selection of the seven healthcare utilisation variables also impacts the cluster assignment; our model may weight inpatient admissions to a higher degree than other attendances, by including separately both elective and emergency admissions and lengths of stay. Different weightings can be applied to variables within the clustering process, to increase or decrease their relative importance, but there is no ground-truth from which to evaluate whether clusters defined in this way are better indicators of the utilisation in the population.

The defined segments are also not agnostic to the choice of clustering algorithm. Using a hierarchical approach with Ward's method on a subset of data, clusters were descriptively similar in terms of relative differences in utilisation, but with only an 85% match in terms of homogeneity and completeness. A disadvantage of k-means clustering is that changes to the number of segments can lead to changes in inclusion of previous clusters, although the six clusters appeared stable with respect to five or seven clusters, with new clusters in each case split from one or two larger clusters, lending the transition between k-means clustering outputs a quasi-hierarchical character. Use of hierarchical clustering would ensure the stability of clusters over a range of total clusters, but was not computationally feasible for the entire dataset.

Conclusion

As integrated care services focus on population health and preventive care, it is imperative that approaches are developed to identify those children and young people at greater risk. In this analysis, we describe how use of segmentation in the CYP population can identify distinct groups

For peer review only

based on different patterns of utilisation of services. Unlike in the adult population, demographics and presence of long-term conditions may not adequately explain the variation in healthcare utilisation in children. This suggests a less well-defined trajectory of healthcare utilisation in children, which may indicate greater potential for the positive impact of early interventions. Further research is needed to understand both how CYP transition between utilisation segments, and whether additional factors available in routine electronic health records can explain variation in utilisation, to enable early identification and intervention.

Acknowledgements

Many thanks to Eamon O'Doherty for his support with Discover access and data queries.

Ethics approval

Access to the Discover dataset was granted by the North West London Data Access Sub-group on 17/09/2020. Ethical approval was not required for this study as work was carried out for the purposes of service evaluation.

Funding

TB is supported by a National Institute for Health Research (NIHR) Academic Clinical Fellowship and by the NIHR Applied Research Collaboration (ARC) programme for NW London. JC and M Barahona acknowledge support from EPSRC grant EP/N014529/1 supporting the EPSRC Centre for Mathematics of Precision Healthcare. JC is also supported by the Wellcome Trust. [215938/Z/19/Z]. TW is supported by the NIHR ARC programme for NW London. The views expressed in this publication are those of the authors and not necessarily those of the NHS, the NIHR or the Department of Health.

Competing interests

For peer review only

The authors have no competing interests to declare.

Contributorship statement

TB, JC, M Blair and DSH conceived the study. TB, JC, TW, M Barahona, M Blair and DSH were involved in the design and methodology of the study. TB conducted the cohort extraction and statistical analysis on Discover. TB and JC conducted the cluster analysis. TB wrote the first draft of the manuscript. All authors contributed to critical revisions of the manuscript and approved the final submission.

Data sharing statement

Data for this study is not publicly available, but access to pseudonymised data may be accessible for research purposes or service evaluation through application to the North West London Data Access Sub-Group.

References

1. Buck D, Baylis A, Dougall D, Robertson R. A vision for population health: Towards a healthier future. 2018. https://www.kingsfund.org.uk/publications/vision-population-health. Accessed 19 Dec 2020.

2. Gourevitch MN, Curtis LH, Durkin MS, Fagerlin A, Gelijns AC, Platt R, et al. The Emergence of Population Health in US Academic Medicine: A Qualitative Assessment. JAMA Netw Open. 2019;2:e192200–e192200. doi:10.1001/jamanetworkopen.2019.2200.

3. Kindig D, Stoddart G. What is population health? Am J Public Health. 2003;93:380–3.

4. NHS England. The NHS Long Term Plan. 2019. https://www.longtermplan.nhs.uk/wp-content/uploads/2019/08/nhs-long-term-plan-version-1.2.pdf. Accessed 6 Sep 2019.

5. Public Health England. Health matters: giving every child the best start in life. 2016. https://www.gov.uk/government/publications/health-matters-giving-every-child-the-best-start-in-

For peer review only

life/health-matters-giving-every-child-the-best-start-in-life. Accessed 28 Jan 2021.

6. Brommels M. Patient Segmentation: Adjust the Production Logic to the Medical Knowledge Applied and the Patient's Ability to Self-Manage—A Discussion Paper. Front Public Heal. 2020;8:195. doi:10.3389/fpubh.2020.00195.

7. Yoon S, Goh H, Kwan YH, Thumboo J, Low LL. Identifying optimal indicators and purposes of population segmentation through engagement of key stakeholders: a qualitative study. Heal Res Policy Syst. 2020;18:26. doi:10.1186/s12961-019-0519-x.

8. Chong JL, Lim KK, Matchar DB. Population segmentation based on healthcare needs: a systematic review. Syst Rev. 2019;8:202. doi:10.1186/s13643-019-1105-6.

9. Yan S, Kwan YH, Tan CS, Thumboo J, Low LL. A systematic review of the clinical application of data-driven population segmentation analysis. BMC Med Res Methodol. 2018;18:121. doi:10.1186/s12874-018-0584-9.

10. Vuik SI, Mayer E, Darzi A. A quantitative evidence base for population health: applying utilization-based cluster analysis to segment a patient population. Popul Health Metr. 2016;14:44. doi:10.1186/s12963-016-0115-z.

11. Dreyer K, Parry W, Jayatunga W, Deeny S. A descriptive analysis of health care use by high cost, high need patients in England. 2019. https://www.health.org.uk/publications/a-descriptive-analysis-of-health-care-use-by-high-cost-high-need-patients-in-england. Accessed 19 Dec 2020.

12. Bottle A, Cohen C, Lucas A, Saravanakumar K, Ul-Haq Z, Smith W, et al. How an electronic health record became a real-world research resource: comparison between London's Whole Systems Integrated Care database and the Clinical Practice Research Datalink. BMC Med Inform Decis Mak. 2020;20:71.

13. Curtis LA, Burns A. Unit Costs of Health and Social Care 2019. PSSRU, University of Kent; 2019. https://kar.kent.ac.uk/79286/.

14. NHS Improvement. Developing the national tariff. 2020.

15. NHS England. National Cost Collection: National Schedule of NHS costs - Year 2018-19 - NHS trust

For peer review only

and NHS foundation trusts. National Cost Collection for the NHS. 2019.

16. Aitchison K, McGeown H, Holden B, Watson M, Klaber RE, Hargreaves D. Population child health: understanding and addressing complex health needs. Arch Dis Child. 2020. doi:10.1136/archdischild-2019-317373.

17. NHS England, British Medical Association. Seasonal influenza vaccination programme clinical used for payment. 2018. https://www.google.com/url?sa=t&rct=j&q=&esrc=s&source=web&cd=&cad=rja&uact=8&ved=2ahUKEwjBybDg5PXtAhUiVBUIHR34BxcQFjAJegQIAxAC&url=https%3A%2F%2Fwww.england.nhs.uk%2Fwp-content%2Fuploads%2F2019%2F07%2Fqsr-sfl-2019-20-codes-list.xlsm&usg=AOvVaw1Mt9mxgXhT_. Accessed 30 Dec 2020.

18. 2.3. Clustering — scikit-learn 0.24.1 documentation. https://scikit-learn.org/stable/modules/clustering.html#k-means. Accessed 29 Jan 2021.

19. Davies DL, Bouldin DW. A Cluster Separation Measure. IEEE Trans Pattern Anal Mach Intell. 1979;PAMI-1:224–7.

20. Caliński T, Harabasz J. A dendrite method for cluster analysis. Commun Stat. 1974;3:1–27. doi:10.1080/03610927408827101.

21. Ministry of Housing Communities & Local Government. English indices of deprivation 2015. National Statistics. 2015. https://www.gov.uk/government/statistics/english-indices-of-deprivation-2015. Accessed 23 Feb 2021.

22. Pedregosa F, Varoquaux G, Gramfort A, Michel V, Thirion B, Grisel O, et al. Scikit-learn: Machine Learning in Python. J Mach Learn Res. 2011;12:2825–30. http://jmlr.org/papers/v12/pedregosa11a.html.

23. Rosenberg A, Hirschberg J. V-Measure: A conditional entropy-based external cluster evaluation measure. EMNLP-CoNLL 2007 - Proc 2007 Jt Conf Empir Methods Nat Lang Process Comput Nat Lang Learn. 2007; June:410–20.

24. National Academies of Sciences Engineering and Medicine. Health-Care Utilization as a Proxy in

For peer review only

Disability Determination. In: Health-Care Utilization as a Proxy in Disability Determination. Washington, DC: The National Academies Press; 2018.

25. Al-Saffar MN, Hayhoe BW, Harris MJ, Majeed A, Greenfield G. Children as frequent attenders in primary care: a systematic review. BJGP open. 2020;4.

26. Dreyer K, Williamson RAP, Hargreaves DS, Rosen R, Deeny SR. Associations between parental mental health and other family factors and healthcare utilisation among children and young people: a retrospective, cross-sectional study of linked healthcare data. BMJ Paediatr open. 2018;2:e000266.

27. Obermeyer Z, Powers B, Vogeli C, Mullainathan S. Dissecting racial bias in an algorithm used to manage the health of populations. Science (80-). 2019;366:447–53. doi:10.1126/science.aax2342.

28. Neill SJ. Containing acute childhood illness within family life: a substantive grounded theory. J child Heal care Prof Work with Child Hosp community. 2010;14:327–44.

29. Williams TC, MacRae C, Swann O V, Haseeb H, Cunningham S, Davies P, et al. Indirect effects of the COVID-19 pandemic on paediatric healthcare use and severe disease: a retrospective national cohort study. Arch Dis Child. 2021. doi:10.1136/archdischild-2020-321008.

30. David G, Smith-McLallen A, Ukert B. The effect of predictive analytics-driven interventions on healthcare utilization. J Health Econ. 2019;64:68–79. doi:https://doi.org/10.1016/j.jhealeco.2019.02.002.

31. Finkelstein A, Zhou A, Taubman S, Doyle J. Health Care Hotspotting — A Randomized, Controlled Trial. N Engl J Med. 2020;382:152–62. doi:10.1056/NEJMsa1906848.

32. Peele P, Keyser D, Lovelace J, Moss D. Advancing Value-Based Population Health Management Through Payer-Provider Partnerships: Improving Outcomes for Children With Complex Conditions. J Healthc Qual Off Publ Natl Assoc Healthc Qual. 2018;40:e26–32.

33. Ruzangi J, Blair M, Cecil E, Greenfield G, Bottle A, Hargreaves DS, et al. Trends in healthcare use in children aged less than 15 years: a population-based cohort study in England from 2007 to 2017. BMJ Open. 2020;10. doi:10.1136/bmjopen-2019-033761.

34. Blair M, Poots AJ, Lim V, Hiles S, Greenfield G, Crehan C, et al. Preschool children who are

For peer review only

frequent attenders in emergency departments: an observational study of associated demographics and clinical characteristics. Arch Dis Child. 2018;103:19–23. doi:10.1136/archdischild-2016-311952.

35. Office for National Statistics. Regional ethnic diversity. https://www.ethnicity-facts-figures.service.gov.uk/uk-population-by-ethnicity/national-and-regional-populations/regional-ethnic-diversity/latest#ethnic-diversity-by-area. Accessed 14 Aug 2020.

36. Aspinall PJ. Hidden Needs: Identifying Key Vulnerable Groups in Data Collections: Vulnerable Migrants, Gypsies and Travellers, Homeless People, and Sex Workers. Incl Heal. 2014;:1–132. https://www.gov.uk/government/uploads/system/uploads/attachment_data/file/287805/vulnerable_groups_data_collections.pdf.

37. Khan NF, Harrison SE, Rose PW. Validity of diagnostic coding within the General Practice Research Database: a systematic review. Br J Gen Pract. 2010;60:e128--e136. doi:10.3399/bjgp10X483562.

38. Charlton RA, McGrogan A, Snowball J, Yates LM, Wood A, Clayton-Smith J, et al. Sensitivity of the UK Clinical Practice Research Datalink to Detect Neurodevelopmental Effects of Medicine Exposure in Utero: Comparative Analysis of an Antiepileptic Drug-Exposed Cohort. Drug Saf. 2017;40:387–97. doi:10.1007/s40264-017-0506-5.

39. Public Health England. Obesity Profile. Public Health Profiles. 2020.

For peer review only

Table 1: Participant demographics, and long-term conditions at recruitment for all 387,013 participants

Participant characteristic		Total	Percentage
**Gender**	Female	184,408	48.7%
	Male	193,895	51.3%
	Unknown	6	<0.1%
**Age (years)**	Mean (SD)	7.4 (4.6)	
	Median (IQR)	7 (3-11)	
	0	28,304	7.5%
	1-2	44,590	11.8%
	3-4	45,559	12.0%
	5-10	148025	39.1%
	11-15	111,831	29.6%
**Ethnicity**	Asian or Asian British	92,704	24.5%
	White	83,406	22.0%
	Mixed/multiple ethnic groups	41,727	11.0%
	Other ethnic groups	38,279	10.1%
	Black or Black British	25,959	6.9%
	Unknown	96234	25.4%
**IMD quintile**	1 (most deprived)	60,506	16.0%
	2	121,174	32.0%
	3	96,213	25.4%
	4	57,445	15.2%
	5 (least deprived)	26,713	7.1%
	Unknown	16,258	4.3%
**Number of long-term conditions (LTC)**	0	341,911	90.4%
	1	32,963	8.7%
	2	2,724	0.7%
	3	235	0.1%
	4 or more	24	<0.1%
	Missing data	452	0.1%
**Long-term conditions***	Asthma	21,659	5.7%
	Obesity	6995	1.9%
	Mental health problem	2893	0.8%
	Learning Disability	1607	0.4%
	Chronic heart disease	1523	0.4%
	Chronic neurological disease & epilepsy	1309	0.3%
	Hypothyroidism	1088	0.3%
	Cancer & immunosuppression	1061	0.3%
	Diabetes	775	0.2%
	Chronic respiratory disease (non-asthma)	126	<0.1%
	Chronic kidney disease	126	<0.1%
	Palliative care	36	<0.1%
	Rheumatoid arthritis	18	<0.1%
**Total participants**		**378,309**	

* Proportion of those with non-missing data on LTC

For peer review only

Table 2: Healthcare utilisation over one year for all 378,309 participants

Healthcare utilisation		Total	Percentage
**GP attendances**	Mean (SD)	2.8 (3.9)	
	Median (IQR)	2 (0-4)	
	0	113,554	30.0%
	1	70,666	18.7%
	2-4	116,328	30.7%
	5-9	57,771	15.3%
	10+	19,990	5.3%
**A&E attendances**	Mean (SD)	0.4 (1.0)	
	Median (IQR)	0 (0-0)	
	0	289,316	76.5%
	1	51,972	13.7%
	2-4	33,022	8.7%
	5-9	3,724	1.0%
	10+	275	0.1%
**Outpatient attendances**	Mean (SD)	0.7 (2.3)	
	Median (IQR)	0 (0-0)	
	0	298,289	78.8%
	1	28,437	7.5%
	2-4	35,014	9.3%
	5-9	12,448	3.3%
	10+	4,121	1.1%
**Elective admissions**	Mean (SD)	0.04 (0.40)	
	Median (IQR)	0 (0-0)	
	0	368,848	97.5%
	1-2	7,498	2.0%
	3-4	1,709	0.5%
	5-9	158	<0.1%
	10+	96	<0.1%
**Total elective length of stay (days) ^**	Mean (SD)	0.24 (0.98)	
	Median (IQR)	0 (0-0)	
	0	8,248	87.2%
	1	822	8.7%
	2-4	256	2.7%
	5-9	76	0.8%
	10+	59	0.6%
**Emergency admissions**	Mean (SD)	0.04 (0.28)	
	Median (IQR)	0 (0-0)	
	0	366,231	96.8%
	1-2	10,054	2.7%
	3-4	1,902	0.5%
	5-9	108	<0.1%
	10+	14	<0.1%

For peer review only

	Mean (SD)	1.6 (4.2)	
	Median (IQR)	1 (0-2)	
**Total emergency length of stay (days) ^**	0	5,058	41.9%
	1-2	3,778	31.3%
	3-4	2,302	19.1%
	5-9	647	5.4%
	10-14	293	2.4%
**Total participants**		**378,309**	

* Proportion of those with non-missing data on LTC

^ percentages of those admitted at least once

For peer review only

Table 3: Mean presentations/length of stay over one year for each segment from 6 cluster k-means model (green indicates lower than average utilisation, red indicates higher than average utilisation)

Segment	Size	Percentage	GP	Outpatient	A&E	Elective admissions	Elective length of stay	Emergency admissions	Emergency length of stay
I: Very low	110869	29.3%	0.00	0.09	0.07	0.01	0.00	0.00	0.00
II: Low; GP	98293	26.0%	1.36	0.06	0.07	0.01	0.00	0.00	0.00
III: Moderate; GP	67358	17.8%	5.19	0.14	0.00	0.01	0.00	0.00	0.00
IV: Moderate; GP, OP & elective	33633	8.9%	4.23	4.47	0.22	0.15	0.03	0.01	0.01
V: Moderate; GP & emergency	51524	13.6%	4.97	0.17	1.75	0.02	0.00	0.11	0.08
VI: High; all services	16632	4.4%	10.14	5.01	2.78	0.32	0.14	0.52	0.89
**Total**	**378309**		**2.78**	**0.71**	**0.42**	**0.04**	**0.01**	**0.04**	**0.05**

Table 4: Adjusted multinomial logistic regression of factors associated with segment assignment for 269,248 participants with non-missing data

Segment		Relative risk ratio	Standard error	z-score	p-value	95% confidence interval	
I; Very low	**Gender**	Base outcomes					
	Female						
	**Age (years)**						
	0						
	**Ethnicity**						
	Asian or Asian British						
	**IMD quintile**						
	1						
	**Long term conditions**						
	None						
II: Low; GP	**Gender**						
	Male	0.93	0.01	-7.29	0.00	0.91	0.95
	**Age (years)**						
	1-2	0.90	0.04	-2.75	0.01	0.83	0.97
	3-4	0.65	0.03	-11.13	0.00	0.60	0.70
	5-10	0.39	0.01	-26.36	0.00	0.37	0.42
	11-15	0.30	0.01	-33.68	0.00	0.28	0.32
	**Ethnicity**						
	White	0.74	0.01	-22.10	0.00	0.73	0.76
	Mixed	1.06	0.02	3.20	0.00	1.02	1.09
	Other ethnic groups	0.83	0.01	-10.52	0.00	0.80	0.86
	Black or Black British	0.85	0.02	-8.10	0.00	0.82	0.88
	**IMD quintile**						
	2	0.89	0.01	-7.45	0.00	0.86	0.91
	3	0.83	0.01	-11.27	0.00	0.80	0.85
	4	0.78	0.01	-13.33	0.00	0.75	0.81
	5	0.82	0.02	-8.10	0.00	0.79	0.86
	**Long term conditions**						
	One or more	2.29	0.05	35.17	0.00	2.19	2.40
	Constant	2.96	0.11	28.26	0.00	2.74	3.19
III: Moderate; GP	**Gender**						
	Male	0.91	0.01	-8.23	0.00	0.88	0.93
	**Age (years)**						
	1-2	0.50	0.02	-18.60	0.00	0.46	0.54
	3-4	0.26	0.01	-36.82	0.00	0.24	0.28

For peer review only

	5-10	0.09	0.00	-73.43	0.00	0.08	0.09
	11-15	0.07	0.00	-78.84	0.00	0.06	0.07
	**Ethnicity**						
	White	0.49	0.01	-45.98	0.00	0.48	0.51
	Mixed	0.70	0.01	-18.41	0.00	0.67	0.73
	Other ethnic groups	0.67	0.01	-20.45	0.00	0.64	0.70
	Black or Black British	0.63	0.01	-19.98	0.00	0.60	0.66
	**IMD quintile**						
	2	0.88	0.02	-6.80	0.00	0.85	0.91
	3	0.77	0.01	-13.37	0.00	0.74	0.80
	4	0.67	0.01	-18.28	0.00	0.64	0.70
	5	0.82	0.02	-7.37	0.00	0.77	0.86
	**Long term conditions**						
	One or more	5.80	0.13	75.59	0.00	5.54	6.07
	Constant	8.00	0.29	56.48	0.00	7.44	8.59
**IV: Moderate; GP, OP & elective**	**Gender**						
	Male	1.08	0.02	5.00	0.00	1.05	1.11
	**Age (years)**						
	1-2	0.57	0.03	-11.84	0.00	0.51	0.62
	3-4	0.41	0.02	-19.52	0.00	0.37	0.44
	5-10	0.20	0.01	-38.29	0.00	0.19	0.22
	11-15	0.21	0.01	-37.12	0.00	0.19	0.23
	**Ethnicity**						
	White	0.78	0.02	-12.86	0.00	0.75	0.81
	Mixed	1.05	0.03	1.96	0.05	1.00	1.10
	Other ethnic groups	0.96	0.02	-1.66	0.10	0.92	1.01
	Black or Black British	0.96	0.03	-1.30	0.19	0.91	1.02
	**IMD quintile**						
	2	0.73	0.02	-14.28	0.00	0.70	0.77
	3	0.64	0.01	-19.54	0.00	0.61	0.67
	4	0.55	0.01	-22.59	0.00	0.52	0.58
	5	0.69	0.02	-11.10	0.00	0.65	0.74
	**Long term conditions**						
	One or more	7.01	0.17	78.05	0.00	6.67	7.36
	Constant	1.53	0.07	9.31	0.00	1.40	1.68
**V: Moderate; GP & emergency**	**Gender**						
	Male	1.06	0.01	4.13	0.00	1.03	1.08
	**Age (years)**						
	1-2	0.39	0.01	-25.17	0.00	0.36	0.42
	3-4	0.18	0.01	-46.40	0.00	0.17	0.20
	5-10	0.06	0.00	-84.94	0.00	0.06	0.06

For peer review only

	11-15	0.04	0.00	-92.34	0.00	0.04	0.04
	**Ethnicity**						
	White	0.66	0.01	-24.96	0.00	0.63	0.68
	Mixed	0.97	0.02	-1.70	0.09	0.93	1.01
	Other ethnic groups	0.81	0.02	-9.64	0.00	0.78	0.85
	Black or Black British	0.73	0.02	-12.05	0.00	0.70	0.77
	**IMD quintile**						
	2	0.80	0.02	-11.67	0.00	0.77	0.83
	3	0.69	0.01	-17.75	0.00	0.67	0.72
	4	0.56	0.01	-24.17	0.00	0.54	0.59
	5	0.59	0.02	-16.85	0.00	0.56	0.63
	**Long term conditions**						
	One or more	4.84	0.12	61.75	0.00	4.61	5.09
	Constant	7.59	0.28	54.18	0.00	7.06	8.17
**VI: High; all services**	**Gender**						
	Male	1.17	0.02	7.55	0.00	1.12	1.21
	**Age (years)**						
	1-2	0.34	0.02	-23.77	0.00	0.31	0.37
	3-4	0.13	0.01	-42.88	0.00	0.12	0.15
	5-10	0.04	0.00	-76.73	0.00	0.04	0.05
	11-15	0.04	0.00	-77.55	0.00	0.03	0.04
	**Ethnicity**						
	White	0.69	0.02	-14.05	0.00	0.65	0.73
	Mixed	0.97	0.03	-0.89	0.38	0.91	1.03
	Other ethnic groups	0.91	0.03	-2.87	0.00	0.86	0.97
	Black or Black British	0.85	0.03	-4.20	0.00	0.79	0.92
	**IMD quintile**						
	2	0.72	0.02	-11.83	0.00	0.68	0.76
	3	0.58	0.02	-17.84	0.00	0.55	0.62
	4	0.43	0.02	-23.07	0.00	0.40	0.46
	5	0.50	0.02	-14.11	0.00	0.46	0.55
	**Long term conditions**						
	One or more	14.23	0.42	90.16	0.00	13.44	15.08
	Constant	2.55	0.12	19.84	0.00	2.32	2.79

For peer review only

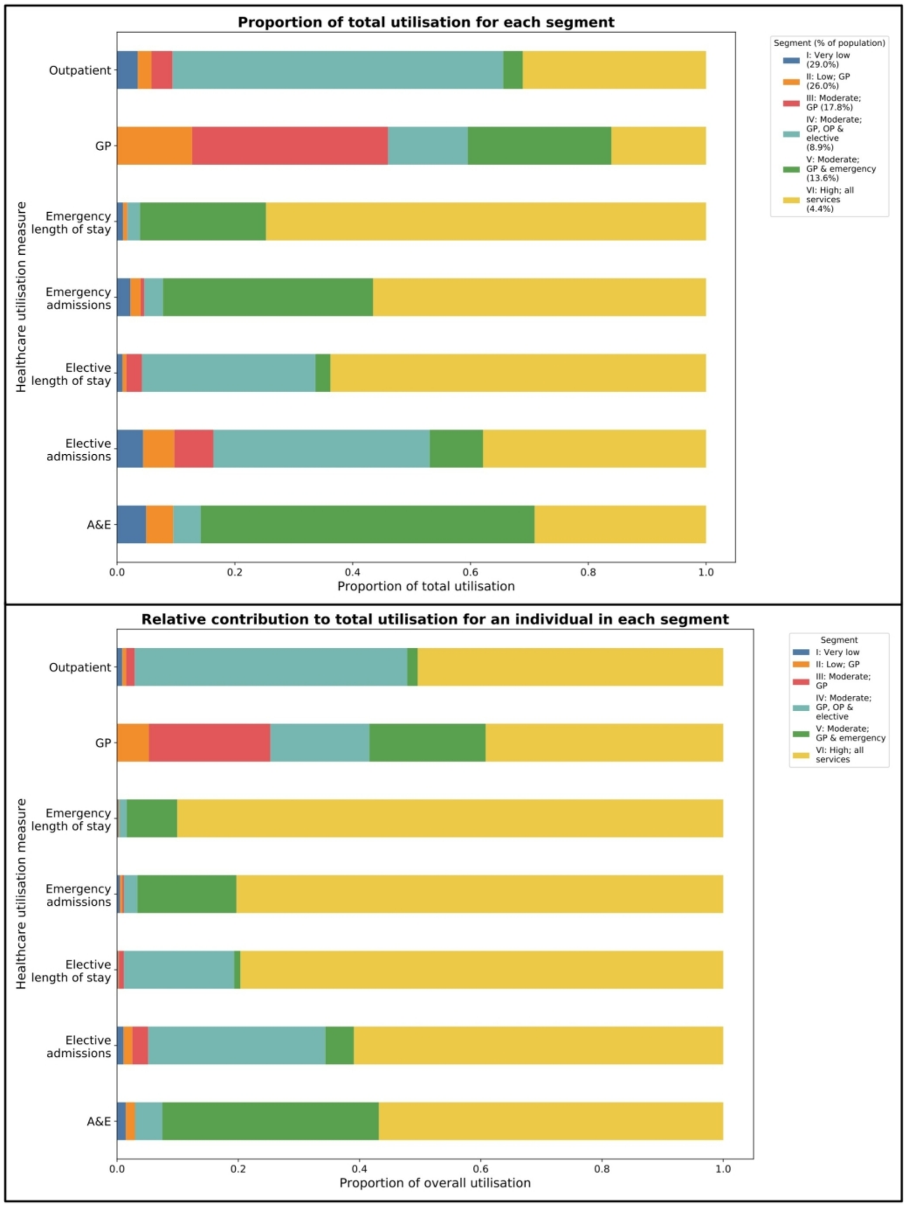

Figure 1: a) Proportion of total activity for each healthcare utilisation variables accounted for by each segment: b) Relative contribution to total activity for each healthcare utilisation variable accounted for by a single individual in each segment assuming equal segment size

160x213mm (300 x 300 DPI)

Patterns of healthcare utilisation in children and young people: a retrospective cohort study using routinely collected healthcare data in North West London

Thomas Beaney[1,2]

Jonathan M Clarke[3]

Thomas Woodcock[1,2]

Rachel McCarthy[4]

Kavitha Saravanakumar[4]

Mauricio Barahona[3]

Mitch Blair[1,2]

Dougal Hargreaves[1,2]

1. Department of Primary Care and Public Health, Imperial College London, London, W6 8RP, UK.
2. National Institute for Health Research Applied Research Collaboration North West London, Imperial College, London, W6 8RP, UK.
3. Department of Mathematics and Centre for Mathematics of Precision Healthcare, Imperial College London, London, SW7 2AZ, UK.
4. North West London Collaboration of Clinical Commissioning Groups, London, NW1 5JD, UK

Corresponding author:

Thomas Beaney

Department of Primary Care and Public Health, Imperial College London, London, W6 8RP, UK.

Email: Thomas.beaney@imperial.ac.uk

For peer review only

Appendix

Patterns of healthcare utilisation in children and young people: a retrospective cohort study using routinely collected healthcare data in North West London

Cohort design

Data for this study uses the Discover platform to access the Whole Systems Integrated Care (WSIC), database combining primary and secondary care data.[1] A cohort was constructed from the Discover database of children and young people (CYP) aged 0-15 years at time of study recruitment with a full year of follow-up. Participants with less than one full year of data were not included, in order to account for seasonal changes in pattern of presentation. A study end date of 29/02/2020 was selected to include recent data while minimising the impact of the COVID-19 pandemic on expected changes to healthcare utilisation.

The study recruitment period was defined between 01/03/2018 and 01/03/2019, with a full year (365 days) of follow-up. All patients currently registered were recruited on 01/03/2019 with follow-up data included up until 29/02/2020, inclusive. For children who died between 01/03/2019 and 29/02/2020, a year of data were included preceding the recorded date of death. Due to data pseudonymisation, date of birth on Discover is not available, and age is given in whole years as of the date of last data refresh. Age used in analysis was calculated as the estimated age at the date of study recruitment (age + 6 months – (number of days from date of recruitment to date of data refresh).

For peer review only - http://bmjopen.bmj.com/site/about/guidelines.xhtml

Healthcare utilisation

Seven healthcare utilisation measures were selected for inclusion:

1. GP attendances
2. A&E attendances
3. Outpatient attendances
4. Elective hospital admissions
5. Total length of stay from elective admissions in days
6. Emergency hospital admissions
7. Total length of stay from emergency admissions in days

Numbers of attendances or admissions were summed across the year. GP attendances included all consultation types collectively (eg. face to face, telephone, home visits) and staff types (eg. GP, nurse) due to variable recording of these factors in the Discover dataset, with exclusion of events linked only to administrative Read codes, and includes visits for preventive reasons. Where more than one event of the same type occurred on a single date, only one attendance was counted. Total length of stay was calculated as the total number of inpatient days across the year, separately for elective and emergency admissions. Where a patient was admitted and discharged on the same day, the length of stay for that admission is recorded as zero days.

Distributions of healthcare utilisation were heavily skewed as shown in Figure 1, with the majority of patients having very few presentations, and a small number having very many. K-means clustering works. Variables were log-transformed prior to min-max scaling which reduced skew (Figure A2).

For peer review only

Figure A1: Histograms displaying distributions of each healthcare utilisation variable

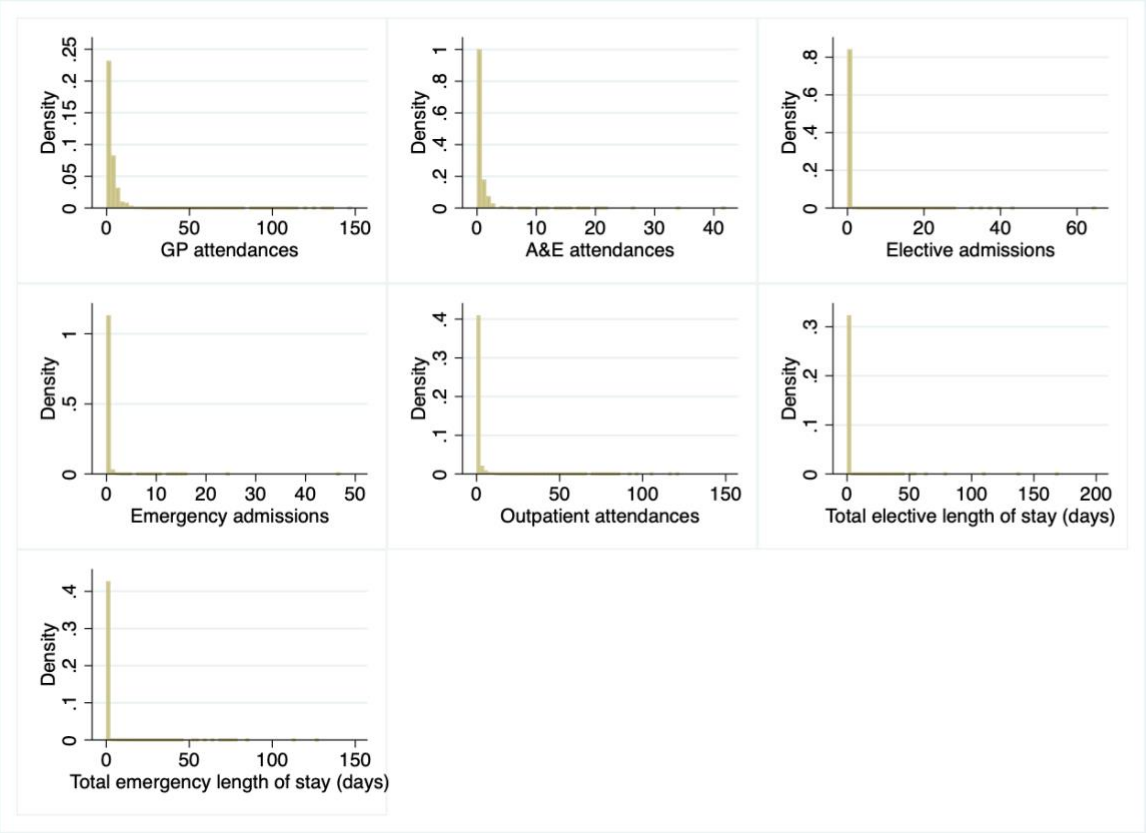

Figure A2: Histograms displaying distributions of each healthcare utilisation variable after log transformation and min-max scaling

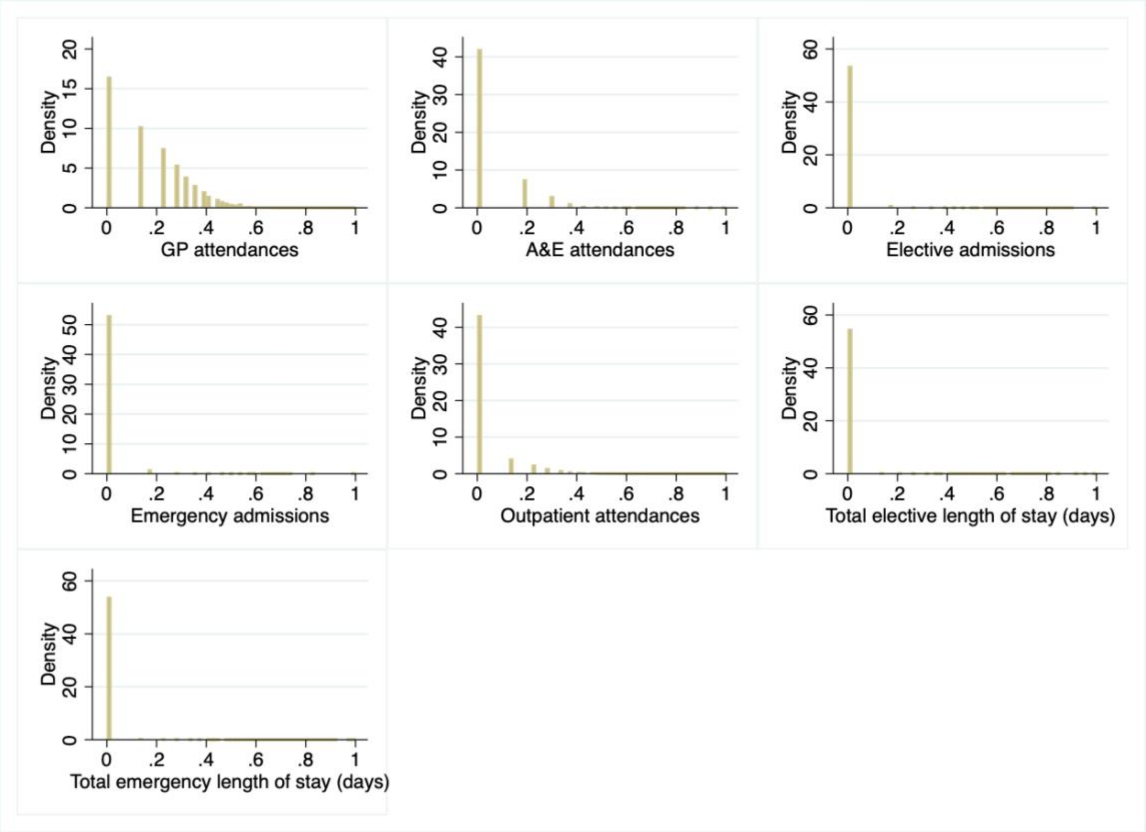

Long-term conditions

Discover includes a long-term condition (LTC) table, listing 41 chronic conditions based on Read V2 codes. A sub-set of 17 of these were selected for inclusion based on Aitchison *et al* (2020), who identified relevant LTCs from Discover applicable to children (Table A1).[2] Certain important diagnostic categories, such as congenital heart disease are not included in the Discover LTC list and so additional diagnosis codes were sourced. Raw Read codes are translated into SNOMED in the GP events table on Discover. Additional SNOMED code clusters were extracted for asthma, obesity, congenital heart disease, chronic kidney disease, diabetes, immunosuppression, learning disability, liver disease, neurological disease and chronic respiratory disease based on available code clusters listed by NHS as risk factors for influenza vaccination.[3] Chronic heart disease codes were combined with coronary heart disease and heart failure, and chronic neurological disease was combined with cerebral palsy, epilepsy and spina bifida (Table A2). Number of LTCs were counted as the number of categories of LTC from Table A3. Relevant codes were identified from the entirety of the patient primary care record and secondary care diagnostic codes were not included in order not to bias number of conditions in favour of those presenting to secondary care.

For peer review only

Table A1: 41 Long-term conditions identified in Discover (included conditions highlighted)

**Asthma ***	**Hypothyroidism ***
Atrial Fibrillation	**Anxiety ***
**Cancer ***	Charcot Marie Tooth Disease
**CKD ***	Dystonia Primary Idiopathic
COPD	Essential Tremor
Dementia	Huntingtons Disease
**Depression ***	Motor Neurone Disease
**Diabetes ***	Multiple Sclerosis
**Epilepsy ***	Multiple System Atrophy
**Heart Failure ***	Muscular Dystrophy
Hypertension	Myalgic Encephalopmyelitis
**Learning Disability ***	Myasthenia Gravis
**Mental Health ***	Parkinsons Disease
**Obesity ***	Progressive Supranuclear Palsy
Osteoporosis	Transverse Myelitis
Peripheral Arterial Disease	**Cerebal Palsy ***
**Palliative care ***	**Spina Bifida and Hydrocephalus ***
**Rheumatoid Arthritis ***	Ataxia
**Coronary Heart Disease ***	Ataxia-Telangiectasia
Stroke/TIA	Encephalitis
Ischaemic Heart Disease	

* Included in analysis

Table A2: Defined long-term conditions and source of codes from Discover and SNOMED

Long-term condition (LTC)	Discover LTC table	Additional SNOMED clusters[3]
Asthma	Asthma	Asthma
Obesity	Obesity	Obesity
Mental health condition	Anxiety; depression; mental health	-
Learning Disability	Learning disability	Learning disability
Chronic heart disease	Coronary heart disease; heart failure	Chronic heart disease
Chronic neurological disease & epilepsy	Cerebral palsy; epilepsy; spina bifida	Chronic neurological disease
Hypothyroidism	Hypothyroidism	-
Cancer & immunosuppression	Cancer	Immunosuppression
Diabetes	Diabetes	Diabetes
Chronic respiratory disease (non-asthma)	-	Chronic respiratory disease
Chronic kidney disease	Chronic kidney disease	Chronic kidney disease
Palliative care	Palliative care	-
Rheumatoid arthritis	Rheumatoid arthritis	-

For peer review only

Healthcare costs

Total healthcare costs over the year were summed across all activity types. Estimated costs for primary care were derived from the Unit Costs of Health and Social Care 2019, assuming average consultation times and including direct care staff costs.[4] Primary care prescription costs are not included. Secondary care costs are included directly in Discover based on the total National Tariff Payment System cost associated with each attendance or admission.[5] Where these were missing, costs were estimated based on the Unit Costs of Health and Social Care 2019 (for paediatric outpatient attendances) or the National Schedule of NHS Costs (A&E attendances and emergency/elective admissions).[6]

For peer review only

Selection of number of clusters

Figure A3: Elbow plot of cost function against a range of values of k

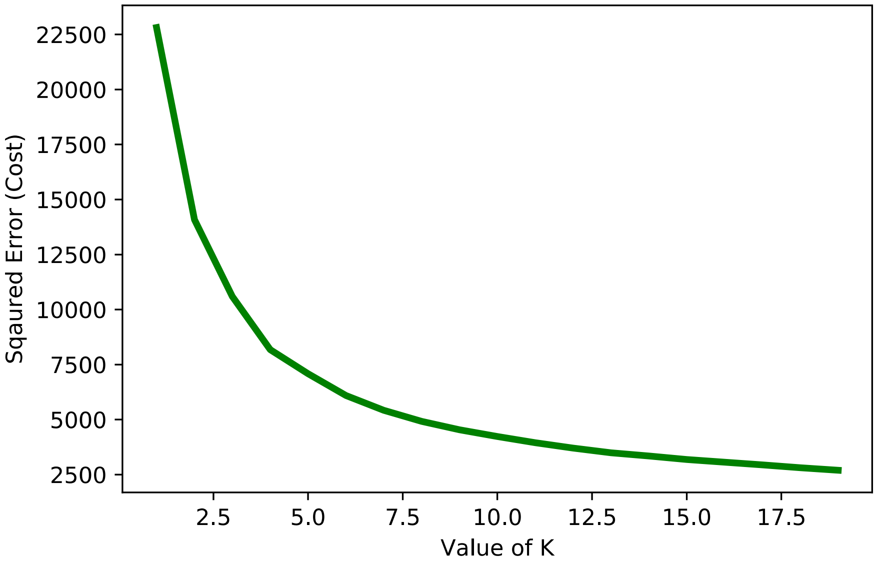

A range of methods were used to select the appropriate number of *k* clusters. An elbow plot of the cost function against *k* showed no clear 'elbow' but suggested a number of clusters from 4 to 7 (Figure A3). Calinski-Harabasz and Davies-Bouldin scores were calculated for *k* in the range 2-14.[7, 8] The former calculates the ratio of between-cluster sum of squared distances to the within-cluster sum of squared distances, with larger values indicating better dispersion. The latter calculates the average similarity of clusters, based on the average distance of each point in the cluster to the centroid, and the distance between cluster centroids, with smaller values indicating better separation. Table A3 displays both scores by value of *k*.

For peer review only - http://bmjopen.bmj.com/site/about/guidelines.xhtml

Table A3: Calinski-Harabasz and Davies-Bouldin scores for values of *k* clusters

k	Calinski-Harabasz	Davies-Bouldin
2	237582	1.09368
3	221031	1.15472
4	228535	0.99956
5	212661	1.07835
6	211027	0.99648
7	205075	1.00265
8	199811	1.03196
9	193744	1.02996
10	187409	1.07746
11	184038	1.04232
12	180593	1.01880
13	176750	1.02698
14	173582	1.02538

The Calinski-Harabasz score was highest for two clusters and declined gradually from four clusters, indicating better dispersion with a smaller number of clusters. The Davies-Bouldin score was lowest for six clusters, with similarly low scores for four, seven and eight clusters. The range from four to eight were selected as a focus for further description. Figures A4-A8 show the distributions of mean activity across the seven healthcare utilisation variables for each of the five models. Confusion matrices (Figures A9-A12) show the changes in assignment between consecutive increases in *k* clusters.

Utilisation within each cluster was assigned a descriptor based on total utilisation (low if mean attendances below average for all attendance types; medium if above average for at least one and fewer than four attendance types; and high if above average for four or more attendance types) and predominant attendance types accounting for utilisation.

In a 4-cluster model (Figures A4a and A4b), a low utilisation group and a moderate, GP predominant segment were formed. Two high utilisation segments were also formed, one utilising predominantly

For peer review only

GP and emergency care, and the other using all services. The 5-cluster model (Figures A5a and A5b) retained the first two clusters, but split the remaining higher utilisation clusters into a moderate; GP, outpatient and elective care segment, and a moderate utilisation; GP and emergency care group, retaining in addition a smaller high utilisation cluster. The 6-cluster (Figures A6a and A6b) model split the very low and the moderate; GP clusters into a new low utilisation; GP cluster.

The 7-cluster model (Figures A7a and A7b) split three segments (very low; low utilisation, GP; and moderate utilisation, GP and emergency care) into an additional low utilisation group characterised by lower than average GP use, but higher emergency care use. The 8-cluster model (Figures A8a and A8b) split the moderate utilisation GP, outpatient and elective care group into an additional moderate utilisation cluster characterised by lower than average GP use, but higher elective care use. Participants in other segments from the 7-cluster model were also reassigned on increasing to 8-clusters, suggesting inherently less stable clusters. The additional clusters produced by the 7- and 8-cluster models were decided to be less useful clinically as descriptors given the added complexity of the model, and the 6-cluster model was selected as the optimal number of segments.

Hierarchical clustering

Hierarchical agglomerative clustering using Ward's method was computationally feasible on sub-samples of up to 50,000 participants (13.2% of the total sample). In order to compare the similarity of cluster assignment between different random draws, a fixed sub-sample of 5,000 participants was randomly selected (R_F). The clustering algorithm was then iterated 10 times. On each iteration, a new sub-sample of 45,000 participants (R_V) was randomly selected, added to R_F to create a total sub-sample of 50,000 participants ($R_F + R_V$). Pair-wise comparisons of cluster membership for participants in R_F from each iteration compared to the preceding iteration were made using V-measure scores, the harmonic mean of homogeneity and completeness scores.[9] A range of V-measure scores from 0.765 – 0.918 were calculated, with a mean of 0.847, suggesting that cluster assignment was sensitive the sub-sample of data used.

For peer review only - http://bmjopen.bmj.com/site/about/guidelines.xhtml

Figure A4a: Mean number of attendances to outpatients, GP and A&E from the 4 cluster model

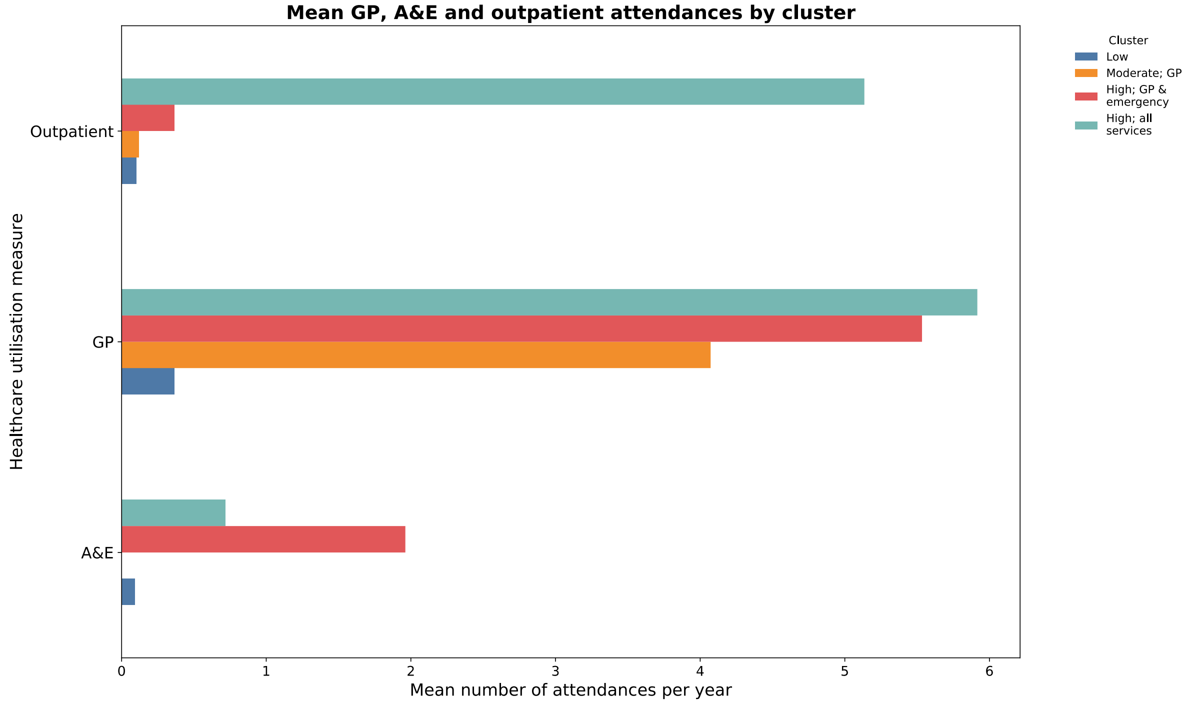

Figure A4b: Mean number of elective and emergency admission and yearly length of stay from the 4 cluster model

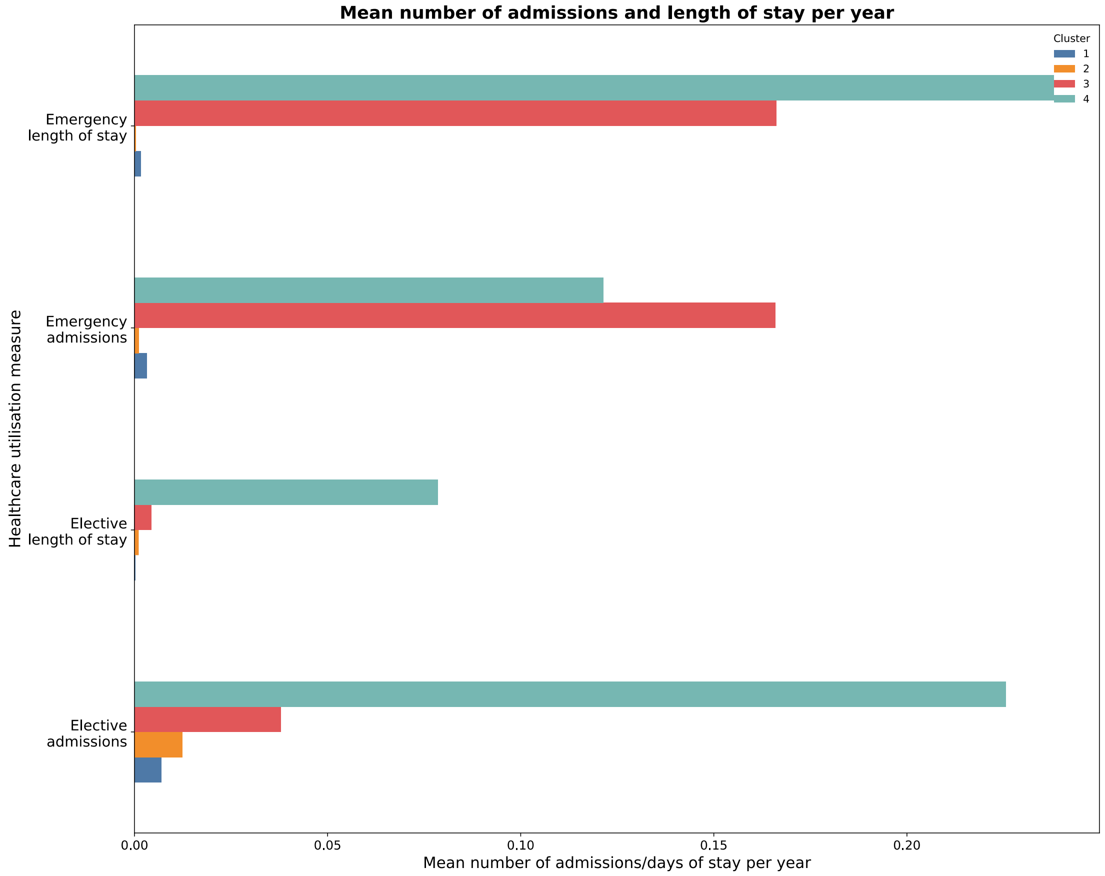

For peer review only - http://bmjopen.bmj.com/site/about/guidelines.xhtml

Figure A5a: Mean number of attendances to outpatients, GP and A&E from the 5 cluster model

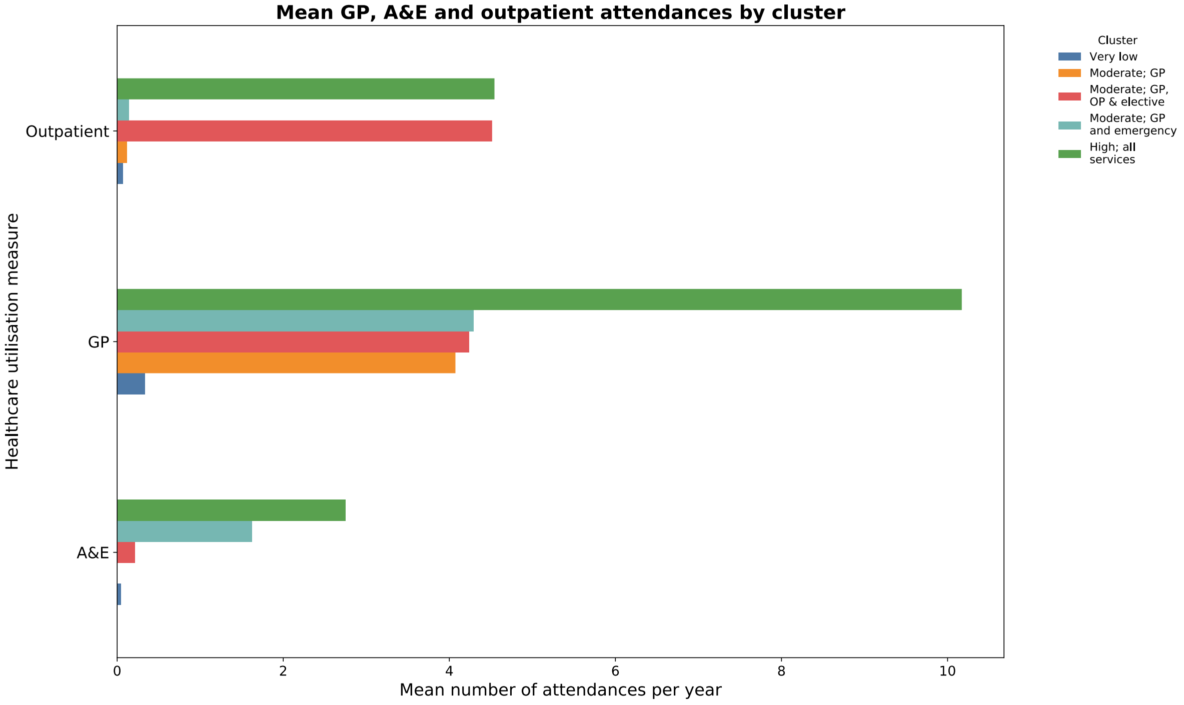

Figure A5b: Mean number of elective and emergency admission and yearly length of stay from the 5 cluster model

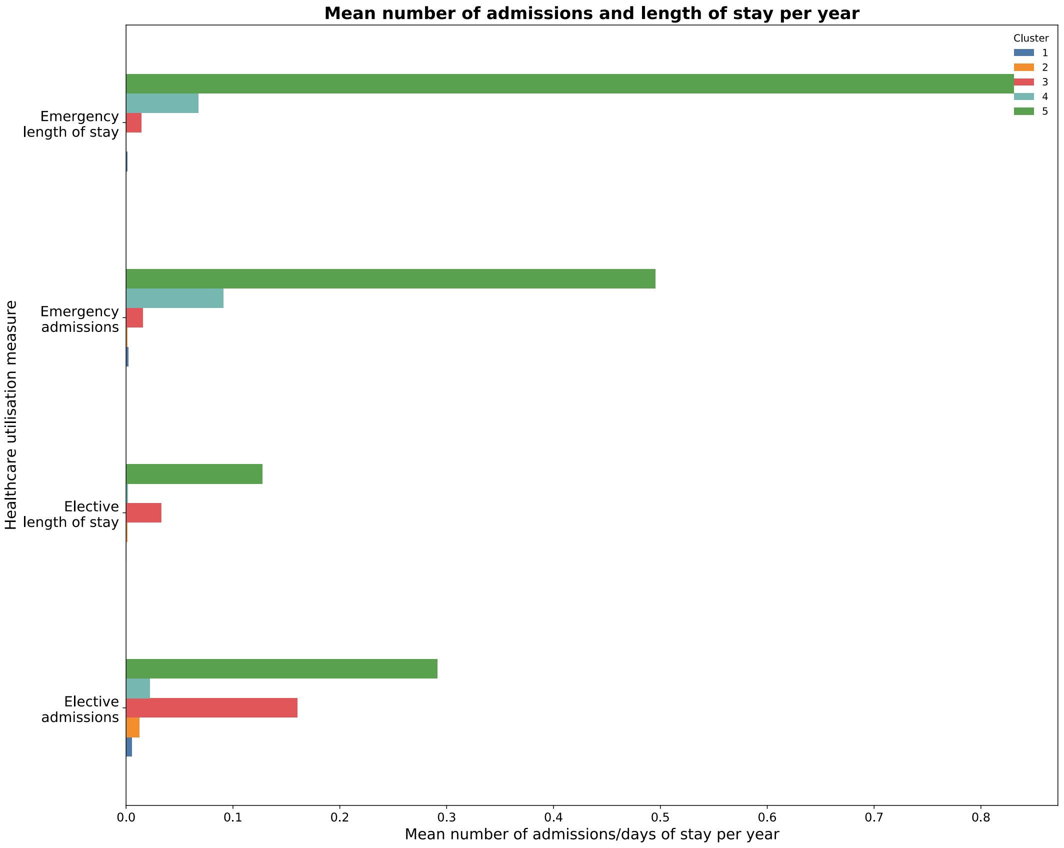

Figure A6a: Mean number of attendances to outpatients, GP and A&E from the 6 cluster model

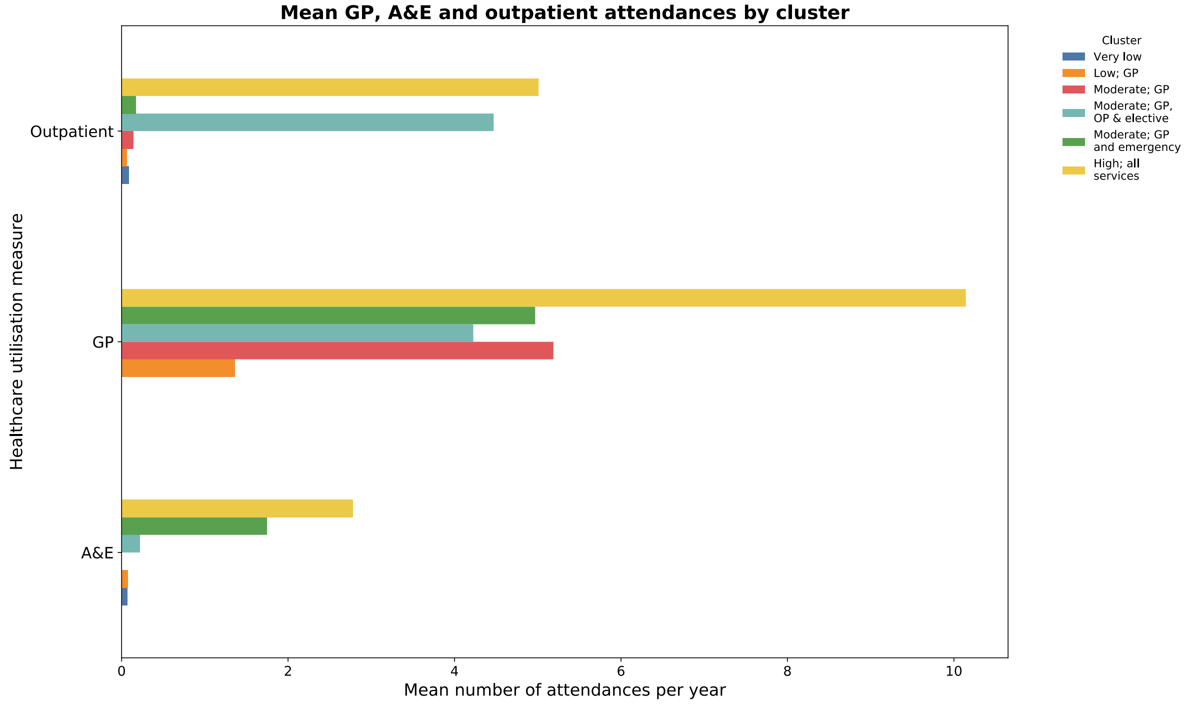

Figure A6b: Mean number of elective and emergency admission and yearly length of stay from the 6 cluster model

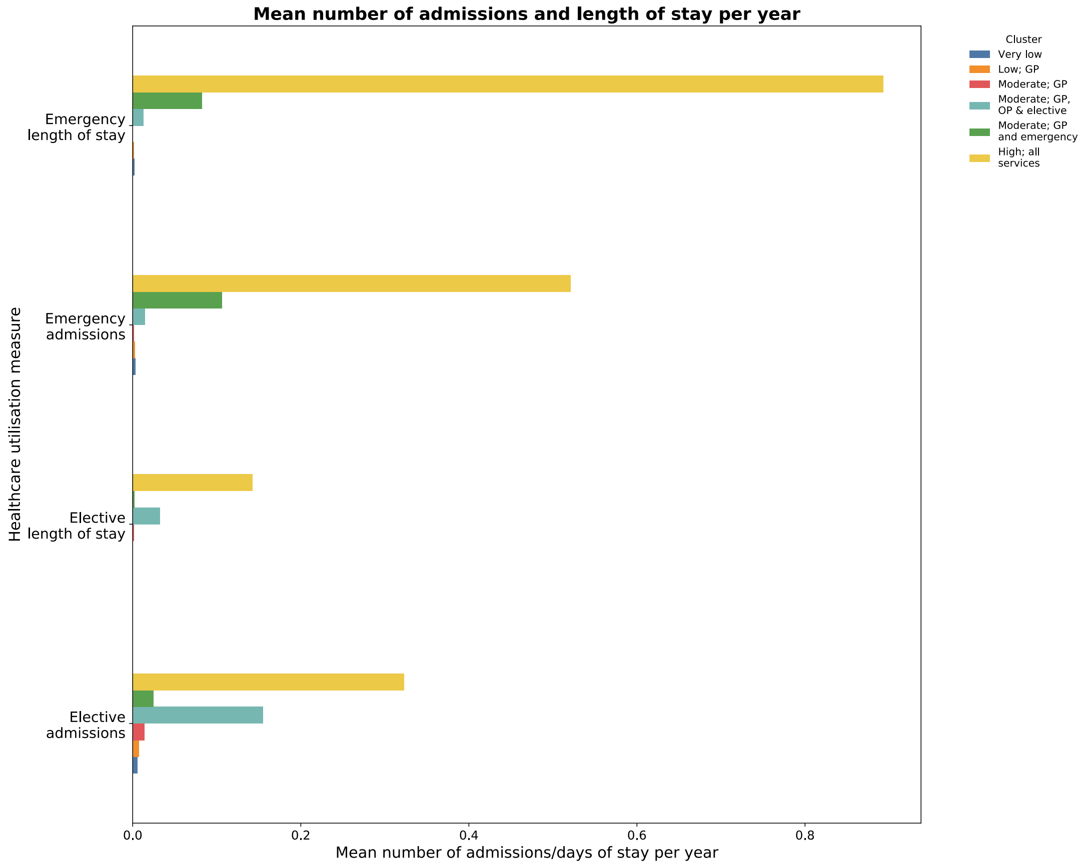

For peer review only - http://bmjopen.bmj.com/site/about/guidelines.xhtml

Figure A7a: Mean number of attendances to outpatients, GP and A&E from the 7 cluster model

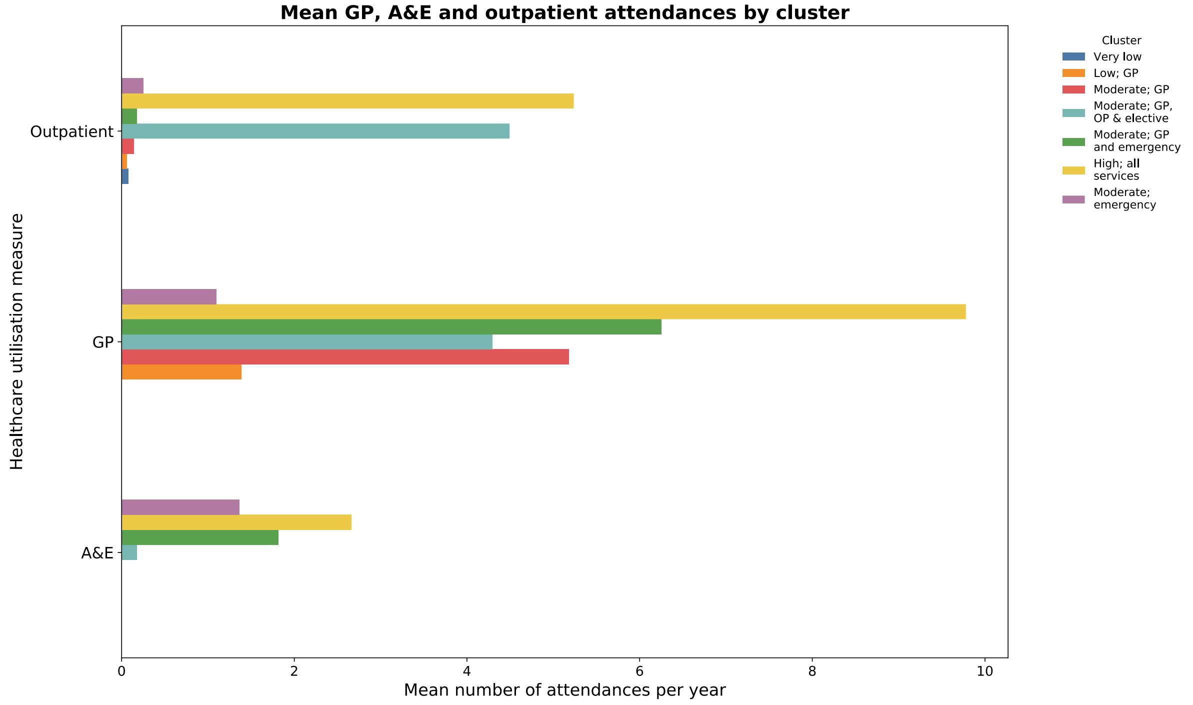

Figure A7b: Mean number of elective and emergency admission and yearly length of stay from the 7 cluster model

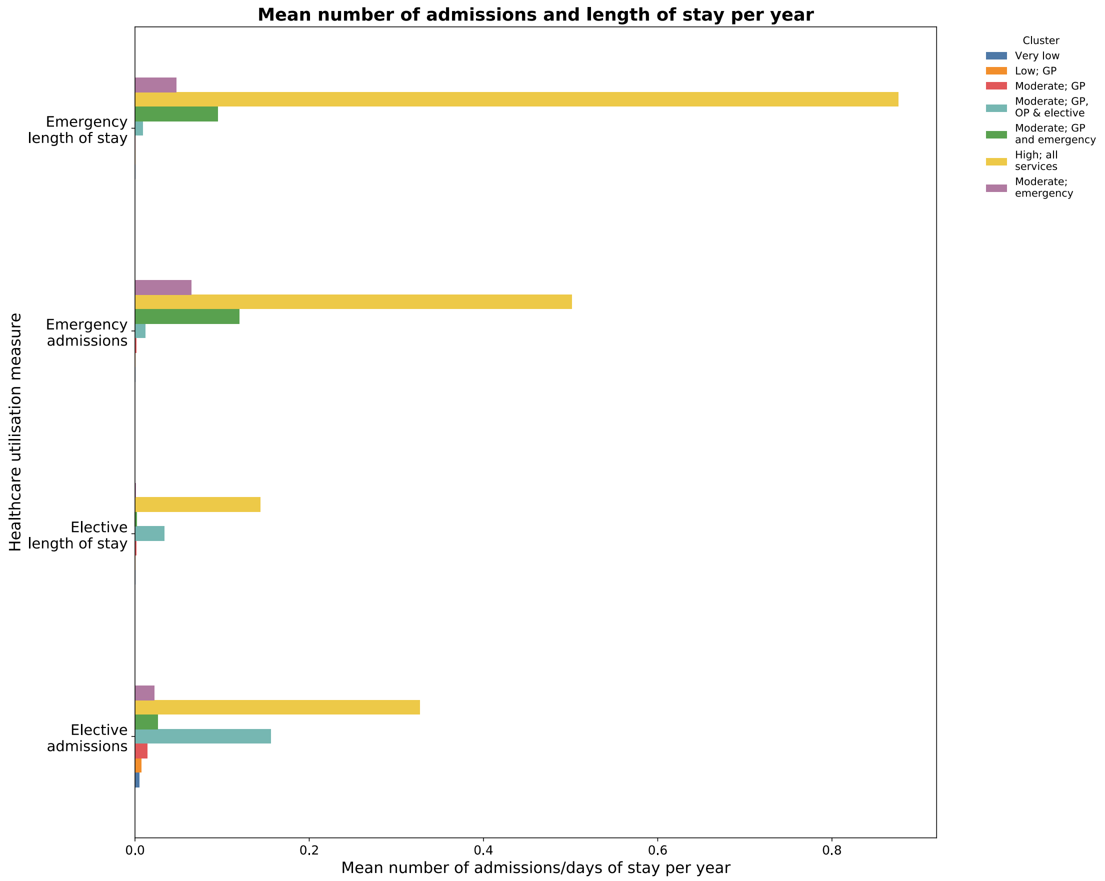

For peer review only - http://bmjopen.bmj.com/site/about/guidelines.xhtml

Figure A8a: Mean number of attendances to outpatients, GP and A&E from the 8 cluster model

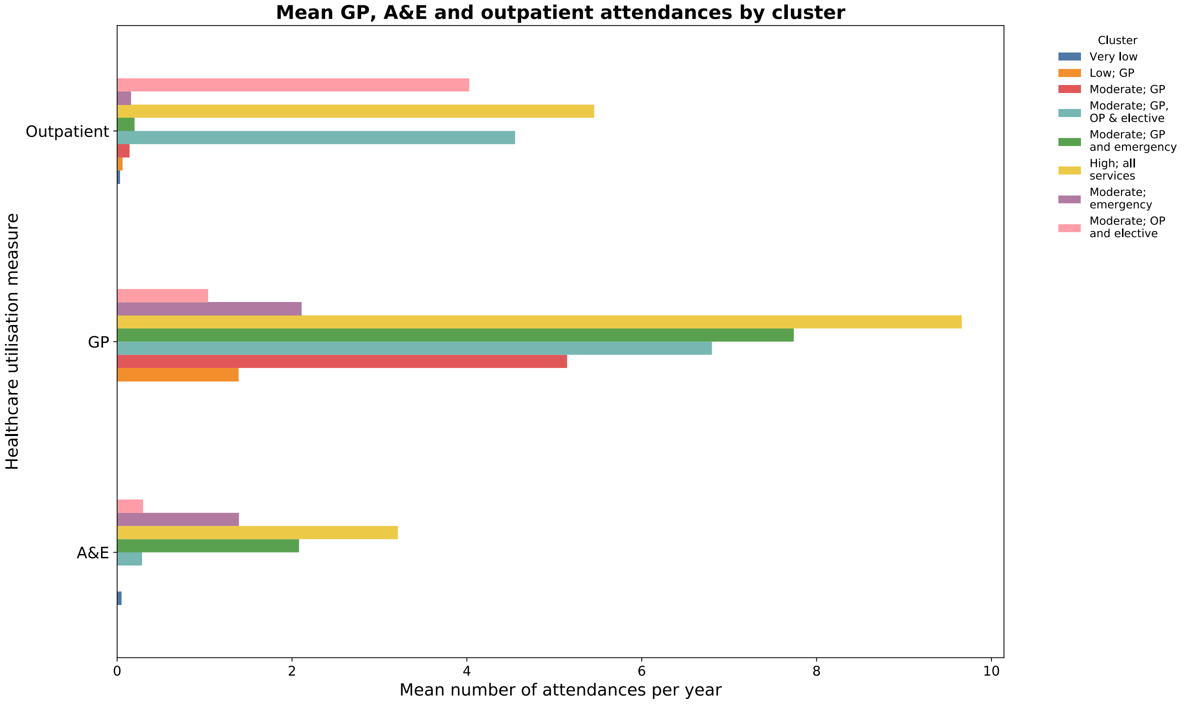

Figure A8b: Mean number of elective and emergency admission and yearly length of stay from the 8 cluster model

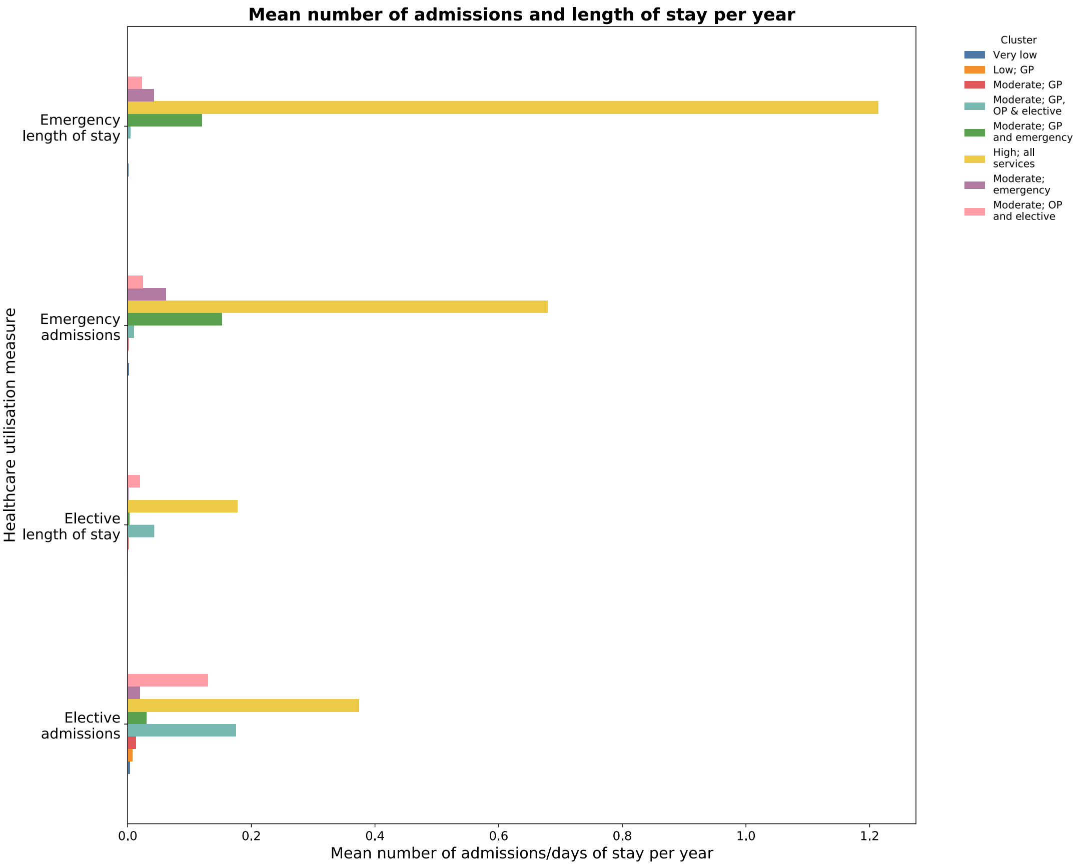

Figure A9: Confusion matrix for cluster assignment comparing 4 vs 5 clusters from k means

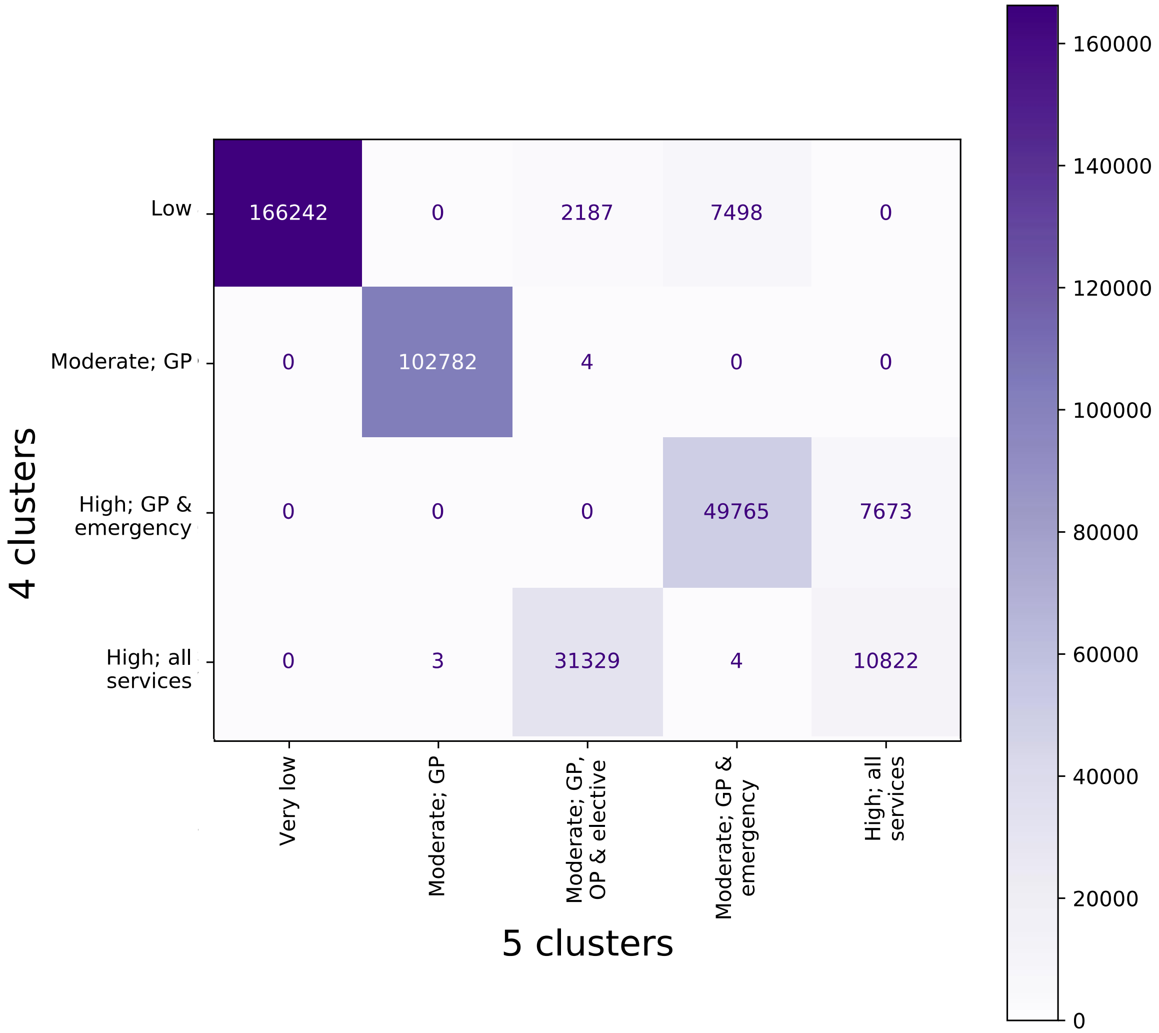

only

For peer review only - http://bmjopen.bmj.com/site/about/guidelines.xhtml

Figure A10: Confusion matrix for cluster assignment comparing 5 vs 6 clusters from k means

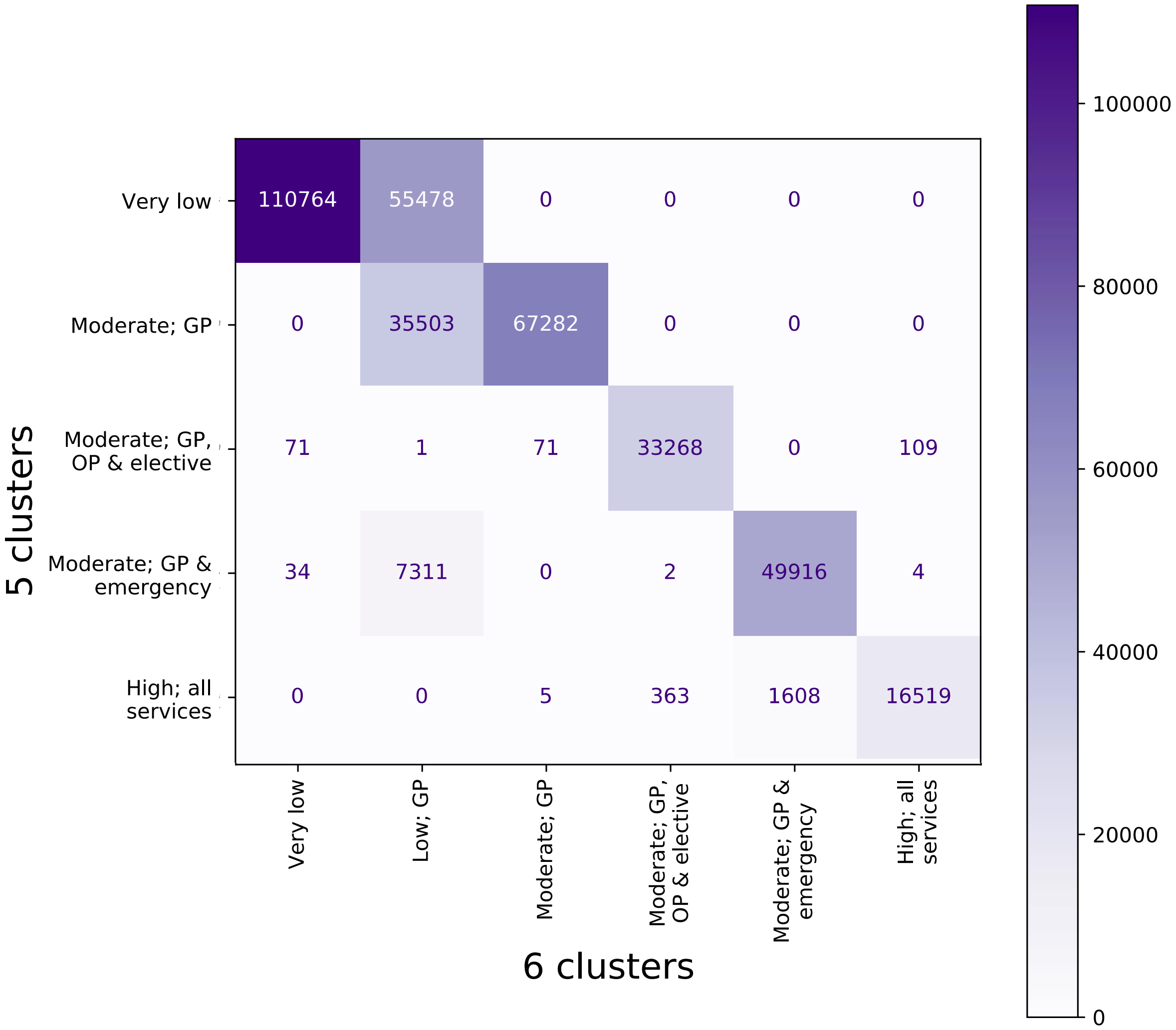

only

Figure A11: Confusion matrix for cluster assignment comparing 6 vs 7 clusters from k means

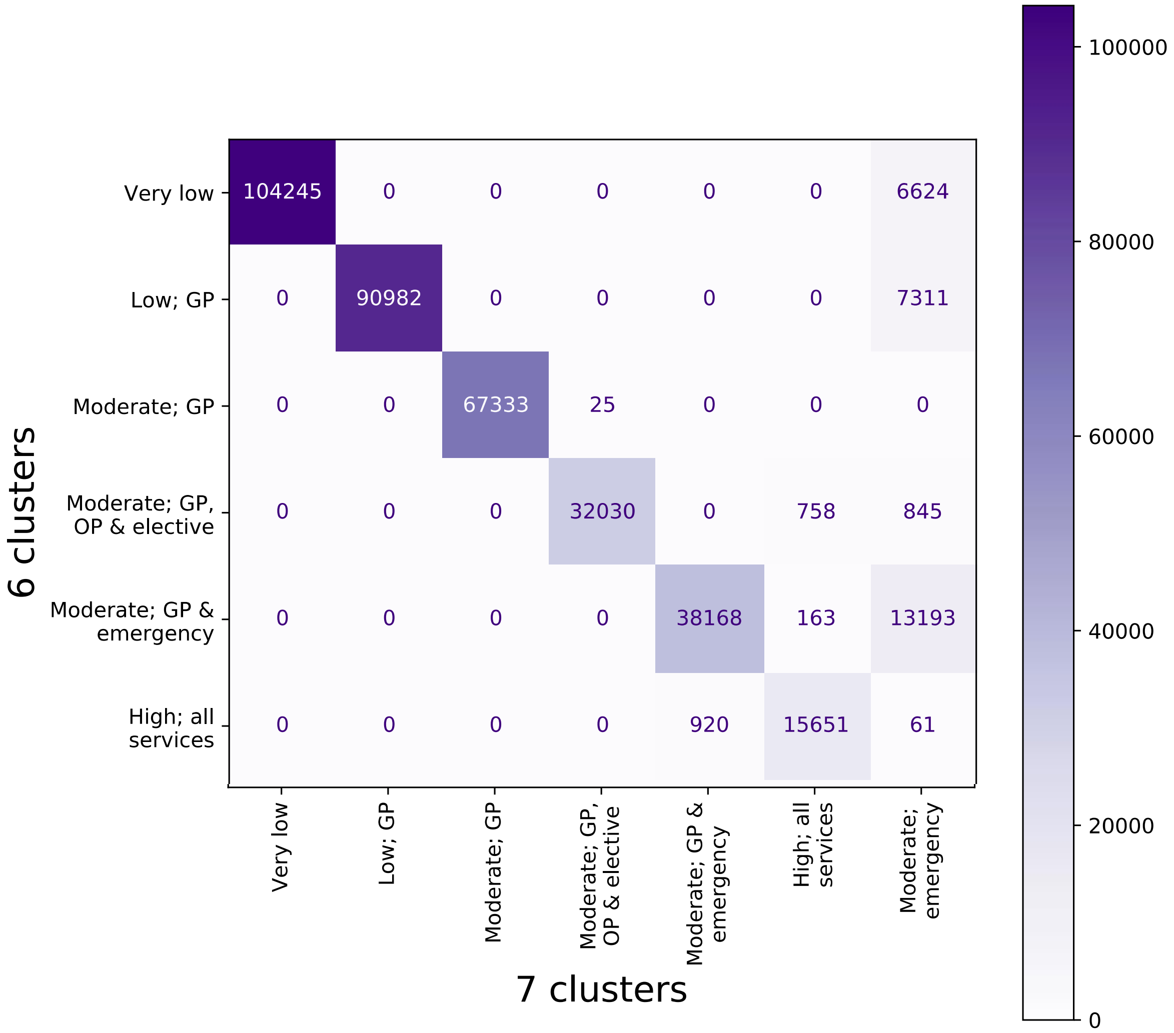

only

Figure A12: Confusion matrix for cluster assignment comparing 7 vs 8 clusters from k means

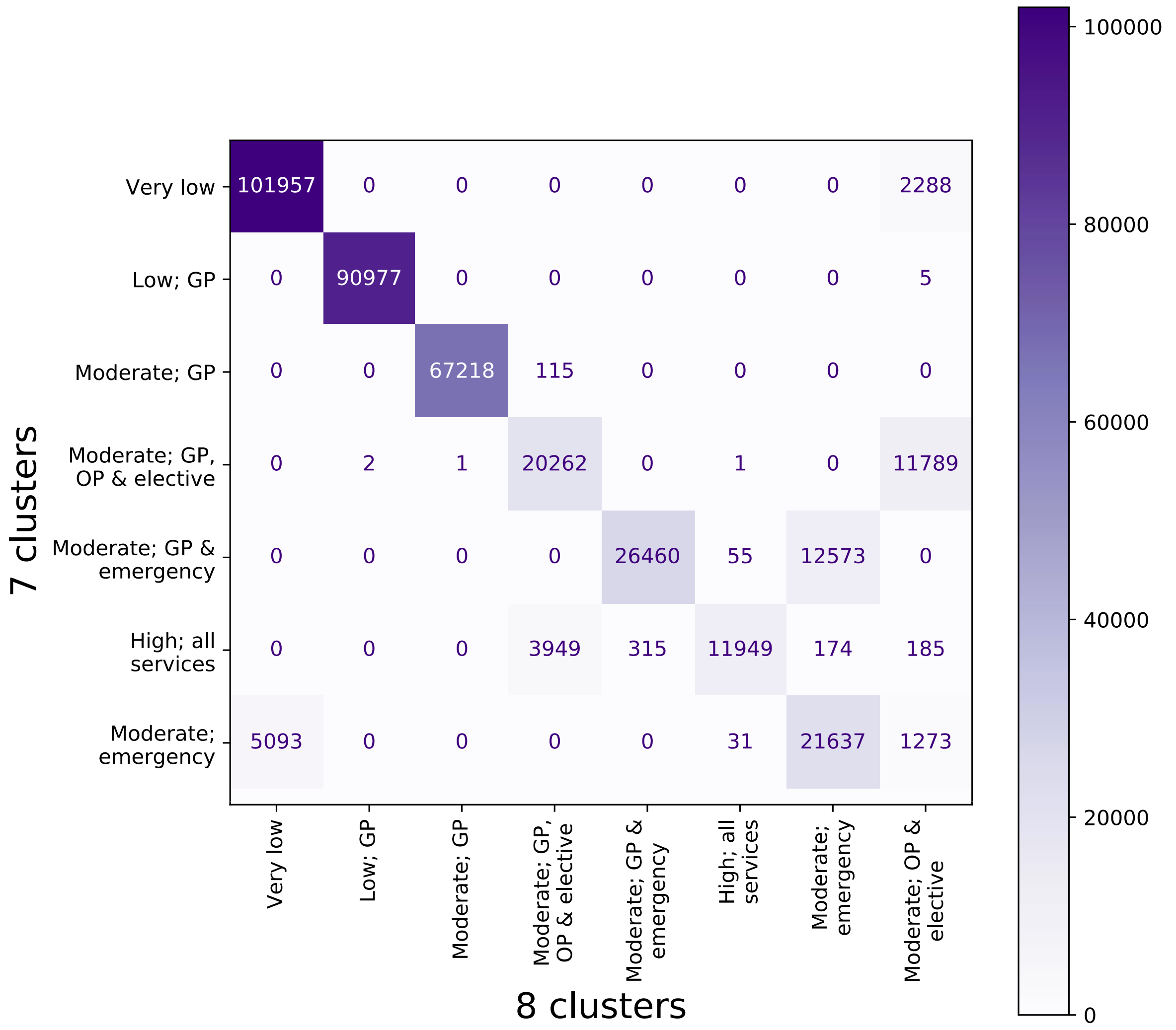

Figure A13: Confusion matrix for cluster assignment 6 clusters comparing models from k means and equivalent hierarchical clustering using Ward's method

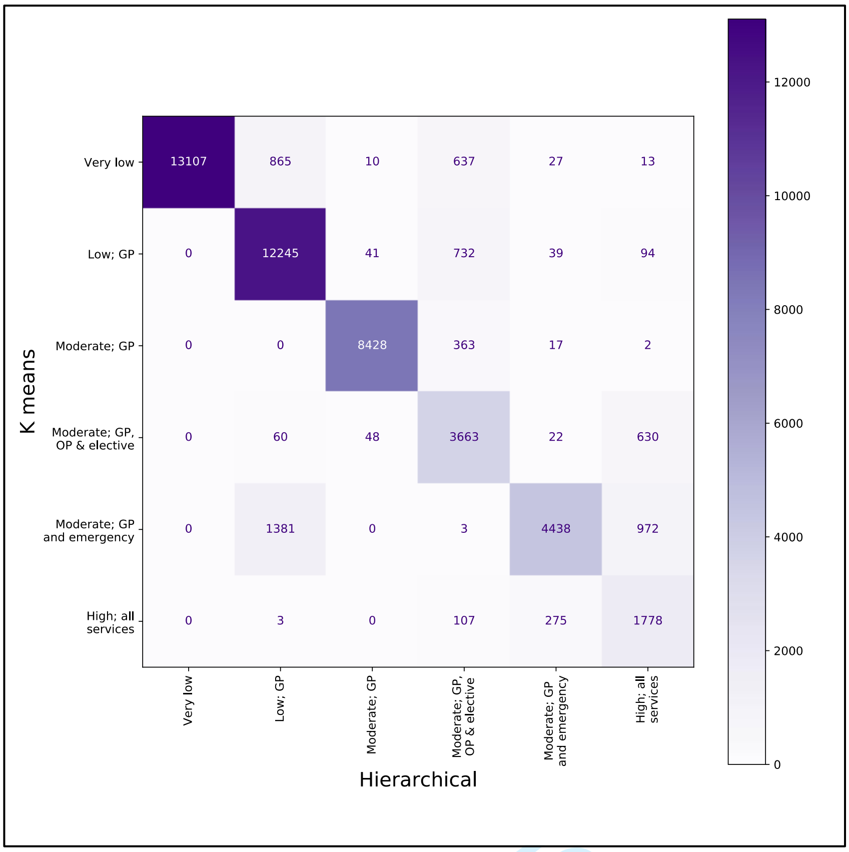

For peer review only

Table A4: Mean utilisation across each segment by utilisation variable from 6 cluster hierarchical model on subsample of 50,000 participants (green indicates lower than average utilisation, red indicates higher than average utilisation)

Segment	Size	Percentage	GP	Outpatient	A&E	Elective admissions	Elective length of stay	Emergency admissions	Emergency length of stay
I: No health service utilisation	12893	25.8%	0.00	0.00	0.00	0.00	0.00	0.00	0.00
II: Low; GP	13860	27.7%	1.70	0.00	0.00	0.00	0.00	0.00	0.00
III: Moderate; GP	5056	10.1%	6.27	0.01	0.00	0.02	0.00	0.00	0.00
IV: Moderate; GP, OP & elective	7497	15.0%	3.61	3.05	0.26	0.11	0.03	0.01	0.01
V: Moderate; GP & emergency care	7648	15.3%	4.20	0.03	1.71	0.02	0.00	0.11	0.08
VI: High; all services	3046	6.1%	8.61	4.15	2.22	0.24	0.07	0.38	0.67
**Total**	**50000**		**2.81**	**0.72**	**0.43**	**0.04**	**0.01**	**0.04**	**0.06**

For peer review only - http://bmjopen.bmj.com/site/about/guidelines.xhtml

Table A5: Median and interquartile range of presentations/length of stay over one year for each segment from 6 cluster k-means model (green indicates lower than average utilisation, red indicates higher than average utilisation)

k	Segment	Size	Percentage	GP	Outpatient	A&E	Elective admissions	Elective length of stay	Emergency admissions	Emergency length of stay
1	Very low	110869	29.3%	0 (0-0)	0 (0-0)	0 (0-0)	0 (0-0)	0 (0-0)	0 (0-0)	0 (0-0)
2	Low: GP	98293	26.0%	1 (1-2)	0 (0-0)	0 (0-0)	0 (0-0)	0 (0-0)	0 (0-0)	0 (0-0)
3	Moderate: GP	67358	17.8%	4 (3-6)	0 (0-0)	0 (0-0)	0 (0-0)	0 (0-0)	0 (0-0)	0 (0-0)
4	Moderate: GP, OP & elective	33633	8.9%	3 (2-6)	3 (2-5)	0 (0-0)	0 (0-0)	0 (0-0)	0 (0-0)	0 (0-0)
5	Moderate: GP & emergency	51524	13.6%	4 (2-6)	0 (0-0)	1 (1-2)	0 (0-0)	0 (0-0)	0 (0-0)	0 (0-0)
6	High: all services	16632	4.4%	8 (6-12)	3 (2-6)	2 (1-3)	0 (0-0)	0 (0-0)	0 (0-1)	0 (0-0)
	**Total**	**378309**		**2 (0-4)**	**0 (0-0)**	**0 (0-0)**	**0 (0-0)**	**0 (0-0)**	**0 (0-0)**	**0 (0-0)**

For peer review only - http://bmjopen.bmj.com/site/about/guidelines.xhtml

Table A6: Total healthcare associated costs for each segment from the 6-cluster k-means model

Segment	Size	Percentage	GP		Outpatient		A&E		Elective admissions		Emergency admissions		Total		Mean cost per capita (£)
			Total (£)	Percentage	Total (£)	Percentage	Total (£)	Percentage	Total (£)	Percentage	Total (£)	Percentage	Total (£)	Percentage	
I: Very low	110869	29.3%	0	0.0%	1677749	3.4%	846374	4.6%	700875	3.6%	315633	1.7%	3540631	2.5%	32
II: Low; GP	98293	26.0%	4415301	12.7%	1141363	2.3%	768704	4.2%	807133	4.1%	272191	1.4%	7404692	5.3%	75
III: Moderate; GP	67358	17.8%	11531157	33.3%	1862688	3.8%	0	0.0%	1095476	5.6%	71574	0.4%	14560895	10.4%	216
IV: Moderate; GP, OP & elective	33633	8.9%	4689465	13.5%	27200957	55.3%	823913	4.5%	7729103	39.5%	664487	3.5%	41107925	29.2%	1222
V: Moderate; GP & emergency care	51524	13.6%	8449386	24.4%	1691922	3.4%	10173249	55.4%	1406429	7.2%	5550515	29.6%	27271501	19.4%	529
VI: High; all services	16632	4.4%	5567562	16.1%	15639629	31.8%	5748710	31.3%	7832472	40.0%	11904047	63.4%	46692420	33.2%	2807
**Total**	**378309**		**34652871**		**49214308**		**18360950**		**19571488**		**18778447**		**140578064**		

For peer review only

Table A7: Population characteristics for each segment from 6-cluster k-means model

Participant characteristic		I: Very low		II: Low; GP		III: Moderate; GP		IV: Moderate; GP, OP & elective		V: Moderate; GP & emergency		VI: High; all services	
		Total	Proportion	Total	Proportion	Total	Proportion	Total	Proportion	Total	Proportion	Total	Proportion
**Gender**	Female	53,785	48.5%	49,476	50.3%	34,224	50.8%	15,485	46.0%	24,128	46.8%	7,310	44.0%
	Male	57,083	51.5%	48,816	49.7%	33,132	49.2%	18,147	54.0%	27,396	53.2%	9,321	56.0%
	Unknown	1	0.0%	1	0.0%	2	0.0%	1	0.0%	0	0.0%	1	0.0%
**Age (years) + female**	Mean (SD)	8.7 (4.0)		7.5 (4.3)		6.3 (4.8)		8.2 (4.6)		5.4 (4.7)		6.3 (5.1)	
	Median (IQR)	9 (6-12)		8 (4-11)		6 (2-10)		9 (4-12)		4 (1-9)		5 (1-11)	
	0	1,064	1.0%	2,304	2.3%	4,106	6.1%	864	2.6%	4,211	8.2%	1,222	7.3%
	1-2	2,997	2.7%	5,422	5.5%	5,958	8.8%	1,386	4.1%	4,708	9.1%	1,198	7.2%
	3-4	4,553	4.1%	6,395	6.5%	5,144	7.6%	1,730	5.1%	3,550	6.9%	873	5.2%
	5-10	25454	23.0%	21398	21.8%	10461	15.5%	5643	16.8%	7176	13.9%	2076	12.5%
	11-15	19,717	17.8%	13,957	14.2%	8,555	12.7%	5,862	17.4%	4,483	8.7%	1,941	11.7%
**Age (years) + male**	Mean (SD)	8.9 (4.0)		7.7 (4.3)		6.1 (4.6)		7.8 (4.6)		5.4 (4.6)		5.5 (4.8)	
	Median (IQR)	9 (6-12)		8 (4-11)		5 (2-10)		8 (4-12)		4 (1-9)		4 (1-10)	
	0	1,021	0.9%	2,036	2.1%	3,975	5.9%	1,084	3.2%	4,723	9.2%	1,694	10.2%
	1-2	3,038	2.7%	5,075	5.2%	5,698	8.5%	1,921	5.7%	5,398	10.5%	1,790	10.8%
	3-4	4,542	4.1%	5,976	6.1%	5,184	7.7%	2,203	6.6%	4,067	7.9%	1,342	8.1%
	5-10	25916	23.4%	21542	21.9%	11012	16.3%	6757	20.1%	8101	15.7%	2486	14.9%
	11-15	22,566	20.4%	14,187	14.4%	7,263	10.8%	6,182	18.4%	5,107	9.9%	2,009	12.1%
**Age (years)**	Mean (SD)	8.8 (4.0)		7.6 (4.3)		6.2 (4.7)		8.0 (4.6)		5.4 (4.6)		5.9 (4.9)	
	Median (IQR)	9 (6-12)		8 (4-11)		5 (2-10)		8 (4-12)		4 (1-9)		5 (1-10)	
	0	2,085	1.9%	4,340	4.4%	8,081	12.0%	1,948	5.8%	8,934	17.3%	2,916	17.5%
	1-2	6,035	5.4%	10,498	10.7%	11,656	17.3%	3,307	9.8%	10,106	19.6%	2,988	18.0%
	3-4	9,095	8.2%	12,371	12.6%	10,328	15.3%	3,933	11.7%	7,617	14.8%	2,215	13.3%

For peer review only

	5-10	51371	46.3%	42940	43.7%	21475	31.9%	12400	36.9%	15277	29.7%	4562	27.4%
	11-15	42,283	38.1%	28,144	28.6%	15,818	23.5%	12,045	35.8%	9,590	18.6%	3,951	23.8%
**Ethnicity**	Asian or Asian British	23,778	21.4%	24,124	24.5%	20,215	30.0%	7,749	23.0%	12,893	25.0%	3,945	23.7%
	White	27,950	25.2%	21,714	22.1%	12,481	18.5%	7,151	21.3%	10,726	20.8%	3,384	20.3%
	Mixed/multiple ethnic groups	10,425	9.4%	11,941	12.1%	7,060	10.5%	3,924	11.7%	6,376	12.4%	2,001	12.0%
	Other ethnic groups	10,566	9.5%	9,566	9.7%	6,889	10.2%	3,674	10.9%	5,581	10.8%	2,003	12.0%
	Black or Black British	7,749	7.0%	6,834	7.0%	4,225	6.3%	2,773	8.2%	3,138	6.1%	1,240	7.5%
	Unknown	30401	27.4%	24114	24.5%	16488	24.5%	8362	24.9%	12810	24.9%	4059	24.4%
**IMD quintile**	1 (most deprived)	15,282	13.8%	15,462	15.7%	10,670	15.8%	6,600	19.6%	9,101	17.7%	3,391	20.4%
	2	34,072	30.7%	31,174	31.7%	22,292	33.1%	10,792	32.1%	17,178	33.3%	5,666	34.1%
	3	29,050	26.2%	25,125	25.6%	17,163	25.5%	7,936	23.6%	13,028	25.3%	3,911	23.5%
	4	19,216	17.3%	15,457	15.7%	9,525	14.1%	4,453	13.2%	6,888	13.4%	1,906	11.5%
	5 (least deprived)	8,362	7.5%	7,054	7.2%	4,940	7.3%	2,385	7.1%	3,041	5.9%	931	5.6%
	Unknown	4,887	4.4%	4,021	4.1%	2,768	4.1%	1,467	4.4%	2,288	4.4%	827	5.0%
**Number of long-term conditions**	0	106,464	96.0%	91,248	92.8%	58,453	86.8%	27,083	80.5%	46,206	89.7%	12,457	74.9%
	One or more	4,015	3.6%	7,026	7.1%	8,890	13.2%	6,544	19.5%	5,308	10.3%	4,163	25.0%
	Missing	390	0.4%	19	0.0%	15	0.0%	6	0.0%	10	0.0%	12	0.1%
**Chronic condition***	Asthma	2,288	2.1%	4,469	4.5%	6,053	9.0%	3,128	9.3%	3,560	6.9%	2,161	13.0%
	Obesity	938	0.8%	1605	1.6%	1726	2.6%	993	3.0%	1139	2.2%	594	3.6%
	Mental health problem	276	0.2%	435	0.4%	631	0.9%	756	2.2%	398	0.8%	397	2.4%
	Learning Disability	84	0.1%	148	0.2%	394	0.6%	527	1.6%	133	0.3%	321	1.9%
	Chronic heart disease	245	0.2%	279	0.3%	237	0.4%	344	1.0%	186	0.4%	232	1.4%
	Chronic neurological disease & epilepsy	45	0.0%	93	0.1%	135	0.2%	508	1.5%	67	0.1%	461	2.8%
	Hypothyroidism	108	0.1%	125	0.1%	229	0.3%	396	1.2%	76	0.1%	154	0.9%

For peer review only

Cancer & immunosuppression	124	0.1%	152	0.2%	146	0.2%	315	0.9%	95	0.2%	229	1.4%
Diabetes	18	0.0%	16	0.0%	20	0.0%	440	1.3%	14	0.0%	267	1.6%
**Total participants**	**110,869**	**29.3%**	**98,293**	**26.0%**	**67,358**	**17.8%**	**33,633**	**8.9%**	**51,524**	**13.6%**	**16,632**	**4.4%**

* For those with ≥500 with condition. Proportion of those with non-missing data on LTC

For peer review only

For peer review only - http://bmjopen.bmj.com/site/about/guidelines.xhtml

References

1. Bottle A, Cohen C, Lucas A, Saravanakumar K, Ul-Haq Z, Smith W, et al. How an electronic health record became a real-world research resource: comparison between London's Whole Systems Integrated Care database and the Clinical Practice Research Datalink. BMC Med Inform Decis Mak. 2020;20:71.

2. Aitchison K, McGeown H, Holden B, Watson M, Klaber RE, Hargreaves D. Population child health: understanding and addressing complex health needs. Arch Dis Child. 2020. doi:10.1136/archdischild-2019-317373.

3. NHS England, British Medical Association. Seasonal influenza vaccination programme clinical used for payment. 2018. https://www.google.com/url?sa=t&rct=j&q=&esrc=s&source=web&cd=&cad=rja&uact=8&ved=2ahUKEwjBybDg5PXtAhUiVBUIHR34BxcQFjAJegQIAxAC&url=https%3A%2F%2Fwww.england.nhs.uk%2Fwp-content%2Fuploads%2F2019%2F07%2Fqsr-sfl-2019-20-codes-list.xlsm&usg=AOvVaw1Mt9mxgXhT_. Accessed 30 Dec 2020.

4. Curtis LA, Burns A. Unit Costs of Health and Social Care 2019. PSSRU, University of Kent; 2019. https://kar.kent.ac.uk/79286/.

5. NHS Improvement. Developing the national tariff. 2020.

6. NHS England. National Cost Collection: National Schedule of NHS costs - Year 2018-19 - NHS trust and NHS foundation trusts. National Cost Collection for the NHS. 2019.

7. Davies DL, Bouldin DW. A Cluster Separation Measure. IEEE Trans Pattern Anal Mach Intell. 1979;PAMI-1:224–7.

8. Caliński T, Harabasz J. A dendrite method for cluster analysis. Commun Stat. 1974;3:1–27. doi:10.1080/03610927408827101.

9. Rosenberg A, Hirschberg J. V-Measure: A conditional entropy-based external cluster evaluation measure. EMNLP-CoNLL 2007 - Proc 2007 Jt Conf Empir Methods Nat Lang Process Comput Nat Lang Learn. 2007; June:410–20.

STROBE Statement—Checklist of items that should be included in reports of ***cohort studies***

	Item No	Recommendation	Page No
**Title and abstract**	1	(*a*) Indicate the study's design with a commonly used term in the title or the abstract<br>(*b*) Provide in the abstract an informative and balanced summary of what was done and what was found	2
**Introduction**			
Background/rationale	2	Explain the scientific background and rationale for the investigation being reported	4-5
Objectives	3	State specific objectives, including any prespecified hypotheses	5
**Methods**			
Study design	4	Present key elements of study design early in the paper	5-6
Setting	5	Describe the setting, locations, and relevant dates, including periods of recruitment, exposure, follow-up, and data collection	5
Participants	6	(*a*) Give the eligibility criteria, and the sources and methods of selection of participants. Describe methods of follow-up<br>(*b*) For matched studies, give matching criteria and number of exposed and unexposed	5-6
Variables	7	Clearly define all outcomes, exposures, predictors, potential confounders, and effect modifiers. Give diagnostic criteria, if applicable	6-8
Data sources/ measurement	8*	For each variable of interest, give sources of data and details of methods of assessment (measurement). Describe comparability of assessment methods if there is more than one group	6-8
Bias	9	Describe any efforts to address potential sources of bias	8
Study size	10	Explain how the study size was arrived at	5-6
Quantitative variables	11	Explain how quantitative variables were handled in the analyses. If applicable, describe which groupings were chosen and why	5-7
Statistical methods	12	(*a*) Describe all statistical methods, including those used to control for confounding<br>(*b*) Describe any methods used to examine subgroups and interactions<br>(*c*) Explain how missing data were addressed<br>(*d*) If applicable, explain how loss to follow-up was addressed<br>(*e*) Describe any sensitivity analyses	7-8
**Results**			
Participants	13*	(a) Report numbers of individuals at each stage of study—eg numbers potentially eligible, examined for eligibility, confirmed eligible, included in the study, completing follow-up, and analysed<br>(b) Give reasons for non-participation at each stage<br>(c) Consider use of a flow diagram	8
Descriptive data	14*	(a) Give characteristics of study participants (eg demographic, clinical, social) and information on exposures and potential confounders<br>(b) Indicate number of participants with missing data for each variable of interest<br>(c) Summarise follow-up time (eg, average and total amount)	9
Outcome data	15*	Report numbers of outcome events or summary measures over time	9

For peer review only

Main results	16	(*a*) Give unadjusted estimates and, if applicable, confounder-adjusted estimates and their precision (eg, 95% confidence interval). Make clear which confounders were adjusted for and why they were included<br>(*b*) Report category boundaries when continuous variables were categorized<br>(*c*) If relevant, consider translating estimates of relative risk into absolute risk for a meaningful time period	10-14
Other analyses	17	Report other analyses done—eg analyses of subgroups and interactions, and sensitivity analyses	14
**Discussion**			
Key results	18	Summarise key results with reference to study objectives	14-15
Limitations	19	Discuss limitations of the study, taking into account sources of potential bias or imprecision. Discuss both direction and magnitude of any potential bias	17-19
Interpretation	20	Give a cautious overall interpretation of results considering objectives, limitations, multiplicity of analyses, results from similar studies, and other relevant evidence	15-17
Generalisability	21	Discuss the generalisability (external validity) of the study results	17-18
**Other information**			
Funding	22	Give the source of funding and the role of the funders for the present study and, if applicable, for the original study on which the present article is based	19

*Give information separately for exposed and unexposed groups.

Note: An Explanation and Elaboration article discusses each checklist item and gives methodological background and published examples of transparent reporting. The STROBE checklist is best used in conjunction with this article (freely available on the Web sites of PLoS Medicine at http://www.plosmedicine.org/, Annals of Internal Medicine at http://www.annals.org/, and Epidemiology at http://www.epidem.com/). Information on the STROBE Initiative is available at http://www.strobe-statement.org.

For peer review only

BMJ Open

Patterns of healthcare utilisation in children and young people: a retrospective cohort study using routinely collected healthcare data in North West London

Journal:	*BMJ Open*
Manuscript ID	bmjopen-2021-050847.R1
Article Type:	Original research
Date Submitted by the Author:	15-Nov-2021
Complete List of Authors:	Beaney, Thomas; Imperial College London, Department of Primary Care and Public Health; Imperial College London, National Institute for Health Research Applied Research Collaboration North West London<br>Clarke, Jonathan; Imperial College of Science Technology and Medicine, Department of Mathematics and Centre for Mathematics of Precision Healthcare<br>Woodcock, Thomas; Imperial College London, Department of Primary Care and Public Health; Imperial College London, National Institute for Health Research Applied Research Collaboration North West London<br>McCarthy, Rachel; North West London Collaboration of Clinical Commissioning Groups<br>Saravanakumar, Kavitha; orth West London Collaboration of Clinical Commissioning Groups<br>Barahona, Mauricio; Imperial College London, Centre for Mathematics of Precision Healthcare; Imperial College London, Department of Mathematics<br>Blair, Mitch; Imperial College London, Department of Primary Care and Public Health; Imperial College London, National Institute for Health Research Applied Research Collaboration North West London<br>Hargreaves, Dougal; Imperial College London, Primary Care and Public Health; Imperial College London, National Institute for Health Research Applied Research Collaboration North West London
**Primary Subject Heading**:	Public health
Secondary Subject Heading:	Paediatrics, Public health
Keywords:	Health informatics < BIOTECHNOLOGY & BIOINFORMATICS, HEALTH SERVICES ADMINISTRATION & MANAGEMENT, PAEDIATRICS, PUBLIC HEALTH, STATISTICS & RESEARCH METHODS

SCHOLARONE™
Manuscripts

I, the Submitting Author has the right to grant and does grant on behalf of all authors of the Work (as defined in the below author licence), an exclusive licence and/or a non-exclusive licence for contributions from authors who are: i) UK Crown employees; ii) where BMJ has agreed a CC-BY licence shall apply, and/or iii) in accordance with the terms applicable for US Federal Government officers or employees acting as part of their official duties; on a worldwide, perpetual, irrevocable, royalty-free basis to BMJ Publishing Group Ltd ("BMJ") its licensees and where the relevant Journal is co-owned by BMJ to the co-owners of the Journal, to publish the Work in this journal and any other BMJ products and to exploit all rights, as set out in our licence.

The Submitting Author accepts and understands that any supply made under these terms is made by BMJ to the Submitting Author unless you are acting as an employee on behalf of your employer or a postgraduate student of an affiliated institution which is paying any applicable article publishing charge ("APC") for Open Access articles. Where the Submitting Author wishes to make the Work available on an Open Access basis (and intends to pay the relevant APC), the terms of reuse of such Open Access shall be governed by a Creative Commons licence – details of these licences and which Creative Commons licence will apply to this Work are set out in our licence referred to above.

Other than as permitted in any relevant BMJ Author's Self Archiving Policies, I confirm this Work has not been accepted for publication elsewhere, is not being considered for publication elsewhere and does not duplicate material already published. I confirm all authors consent to publication of this Work and authorise the granting of this licence.

For peer review only

Patterns of healthcare utilisation in children and young people: a retrospective cohort study using routinely collected healthcare data in Northwest London

Thomas Beaney[1,2]

Jonathan M Clarke[3]

Thomas Woodcock[1,2]

Rachel McCarthy[4]

Kavitha Saravanakumar[4]

Mauricio Barahona[3]

Mitch Blair[1,2]

Dougal Hargreaves[1,2]

1. Department of Primary Care and Public Health, Imperial College London, London, W6 8RP, UK.
2. National Institute for Health Research Applied Research Collaboration Northwest London, Imperial College, London, W6 8RP, UK.
3. Department of Mathematics and Centre for Mathematics of Precision Healthcare, Imperial College London, London, SW7 2AZ, UK.
4. North West London Collaboration of Clinical Commissioning Groups, London, NW1 5JD, UK.

Corresponding author:

Thomas Beaney

Department of Primary Care and Public Health, Imperial College London, London, W6 8RP, UK.

Email: Thomas.beaney@imperial.ac.uk

For peer review only

Abstract (word count 300)

Objectives

With a growing role for health services in managing population health, there is a need for early identification of populations with high need. Segmentation approaches partition the population based on demographics, long-term conditions (LTCs) or healthcare utilisation but have mostly been applied to adults. Our study uses segmentation methods to distinguish patterns of healthcare utilisation in children and young people (CYP) and to explore predictors of segment membership.

Design

Retrospective cohort study.

Setting

Routinely collected primary and secondary healthcare data in Northwest London from the Discover database.

Participants

378,309 CYP aged 0-15 years registered to a general practice in Northwest London with one full year of follow-up.

Primary and secondary outcome measures

Assignment of each participant to a segment defined by seven healthcare variables representing primary and secondary care attendances, and description of utilisation patterns by segment. Predictors of segment membership described by age, sex, ethnicity, deprivation and LTCs.

Results

Participants were grouped into six segments based on healthcare utilisation. Three segments predominantly used primary care; two moderate utilisation segments differed in use of emergency or elective care, and a high utilisation segment, representing 16,632 (4.4%) children accounted for the highest mean presentations across all service types. The two smallest segments, representing 13.3% of the population, accounted for 62.5% of total costs. Younger age, residence in areas of

For peer review only

higher deprivation, and presence of one or more LTCs were associated with membership of higher utilisation segments, but 75.0% of those in the highest utilisation segment had no LTC.

Conclusions

This article identifies six segments of healthcare utilisation in CYP and predictors of segment membership. Demographics and LTCs may not explain utilisation patterns as strongly as in adults which may limit the use of routine data in predicting utilisation and suggests children have less well-defined trajectories of service use than adults.

Strengths and limitations of this study

- Segmentation approaches have been widely used in the adult population for the purposes of population health management, but are less widely used in children.
- This study uses routinely collected primary and secondary care data, including all eligible patients registered to general practices in Northwest London region.
- Using a data-driven approach with k-means clustering, we defined six distinct segments of the child population in Northwest London based on healthcare utilisation patterns.
- Age, sex, ethnicity, deprivation and number of long-term conditions were strong predictors of utilisation segment, but three-quarters of children in the highest utilisation segment had no long-term condition.
- Demographics and co-morbidities may not capture the variation in healthcare utilisation to the same extent as in adults and further research is needed to identify whether additional factors in the electronic health record can predict utilisation in children.

For peer review only

Introduction

There is growing recognition, both internationally and within the UK, of the role of the health service in managing population health.[1–3] Population health has become a core responsibility of NHS organisations and encompasses the activities that can be taken at a system level to improve physical and mental health and wellbeing, and reduce health inequalities for a whole population, rather than focussing solely on provision of services for individual patients.[4] Critical to population health activities is the ability to identify and understand the needs of those for whom early intervention may improve outcomes, and thus reduce the need for more costly hospital-based care. The life course approach to prevention suggests that the long-term benefits of early intervention are greatest in children and young people (CYP), particularly in the first 1,000 days of life, highlighting the need for population health approaches tailored towards younger people.[5]

Segmentation methods are an approach to categorising individuals with similar characteristics with the aim of identifying common care needs and designing services tailored to those in each segment to improve health outcomes and optimise healthcare utilisation.[6, 7]. A variety of approaches to population segmentation in health settings have been described, based mostly on demographic and medical factors and including both expert-driven and data-driven designs, or a mix of the two.[8, 9] Segmenting on healthcare utilisation is an alternative, and included in some approaches, but only one previous segmentation method was based solely on utilisation.[10] Existing work on segmentation methods have applied to adults, who may represent a very different population to that of CYP in terms of chronic disease burden and patterns of healthcare utilisation, indicating a need for research focussed on the CYP population.

In the adult population, health care utilisation and expenditure are closely associated with increasing age and co-morbidities: in a recent study of healthcare costs in England, adults accounting for the top 5% of costs were significantly older and 85.5% had at least one long-term condition (LTC).[11] In

For peer review only

contrast, in the CYP population, a higher proportion of under-5s accounted for the top 5% of costs (compared with those aged 5-9 and 10-14 years), with only half of those in the high-cost group having no defined LTC.[12] These findings suggest that approaches to segmentation using age and LTC will only partially account for the utilisation and costs associated with care in CYP, and that other factors, including those available in electronic health records (EHRs) such as sex, ethnicity and deprivation may also play a role.

Exploration of the link between modifiable and non-modifiable risk factors in relation to children's use of health services is key to developing our understanding of the utility and limitations of segmentations approaches, and the extent to which health need can be identified and intervened upon pre-emptively. The aim of this article is to use a data-driven method to characterise different patterns of healthcare utilisation over the course of a year in the CYP population for Northwest London, using routinely available healthcare data. We then describe the population characteristics of the resulting segments, by age, sex, ethnicity, LTC burden and deprivation in order to identify population groups with different patterns of healthcare utilisation

Methods

Data source and cohort design

Data for this study used the Discover platform to access the Whole Systems Integrated Care (WSIC) database. WSIC holds the records of 2.4 million patients registered to general practices (GPs) within Northwest London, representing 95% of the registered population and comprising both primary and secondary care data.[13] Primary care data are extracted directly from the EHR, and are linked to secondary care data from the Secondary Uses Service (SUS) dataset of accident and emergency (A&E) attendances, inpatient admissions (including both elective and emergency admissions) and outpatient attendances covering all hospital providers in England.

For peer review only

A retrospective cohort was constructed of all CYP aged 0-15 years who were registered as of 01/03/2019 with a full year (365 days) of follow-up until 29/02/2020. These dates were chosen to provide contemporaneous data while minimising the overlap with the COVID-19 pandemic, due to expected changes in usual healthcare utilisation patterns. For children who had died over the study time period, 365 days of follow-up were included prior to the date of death to ensure a comparable time period, allowing an earliest study start date of 01/03/2018.

Healthcare utilisation, cost and LTCs

Seven healthcare utilisation variables were chosen, for which attendance is robustly recorded in the clinical record and summed across the year

1. GP attendances (including all consultation and staff types)
2. A&E attendances
3. Outpatient attendances
4. Elective hospital admissions
5. Total length of stay from elective admissions (days)
6. Emergency hospital admissions
7. Total length of stay from emergency admissions (days)

Estimated healthcare costs for primary care were derived from the Unit Costs of Health and Social Care 2019, assuming average consultation times and including direct care staff costs.[14] Primary care prescription costs were not included. Secondary care costs are included directly in Discover based on the total National Tariff Payment System cost associated with each attendance or admission.[15] Where these were missing, costs were estimated based on the Unit Costs of Health and Social Care 2019 (for paediatric outpatient attendances) or the National Schedule of NHS Costs (A&E attendances and emergency/elective admissions).[16] Co-morbidities were selected from among 41 LTCs defined in Discover. A sub-set of seventeen of these chronic conditions identified in

For peer review only

previous literature as relevant to children were included [17] (online supplemental file, table S1), supplemented by additional SNOMED code sets for relevant conditions not included in Discover (online supplemental file, table S2) [18] identified from the entirety of the patient primary care record. Secondary care diagnostic codes were not included in order not to bias number of conditions in favour of those presenting to secondary care, although they may be coded in the primary care record. Further details of the healthcare utilisation variables and LTCs are given in the online supplemental file, p2-4.

Cluster analysis

Two methods were considered for cluster analysis, which have been widely used in the segmentation literature: k-means and hierarchical agglomerative clustering, following the approach used by Vuik *et al* (2016).[9, 10] Given our population approach, use of the entire dataset was preferrable. Hierarchical methods are computationally intensive and may not run on datasets with several hundreds of thousands of samples and so k-means was chosen as the primary clustering method with a sensitivity compared to Ward's hierarchical agglomerative clustering on a sub-set of data.[10] Distributions of each utilisation measure were examined using histograms, were log-transformed to reduce skew and rescaled using min-max scaling. K-means clustering was performed, partitioning the cohort using Euclidean distance on the seven transformed and scaled utilisation variables. The algorithm was iterated 10,000 times with different randomly placed initial cluster centroids, and the iteration was selected which minimised the within-cluster sum of squares.[19] A range of models with a number of *k* clusters from 2 to 14 were compared using Davies-Bouldin (D-B) and Calinski-Harabasz (C-H) scores.[20, 21] Models with low D-B scores and high C-H scores were examined further: i) confusion matrices were constructed to compare cluster assignment in the model with *k* clusters against the model with *k+1* clusters and ii) mean values for each utilisation variable were calculated for each cluster in each model. Further details of the clustering approach are given in the online supplemental file, p5-17.

For peer review only

Statistical analysis

For the chosen clustering model, the resulting population segments were described by mean utilisation and characterised by age, sex, ethnicity, deprivation and LTC. Deprivation was calculated using quintiles of the Index of Multiple Deprivation (IMD) linked to patient postcode, with 1 representing the most deprived and 5 indicating the least deprived quintile.[22] Univariable associations were compared using chi squared tests. Adjusted multinomial logistic regression was used to identify characteristics associated with assignment to a particular segment relative to the lowest utilisation segment, including age, sex, ethnicity, deprivation and LTC in the model. The association between death and segment assignment was examined using univariable logistic regression. Missing data were low for gender (<0.1%) and IMD quintile (4.3%), but ethnicity was missing in 25.4% of cases. A sensitivity analysis of the multinomial model was carried out with the exclusion of ethnicity as a predictor.

Data were extracted from Discover using Microsoft SQL Server Management Studio 2012. Stata version 16.1 (StataCorp) was used for data manipulation, characterisation of segments and multinomial regression. Cluster analysis used Python version 3.6.8, Pandas version 1.2.0 and Scikit-learn version 0.23.2.[23]

Patient and Public Involvement

Patients or the public were not involved in this study.

Results

Population characteristics

A total of 429,496 CYP aged 0-15 years registered to GPs in Northwest London were identified in Discover. Of these, 378,266 (88.1%) participants were currently registered and had a full year of

For peer review only

follow-up between 01/03/2019 and 29/02/2020. An additional 43 children (0.01%) died during this time period, and a full year of follow-up was included preceding the date of death. A total of 378,309 participants were therefore included in the cohort.

Characteristics of the population are shown in Table 1. 51.3% of children were male, and 48.7% female with a mean (SD) age of 7.4 (4.6) years. Ethnicity was documented for 74.6% of participants, with the most common ethnic category being Asian or Asian British (24.5%), followed by 22.0% white, 11.0% mixed, 10.1% other ethnic groups and 6.9% black. There was a higher proportion of the population falling into more deprived areas by IMD quintile, with 16.0% in the most deprived, 32.0% in the second most deprived, and only 7.1% in the least deprived quintile. 35,946 (9.5%) were recorded as having one or more LTCs, with 32,963 (91.7%) of these having one LTC and 2,983 (8.3%) having more than one LTC. Asthma was the most commonly recorded chronic condition, with 21,659 prevalent cases (5.7%), followed by obesity (6,995; 1.9%), mental health problems (2,893; 0.8%), learning disabilities (1,607; 0.4%) and chronic heart conditions (1523; 0.4%).

Healthcare utilisation

264,755 (70.0%) CYP had at least one attendance to their GP over the year, with a mean (SD) number of attendances of 2.8 (3.9), and 19,990 (5.3%) children presenting ten or more times (Table 2). 136,424 (36.1%) were matched with one or more records of any secondary care attendance, with 23.5% attending A&E and 21.2% attending outpatients at least once over the year. 9,461 (2.5%) children had one or more elective admissions, with a mean of length of stay of 0.3 days per admission. 87.2% of those with at least one elective admission had no overnight admissions over the year. 12,078 (3.2%) children had at least one emergency admission, with a mean length of stay of 1.6 days per admission. Of those children admitted at least once as an emergency, 41.9% had no overnight admissions over the year.

For peer review only

Model selection

Comparison of D-B and C-H scores for a range of values of *k* clusters from 2 to 14 from k-means clustering on the seven health utilisation variables identified an optimal range from 4 to 8 clusters for further exploration (online supplemental file, Table S3). Mean utilisation measures were compared for each of the clusters in the 4- to 8-cluster models (online supplemental file, Figures S1-S5) and confusion matrices compared assignment to segments between clusters with increasing number of *k* clusters (online supplemental file, Figures S6-S9). Membership of clusters within the 6-cluster model were relatively consistent with respect to membership of clusters in the 5- and 7-cluster models. Additional increases in number of clusters to 7 or 8 did not add to the clinical utility given the additional complexity of interpretation.

Hierarchical agglomerative clustering using Ward's method was feasible on sub-samples of up to 50,000 participants. Evaluation of cluster membership on ten different random draws of data produced a mean V-measure score (a combination of homogeneity and completeness of cluster assignment) of 0.85 suggesting clustering was sensitive to sampling (online supplemental file, p6-7).[24] Clusters were characterised for the 6-cluster model using Ward's method and were broadly similar to those from the 6-cluster k-means model (online supplemental file, Figure S10 and Table S4). Descriptively, the both the 6-cluster k-means model and the 6-cluster hierarchical model partitioned according to the same variables, differing only in the magnitude of mean presentations, while the 'very low' utilisation group became a 'no-utilisation' group. Given the focus on describing the whole population, rather than a sub-sample, the 6-cluster k-means model was selected for further description.

Utilisation by segment

For peer review only - http://bmjopen.bmj.com/site/about/guidelines.xhtml

For the chosen 6-cluster model, the resulting population segments based on healthcare utilisation were described. Most patients fell into either a very low utilisation segment (I; 29.3%) with almost no healthcare utilisation, or into a low utilisation segment (II; 26.0%) attending a GP a mean (SD) of 1.4 (0.5) times in a year with very little additional utilisation (Table 3; medians (IQR) given in online supplemental file, table S5). Three smaller moderate utilisation segments were identified, with above average primary care utilisation. The largest of these (III), used primary care a mean (SD) of 5.2 (3.1) times per year, but had little other service use. The two remaining moderate utilisation segments were characterised by either above average elective care (IV; outpatient and elective admissions) or above average emergency care (V; A&E attendances and emergency admissions), attending primary care a mean (SD) of 4.2 (3.7) , or 5.0 (3.7) times, respectively.

A high utilisation segment (VI) was also identified, accounting for 16,632 children (4.4%), with the highest mean utilisation across all services (Table 2). Over 50% of all emergency admissions in the population, and total length of stay for elective and emergency admissions were accounted for by this group. Figure 1A shows the distribution of total activity across the seven utilisation variables for each segment. Figure 1B shows the distribution of total activity if all segments were of equal size, denoting the relative share of activity for a given individual within each segment.

Figure 1: a) Proportion of total activity for each healthcare utilisation variables accounted for by each segment: b) Relative contribution to total activity for each healthcare utilisation variable accounted for by a single individual in each segment assuming equal segment size

For peer review only

Costs per segment

Total healthcare costs for the cohort amounted to £140 million over the year with a mean of £372 per child, of which £92 was accounted for by primary care, and £280 by secondary care costs. Higher utilisation segments accounted for larger per capita costs and overall costs (online supplemental file, table S6). Mean per capita costs ranged from £32 in segment I to £1,222 in segment IV and £2,807 in segment VI. The lowest utilisation segment (I) represented 29.3% of the population but only 2.5% of total costs, and the highest utilisation segment (VI) for 4.4% of the population but 33.2% of total costs. Segment IV also accounted for 29.2% of total costs. Costs were not evenly distributed across care type: segment V accounted for 55.4% of A&E costs but only 19.4% of total costs and segment III represented the largest proportion of GP costs (33.3%) but only 10.4% of total costs.

Predictors of segment membership

Characteristics of each segment were described by age, sex, ethnicity, IMD quintile, and LTCs (online supplemental file, table S7). The gender balance differed across segments (p<0.001 from chi-squared) with segments IV-VI containing a higher proportion of males than females (54.0%, 53.2% and 56.0%, respectively). Higher utilisation segments tended to include younger children (p<0.001), with 17.3% and 17.5% aged under 1 year in segments V and VI, respectively, compared to only 1.9% under 1 in segment I. There was a strong association with deprivation (p<0.001) with 20.4% in the most deprived quintile and 5.6% in the least deprived in segment VI, compared to 13.8% in the most deprived and 7.5% in the least deprived in segment I. The proportion of the population with one or more LTCs increased across segments with increased utilisation (p<0.001), with only 3.6% in segment I, and 25.0% in segment VI. Children in the highest utilisation segment were significantly more likely to have died than those in the lowest utilisation segment (OR 21.4, 95% CI: 7.8 – 58.3), although 62.8% of all deaths still occurred outside the highest utilisation segment and 34.9% of deaths occurred in segments I-III.

For peer review only

Adjusted multinomial logistic regression was conducted to identify the predictors of segment membership. Relative risk ratios (RRR) are shown in Table 4. The relative risk of males being in segment VI compared to segment I, was 17% (95% CI: 12-21%) higher compared to females after adjusting for age, ethnicity, deprivation and LTC. Age was a strong predictor of segment membership, with those in younger age groups more likely to be in higher utilisation segments. Those aged 5-10 or 11-15 years had a 96% lower relative risk of being in the highest compared to the lowest utilisation segment, than those aged under 1 year after adjustment (RRR 95% CI: 4-5% and 3-4%, respectively). After adjustment, those of Asian or Asian British ethnicity were more likely to be in higher utilisation segments than those of other ethnicities, and with those of white ethnicity significantly less likely to be in each of the five higher utilisation segments.

A strong association was seen with higher levels of deprivation and membership of higher utilisation segments. Those with at least one LTC were 14.2 (RRR 95% CI: 13.4-15.1) times more likely to be in segment VI than segment I, 7.0 (RRR 95% CI: 6.7-7.4) times more likely to be in segment IV and 4.8 (RRR 95% CI: 4.6-5.1) times more likely to be in segment V, compared to those without any LTC. However, the majority of CYP in all segments had no documented LTC, with the highest proportion (25.0%) seen in segment VI. A sensitivity analysis excluding ethnicity (due to 25.4% missing data) showed no marked differences in coefficients for the remaining variables, indicating that ethnicity was not a confounder of these relationships.

Discussion

Using a data-driven approach, we defined six segments of the CYP population in Northwest London each based on patterns of healthcare utilisation. To our knowledge, this is the first application of segmentation approaches exclusive to the CYP population, including the whole population for a single geographic region. The highest utilisation group, representing less than 5% of the total population, accounted for over 50% of total emergency admissions and length of stay for the whole

For peer review only

population. Age and deprivation were strong predictors of segment membership, with a general tendency towards lower utilisation patterns in older age groups and higher utilisation patterns in children living in areas of higher deprivation. Increased utilisation segments were associated with higher total costs, with the two smallest segments, representing 13% of the population, accounting for almost two thirds of costs.

Those with one or more LTCs were significantly more likely to be in higher-utilisation segments than those without an LTC, but only one quarter of those in the high utilisation group had an LTC. Existing segmentation approaches applied to the adult population have focussed on defining segments based on demographics and LTCs, with some incorporating healthcare utilisation.[8] Although the two approaches are closely linked in adult populations, our findings suggest they are less tightly bound in the CYP population, with three quarters in the high utilisation segment not having a recorded LTC. As a result, segmentation based on demographic factors and clinical co-morbidities may fail to identify patients with higher rates of healthcare utilisation and vice versa, indicating a less well-defined trajectory of service use in children than in adults. Although this may make identification of high-need individuals using demographics and clinical factors more difficult, it suggests the potential for a greater impact of early interventions on healthcare utilisation and long-term outcomes.

The variation in healthcare utilisation with age is line with previous studies showing significantly lower rates of primary and secondary care attendances with increasing age, and the greatest use of primary care and emergency admissions in infants.[25, 26] Our study applied clustering to the whole CYP population and stratified clustering approaches to individual age groups might result in variation in the number and character of segments between age groups.

Implications for policy and practice

For peer review only

Our study demonstrates the feasibility and potential benefits of using a population segmentation approach to detect utilisation for CYP population health rather than reactive, condition-specific strategies which have often been used historically. More widespread use of segmentation models for CYP could lead to more effective and efficient strategies tailored towards local contexts. In addition, our findings provide robust empirical evidence of the ways in which segmentation and appropriate policy responses for each segment differ between CYP and adult populations. Commissioners and policymakers should ensure that there are separate local and national policies in place which recognise and take these differences between children and adults into account.

In the Northwest London context, we are currently utilising segmentation techniques to target interventions for two specific groups of children: infants, where ED attendances and primary care utilisation have increased significantly in the last 10 years[25] and those pre-schoolers who are frequent ED attenders who make up approximately 7% of attendees but account for 40% of the workload.[27]

Our findings of higher utilisation patterns in certain groups, such as Asian ethnic groups and those resident in more deprived areas highlight potential inequalities in health and access to services. Lack of access to one service can lead to a corresponding increase in utilisation of other services, with a recent study indicating a lower ratio of GP and outpatient care vs emergency and inpatient care in children from more deprived areas, and significantly higher primary care use in children from Asian ethnic groups.[28] In children, the factors impacting on healthcare utilisation are complex and multifactorial, involving psychosocial and mental health problems, school absence and parental anxiety and depression – which could not be incorporated in this study.[29, 30] Further work is needed to identify whether the results of our study indicate these populations to have greater and/or unmet health needs.

For peer review only

Implications for segmentation approaches in children

There are broader implications of these findings on the value of segmentation approaches in children. A limitation to the use of healthcare utilisation is that it may not accurately reflect healthcare need. A service may be indicated, but remain under-used, or may not be indicated, but used frequently, influenced by factors including proximity and access to services, to the requirement and ability to pay.[31]

Furthermore, extending from describing utilisation patterns to prediction of those with higher needs in order to intervene can unintentionally reinforce pre-existing inequities in access to healthcare. Obermeyer *et al* (2019) identified significant racial bias in an algorithm identifying need based on health costs, with Black patients showing higher rates of uncontrolled comorbid illness than White patients for a given risk score, due to misattribution of reduced access to care implying lower need.[32] Many of the factors related to utilisation patterns are unlikely to be captured in quantitative data, and further qualitative evaluations exploring child and parental factors related to utilisation patterns would be beneficial.[33]

Unlike with LTC-based segmentation, in which the conditions necessary for segment assignment are by definition 'chronic', healthcare utilisation will fluctuate over time. Identifying the trajectories of movement between segments might allow for early recognition and targeted intervention to reduce the risk of transition into a higher utilisation segment. In CYP, healthcare utilisation patterns are unlikely to be static, resulting in frequent movement between utilisation segments. This is supported by our finding that assignment to lower utilisation segments is more likely with increasing age and previous research showing that fewer than one third of children accounting for the top 5% of healthcare costs in England were in the same group the following year.[12] The COVID-19 pandemic is likely to alter utilisation patterns both in the short and long term, with rates of primary and secondary care emergency attendance and PICU admissions found to have fallen significantly in

For peer review only

Scotland.[34] Further research is needed to understand the transitions between utilisation segments and the extent to which these transitions can be predicted.

A core ambition of segmentation approaches is to identify those at greatest risk and intervene pre-emptively, but evidence for the success of such interventions is mixed, and focussed on adult populations. An intervention based around improving care coordination in the US for adults with heart failure at high risk of secondary care utilisation, demonstrated a reduction in A&E attendances and primary care visits, but not hospitalizations.[35] There is a risk in observational studies that by sampling high utilisers and following up over time, regression to the mean occurs, with over-estimation of the intervention effect. A recent randomized, controlled trial in the US targeting 'superutilizers' with care-coordination following discharge found no significant difference in readmission rates compared to usual care.[36] Within the child population, evidence is more limited. A population health management programme in the US targeted at under-21s in the top 10% of Medicaid expenditure showed a reduction in costs for those enrolled, of $378 per month per participant in the first year, but critically, showed no significant difference to a control group.[37]

For peer review only

Limitations

There are a number of limitations to the generalisability of the findings presented here. The use of Discover data represents a population in a single, localised geography and the findings may not be representative of the CYP population at a national or international level and certain groups may be under- or over- represented. The population of Northwest London includes four of the ten most ethnically diverse local authorities in England, with only 29.6% of those with non-missing data on ethnicity of white ethnicity, compared to 86.0% in England and Wales in 2011.[38, 39] There are also relatively more people resident in more deprived areas compared to the national average, with only 7.1% in the least deprived quintile of deprivation. Discover provides comprehensive population

coverage: 98% of the UK population are estimated to be registered to a GP, and 95% of those registered in Northwest London are captured in Discover.[13] However, it is likely that some of the most vulnerable groups, such as asylum seekers, refugees, travellers and homeless people, who may be disproportionate users of services such as emergency care, are not represented in these data.[40]

Use of linked routinely collected primary and secondary care datasets such as Discover, designed primarily for commissioning purposes and for direct clinical care, are still in their infancy for research purposes when compared with existing datasets such as the UK-based Clinical Practice Research Datalink (CPRD) and Hospital Episode Statistics (HES). Data on LTCs were collected from the primary care record, which relies on routine input from GPs. The validity of diagnostic coding in CPRD has been extensively investigated, with chronic diseases shown to be generally well-recorded.[41] Although no direct research has investigated coding validity in Discover, previous work has suggested a comparable prevalence of disease in Discover with national rates for those diseases defined by the Quality and Outcomes Framework (QOF).[13] However, many of the conditions relevant to LTCs in children do not form part of QOF, and so may be more likely to be incomplete, with evidence suggesting neurodevelopmental disorders in children, for example, are under-reported in the primary care record.[42] Obesity was prevalent in only 1.9% in our study, compared with a London average of 10.0% in reception children and 23.7% in Year 6 children, suggesting LTCs in our analysis may be under-estimated.[43] Total cost data were lower than national reported figures, with a recent report identifying annual costs of £113 and £380 in under 18s for primary and secondary care services, respectively in 2015-2016, compared with £92 and £280 in our study.[44] Cost data will also only measure part of the impact of service use, with quality of life and non-health related outcomes, including education, important metrics that were outside the scope of the current study.

For peer review only

The segmentation method used here was primarily data-driven; however, when using unsupervised clustering, there are no well-established criteria for choosing the optimal number of segments and a degree of subjectivity is inevitable.[9] Although metrics such as the Calinski-Harabasz and Davies-Bouldin scores, used here, give an indication as to the separation of clusters, there is no single metric which can determine the 'best' model, and different metrics may provide conflicting optima. Selection of the seven healthcare utilisation variables also impacts the cluster assignment; our model may weight inpatient admissions to a higher degree than other attendances, by including separately both elective and emergency admissions and lengths of stay. Different weightings can be applied to variables within the clustering process, to increase or decrease their relative importance, but there is no ground-truth from which to evaluate whether clusters defined in this way are better indicators of the utilisation in the population.

The defined segments are also not agnostic to the choice of clustering algorithm. Using a hierarchical approach with Ward's method on a subset of data, clusters were descriptively similar in terms of relative differences in utilisation, but with only an 85% match in terms of homogeneity and completeness. A disadvantage of k-means clustering is that changes to the number of segments can lead to changes in inclusion of previous clusters, although the six clusters appeared stable with respect to five or seven clusters, with new clusters in each case split from one or two larger clusters, lending the transition between k-means clustering outputs a quasi-hierarchical character. Use of hierarchical clustering would ensure the stability of clusters over a range of total clusters, but was not computationally feasible for the entire dataset.

Conclusion

As integrated care services focus on population health and preventive care, it is imperative that approaches are developed to identify those children and young people at greater risk. In this analysis, we describe how use of segmentation in the CYP population can identify distinct groups

For peer review only

based on different patterns of utilisation of services. Demographics and presence of long-term conditions may not explain the variation in healthcare utilisation in children to the same extent as in adults. This suggests a less well-defined trajectory of healthcare utilisation in children, which may indicate greater potential for the positive impact of early interventions. Further research is needed to understand both how CYP transition between utilisation segments, and whether additional factors available in routine EHRs can explain variation in utilisation, to enable early identification and intervention.

Acknowledgements

Many thanks to Eamon O'Doherty for his support with Discover access and data queries.

Ethics approval

Access to the Discover dataset was granted by the Northwest London Data Access Sub-group on 17/09/2020. Ethical approval was not required for this study as work was carried out for the purposes of service evaluation.

Funding

TB is supported by a National Institute for Health Research (NIHR) Academic Clinical Fellowship and by the NIHR Applied Research Collaboration (ARC) programme for Northwest London. JC and M Barahona acknowledge support from EPSRC grant EP/N014529/1 supporting the EPSRC Centre for Mathematics of Precision Healthcare. JC is also supported by the Wellcome Trust. [215938/Z/19/Z]. TW is supported by the NIHR ARC programme for Northwest London. The views expressed in this publication are those of the authors and not necessarily those of the NHS, the NIHR or the Department of Health.

Competing interests

For peer review only

The authors have no competing interests to declare.

Contributorship statement

TB, JC, M Blair and DSH conceived the study. TB, JC, TW, RM, KS, M Barahona, M Blair and DSH were involved in the design of the work and interpretation of the findings. TB conducted the cohort extraction and statistical analysis on Discover. TB and JC conducted the cluster analysis. TB wrote the first draft of the manuscript. TB, JC, TW, RM, KS, M Barahona, M Blair and DSH contributed to critical revisions of the manuscript and approved the final submission.

Data sharing statement

Data for this study is not publicly available, but access to pseudonymised data may be accessible for research purposes or service evaluation through application to the North West London Data Access Sub-Group.

References

1. Buck D, Baylis A, Dougall D, Robertson R. A vision for population health: Towards a healthier future. 2018. https://www.kingsfund.org.uk/publications/vision-population-health. Accessed 19 Dec 2020.

2. Gourevitch MN, Curtis LH, Durkin MS, Fagerlin A, Gelijns AC, Platt R, et al. The Emergence of Population Health in US Academic Medicine: A Qualitative Assessment. JAMA Netw Open. 2019;2:e192200–e192200. doi:10.1001/jamanetworkopen.2019.2200.

3. Kindig D, Stoddart G. What is population health? Am J Public Health. 2003;93:380–3.

4. NHS England. The NHS Long Term Plan. 2019. https://www.longtermplan.nhs.uk/wp-content/uploads/2019/08/nhs-long-term-plan-version-1.2.pdf. Accessed 6 Sep 2019.

5. Public Health England. Health matters: giving every child the best start in life. 2016. https://www.gov.uk/government/publications/health-matters-giving-every-child-the-best-start-in-

For peer review only

life/health-matters-giving-every-child-the-best-start-in-life. Accessed 28 Jan 2021.

6. Brommels M. Patient Segmentation: Adjust the Production Logic to the Medical Knowledge Applied and the Patient's Ability to Self-Manage—A Discussion Paper. Front Public Heal. 2020;8:195. doi:10.3389/fpubh.2020.00195.

7. Yoon S, Goh H, Kwan YH, Thumboo J, Low LL. Identifying optimal indicators and purposes of population segmentation through engagement of key stakeholders: a qualitative study. Heal Res Policy Syst. 2020;18:26. doi:10.1186/s12961-019-0519-x.

8. Chong JL, Lim KK, Matchar DB. Population segmentation based on healthcare needs: a systematic review. Syst Rev. 2019;8:202. doi:10.1186/s13643-019-1105-6.

9. Yan S, Kwan YH, Tan CS, Thumboo J, Low LL. A systematic review of the clinical application of data-driven population segmentation analysis. BMC Med Res Methodol. 2018;18:121. doi:10.1186/s12874-018-0584-9.

10. Vuik SI, Mayer E, Darzi A. A quantitative evidence base for population health: applying utilization-based cluster analysis to segment a patient population. Popul Health Metr. 2016;14:44. doi:10.1186/s12963-016-0115-z.

11. Dreyer K, Parry W, Jayatunga W, Deeny S. A descriptive analysis of health care use by high cost, high need patients in England. 2019. https://www.health.org.uk/publications/a-descriptive-analysis-of-health-care-use-by-high-cost-high-need-patients-in-england. Accessed 19 Dec 2020.

12. Punjabi N, Marszalek K, Beaney T, Shah R, Nicholls D, Deeny S, et al. Categorising high-cost high-need children and young people. Arch Dis Child. 2021. doi:10.1136/archdischild-2021-321654.

13. Bottle A, Cohen C, Lucas A, Saravanakumar K, Ul-Haq Z, Smith W, et al. How an electronic health record became a real-world research resource: comparison between London's Whole Systems Integrated Care database and the Clinical Practice Research Datalink. BMC Med Inform Decis Mak. 2020;20:71.

14. Curtis LA, Burns A. Unit Costs of Health and Social Care 2019. PSSRU, University of Kent; 2019. https://kar.kent.ac.uk/79286/.

For peer review only

15. NHS Improvement. Developing the national tariff. 2020.

16. NHS England. National Cost Collection: National Schedule of NHS costs - Year 2018-19 - NHS trust and NHS foundation trusts. National Cost Collection for the NHS. 2019.

17. Aitchison K, McGeown H, Holden B, Watson M, Klaber RE, Hargreaves D. Population child health: understanding and addressing complex health needs. Arch Dis Child. 2020. doi:10.1136/archdischild-2019-317373.

18. NHS England, British Medical Association. Seasonal influenza vaccination programme clinical used for payment. 2018. https://www.google.com/url?sa=t&rct=j&q=&esrc=s&source=web&cd=&cad=rja&uact=8&ved=2ahUKEwjBybDg5PXtAhUiVBUIHR34BxcQFjAJegQIAxAC&url=https%3A%2F%2Fwww.england.nhs.uk%2Fwp-content%2Fuploads%2F2019%2F07%2Fqsr-sfl-2019-20-codes-list.xlsm&usg=AOvVaw1Mt9mxgXhT_. Accessed 30 Dec 2020.

19. 2.3. Clustering — scikit-learn 0.24.1 documentation. https://scikit-learn.org/stable/modules/clustering.html#k-means. Accessed 29 Jan 2021.

20. Davies DL, Bouldin DW. A Cluster Separation Measure. IEEE Trans Pattern Anal Mach Intell. 1979;PAMI-1:224–7.

21. Caliński T, Harabasz J. A dendrite method for cluster analysis. Commun Stat. 1974;3:1–27. doi:10.1080/03610927408827101.

22. Ministry of Housing Communities & Local Government. English indices of deprivation 2015. National Statistics. 2015. https://www.gov.uk/government/statistics/english-indices-of-deprivation-2015. Accessed 23 Feb 2021.

23. Pedregosa F, Varoquaux G, Gramfort A, Michel V, Thirion B, Grisel O, et al. Scikit-learn: Machine Learning in Python. J Mach Learn Res. 2011;12:2825–30. http://jmlr.org/papers/v12/pedregosa11a.html.

24. Rosenberg A, Hirschberg J. V-Measure: A conditional entropy-based external cluster evaluation measure. EMNLP-CoNLL 2007 - Proc 2007 Jt Conf Empir Methods Nat Lang Process Comput Nat Lang

For peer review only

Learn. 2007; June:410–20.

25. Ruzangi J, Blair M, Cecil E, Greenfield G, Bottle A, Hargreaves DS, et al. Trends in healthcare use in children aged less than 15 years: a population-based cohort study in England from 2007 to 2017. BMJ Open. 2020;10. doi:10.1136/bmjopen-2019-033761.

26. Hobbs FDR, Bankhead C, Mukhtar T, Stevens S, Perera-Salazar R, Holt T, et al. Clinical workload in UK primary care: a retrospective analysis of 100 million consultations in England, 2007–14. Lancet. 2016;387:2323–30. doi:10.1016/S0140-6736(16)00620-6.

27. Blair M, Poots AJ, Lim V, Hiles S, Greenfield G, Crehan C, et al. Preschool children who are frequent attenders in emergency departments: an observational study of associated demographics and clinical characteristics. Arch Dis Child. 2018;103:19–23. doi:10.1136/archdischild-2016-311952.

28. Coughlan CH, Ruzangi J, Neale FK, Nezafat Maldonado B, Blair M, Bottle A, et al. Social and ethnic group differences in healthcare use by children aged 0{\textendash}14 years: a population-based cohort study in England from 2007 to 2017. Arch Dis Child. 2021. doi:10.1136/archdischild-2020-321045.

29. Al-Saffar MN, Hayhoe BW, Harris MJ, Majeed A, Greenfield G. Children as frequent attenders in primary care: a systematic review. BJGP open. 2020;4.

30. Dreyer K, Williamson RAP, Hargreaves DS, Rosen R, Deeny SR. Associations between parental mental health and other family factors and healthcare utilisation among children and young people: a retrospective, cross-sectional study of linked healthcare data. BMJ Paediatr open. 2018;2:e000266.

31. National Academies of Sciences Engineering and Medicine. Health-Care Utilization as a Proxy in Disability Determination. In: Health-Care Utilization as a Proxy in Disability Determination. Washington, DC: The National Academies Press; 2018.

32. Obermeyer Z, Powers B, Vogeli C, Mullainathan S. Dissecting racial bias in an algorithm used to manage the health of populations. Science (80-). 2019;366:447–53. doi:10.1126/science.aax2342.

33. Neill SJ. Containing acute childhood illness within family life: a substantive grounded theory. J child Heal care Prof Work with Child Hosp community. 2010;14:327–44.

For peer review only

34. Williams TC, MacRae C, Swann O V, Haseeb H, Cunningham S, Davies P, et al. Indirect effects of the COVID-19 pandemic on paediatric healthcare use and severe disease: a retrospective national cohort study. Arch Dis Child. 2021. doi:10.1136/archdischild-2020-321008.

35. David G, Smith-McLallen A, Ukert B. The effect of predictive analytics-driven interventions on healthcare utilization. J Health Econ. 2019;64:68–79. doi:https://doi.org/10.1016/j.jhealeco.2019.02.002.

36. Finkelstein A, Zhou A, Taubman S, Doyle J. Health Care Hotspotting — A Randomized, Controlled Trial. N Engl J Med. 2020;382:152–62. doi:10.1056/NEJMsa1906848.

37. Peele P, Keyser D, Lovelace J, Moss D. Advancing Value-Based Population Health Management Through Payer-Provider Partnerships: Improving Outcomes for Children With Complex Conditions. J Healthc Qual Off Publ Natl Assoc Healthc Qual. 2018;40:e26–32.

38. Office for National Statistics. Regional ethnic diversity. https://www.ethnicity-facts-figures.service.gov.uk/uk-population-by-ethnicity/national-and-regional-populations/regional-ethnic-diversity/latest#ethnic-diversity-by-area. Accessed 14 Aug 2020.

39. Office for National Statistics. Ethnicity and National Identity in England and Wales: 2011. 2012. https://www.ons.gov.uk/peoplepopulationandcommunity/culturalidentity/ethnicity/articles/ethnicityandnationalidentityinenglandandwales/2012-12-11. Accessed 26 Oct 2021.

40. Aspinall PJ. Hidden Needs: Identifying Key Vulnerable Groups in Data Collections: Vulnerable Migrants, Gypsies and Travellers, Homeless People, and Sex Workers. Incl Heal. 2014;:1–132. https://www.gov.uk/government/uploads/system/uploads/attachment_data/file/287805/vulnerable_groups_data_collections.pdf.

41. Khan NF, Harrison SE, Rose PW. Validity of diagnostic coding within the General Practice Research Database: a systematic review. Br J Gen Pract. 2010;60:e128--e136. doi:10.3399/bjgp10X483562.

42. Charlton RA, McGrogan A, Snowball J, Yates LM, Wood A, Clayton-Smith J, et al. Sensitivity of the UK Clinical Practice Research Datalink to Detect Neurodevelopmental Effects of Medicine Exposure

For peer review only

in Utero: Comparative Analysis of an Antiepileptic Drug-Exposed Cohort. Drug Saf. 2017;40:387–97. doi:10.1007/s40264-017-0506-5.

43. Public Health England. Obesity Profile. Public Health Profiles. 2020.

44. Kelly E, Lee T, Sibieta L, Waters T. Public Spending on Children in England: 2000 to 2020. 2018; June 2018. https://www.childrenscommissioner.gov.uk/wp-content/uploads/2018/06/Public-Spending-on-Children-in-England-CCO-JUNE-2018.pdf.

For peer review only

Table 1: Participant demographics, and long-term conditions at recruitment for all 378,309 participants

Participant characteristic		Total	Percentage
**Gender**	Female	184,408	48.7%
	Male	193,895	51.3%
	Unknown	6	<0.1%
**Age (years)**	Mean (SD)	7.4 (4.6)	
	Median (IQR)	7 (3-11)	
	Under 1	28,304	7.5%
	1-2	44,590	11.8%
	3-4	45,559	12.0%
	5-10	148025	39.1%
	11-15	111,831	29.6%
**Ethnicity**	Asian or Asian British	92,704	24.5%
	White	83,406	22.0%
	Mixed/multiple ethnic groups	41,727	11.0%
	Other ethnic groups	38,279	10.1%
	Black or Black British	25,959	6.9%
	Unknown	96234	25.4%
**IMD quintile**	1 (most deprived)	60,506	16.0%
	2	121,174	32.0%
	3	96,213	25.4%
	4	57,445	15.2%
	5 (least deprived)	26,713	7.1%
	Unknown	16,258	4.3%
**Number of long-term conditions (LTC)**	0	341,911	90.4%
	1	32,963	8.7%
	2	2,724	0.7%
	3	235	0.1%
	4 or more	24	<0.1%
	Missing data	452	0.1%
**Long-term conditions***	Asthma	21,659	5.7%
	Obesity	6995	1.9%
	Mental health problem	2893	0.8%
	Learning Disability	1607	0.4%
	Chronic heart disease	1523	0.4%
	Chronic neurological disease & epilepsy	1309	0.3%
	Hypothyroidism	1088	0.3%
	Cancer & immunosuppression	1061	0.3%
	Diabetes	775	0.2%
	Chronic respiratory disease (non-asthma)	126	<0.1%
	Chronic kidney disease	126	<0.1%
	Palliative care	36	<0.1%
	Rheumatoid arthritis	18	<0.1%
**Total participants**		**378,309**	

* Proportion of those with non-missing data on LTC

For peer review only

Table 2: Healthcare utilisation over one year for all 378,309 participants

Healthcare utilisation		Total	Percentage
GP attendances	Mean (SD)	2.8 (3.9)	
	Median (IQR)	2 (0-4)	
	0	113,554	30.0%
	1	70,666	18.7%
	2-4	116,328	30.7%
	5-9	57,771	15.3%
	10+	19,990	5.3%
A&E attendances	Mean (SD)	0.4 (1.0)	
	Median (IQR)	0 (0-0)	
	0	289,316	76.5%
	1	51,972	13.7%
	2-4	33,022	8.7%
	5-9	3,724	1.0%
	10+	275	0.1%
Outpatient attendances	Mean (SD)	0.7 (2.3)	
	Median (IQR)	0 (0-0)	
	0	298,289	78.8%
	1	28,437	7.5%
	2-4	35,014	9.3%
	5-9	12,448	3.3%
	10+	4,121	1.1%
Elective admissions	Mean (SD)	0.04 (0.40)	
	Median (IQR)	0 (0-0)	
	0	368,848	97.5%
	1-2	7,498	2.0%
	3-4	1,709	0.5%
	5-9	158	<0.1%
	10+	96	<0.1%
Total elective length of stay (days) ^	Mean (SD)	0.24 (0.98)	
	Median (IQR)	0 (0-0)	
	0	8,248	87.2%
	1	822	8.7%
	2-4	256	2.7%
	5-9	76	0.8%
	10+	59	0.6%
Emergency admissions	Mean (SD)	0.04 (0.28)	
	Median (IQR)	0 (0-0)	
	0	366,231	96.8%
	1-2	10,054	2.7%
	3-4	1,902	0.5%
	5-9	108	<0.1%
	10+	14	<0.1%

For peer review only

	Mean (SD)	1.6 (4.2)	
	Median (IQR)	1 (0-2)	
**Total emergency length of stay (days) ^**	0	5,058	41.9%
	1-2	3,778	31.3%
	3-4	2,302	19.1%
	5-9	647	5.4%
	10-14	293	2.4%
**Total participants**		**378,309**	

* Proportion of those with non-missing data on LTC

^ percentages of those admitted at least once

For peer review only

Table 3: Mean presentations/length of stay over one year for each segment from 6 cluster k-means model (green indicates lower than average utilisation, red indicates higher than average utilisation)

Segment	Size	Percentage	GP	Outpatient	A&E	Elective admissions	Elective length of stay	Emergency admissions	Emergency length of stay
I: Very low	110869	29.3%	0.00	0.09	0.07	0.01	0.00	0.00	0.00
II: Low; GP	98293	26.0%	1.36	0.06	0.07	0.01	0.00	0.00	0.00
III: Moderate; GP	67358	17.8%	5.19	0.14	0.00	0.01	0.00	0.00	0.00
IV: Moderate; GP, OP & elective	33633	8.9%	4.23	4.47	0.22	0.15	0.03	0.01	0.01
V: Moderate; GP & emergency	51524	13.6%	4.97	0.17	1.75	0.02	0.00	0.11	0.08
VI: High; all services	16632	4.4%	10.14	5.01	2.78	0.32	0.14	0.52	0.89
**Total**	**378309**		**2.78**	**0.71**	**0.42**	**0.04**	**0.01**	**0.04**	**0.05**

Table 4: Adjusted multinomial logistic regression of factors associated with segment assignment for 269,248 participants with non-missing data

Segment		Relative risk ratio	Standard error	z-score	p-value	95% confidence interval	
I: Very low	**Gender**	Base outcomes					
	Female						
	**Age (years)**						
	Under 1						
	**Ethnicity**						
	Asian or Asian British						
	**IMD quintile**						
	1 (most deprived)						
	**Long term conditions**						
	None						
II: Low; GP	**Gender**						
	Male	0.93	0.01	-7.29	<0.001	0.91	0.95
	**Age (years)**						
	1-2	0.90	0.04	-2.75	0.01	0.83	0.97
	3-4	0.65	0.03	-11.13	<0.001	0.60	0.70
	5-10	0.39	0.01	-26.36	<0.001	0.37	0.42
	11-15	0.30	0.01	-33.68	<0.001	0.28	0.32
	**Ethnicity**						
	White	0.74	0.01	-22.10	<0.001	0.73	0.76
	Mixed	1.06	0.02	3.20	<0.001	1.02	1.09
	Other ethnic groups	0.83	0.01	-10.52	<0.001	0.80	0.86
	Black or Black British	0.85	0.02	-8.10	<0.001	0.82	0.88
	**IMD quintile**						
	2	0.89	0.01	-7.45	<0.001	0.86	0.91
	3	0.83	0.01	-11.27	<0.001	0.80	0.85
	4	0.78	0.01	-13.33	<0.001	0.75	0.81
	5 (least deprived)	0.82	0.02	-8.10	<0.001	0.79	0.86
	**Long term conditions**						
	One or more	2.29	0.05	35.17	<0.001	2.19	2.40
	Constant	2.96	0.11	28.26	<0.001	2.74	3.19
III: Moderate; GP	**Gender**				<0.001		
	Male	0.91	0.01	-8.23	<0.001	0.88	0.93
	**Age (years)**				<0.001		
	1-2	0.50	0.02	-18.60	<0.001	0.46	0.54
	3-4	0.26	0.01	-36.82	<0.001	0.24	0.28

For peer review only

	5-10	0.09	0.00	-73.43	<0.001	0.08	0.09
	11-15	0.07	0.00	-78.84	<0.001	0.06	0.07
	**Ethnicity**				<0.001		
	White	0.49	0.01	-45.98	<0.001	0.48	0.51
	Mixed	0.70	0.01	-18.41	<0.001	0.67	0.73
	Other ethnic groups	0.67	0.01	-20.45	<0.001	0.64	0.70
	Black or Black British	0.63	0.01	-19.98	<0.001	0.60	0.66
	**IMD quintile**				<0.001		
	2	0.88	0.02	-6.80	<0.001	0.85	0.91
	3	0.77	0.01	-13.37	<0.001	0.74	0.80
	4	0.67	0.01	-18.28	<0.001	0.64	0.70
	5 (least deprived)	0.82	0.02	-7.37	<0.001	0.77	0.86
	**Long term conditions**				<0.001		
	One or more	5.80	0.13	75.59	<0.001	5.54	6.07
	Constant	8.00	0.29	56.48	<0.001	7.44	8.59
**IV: Moderate; GP, OP & elective**	**Gender**				<0.001		
	Male	1.08	0.02	5.00	<0.001	1.05	1.11
	**Age (years)**				<0.001		
	1-2	0.57	0.03	-11.84	<0.001	0.51	0.62
	3-4	0.41	0.02	-19.52	<0.001	0.37	0.44
	5-10	0.20	0.01	-38.29	<0.001	0.19	0.22
	11-15	0.21	0.01	-37.12	<0.001	0.19	0.23
	**Ethnicity**				<0.001		
	White	0.78	0.02	-12.86	<0.001	0.75	0.81
	Mixed	1.05	0.03	1.96	0.05	1.00	1.10
	Other ethnic groups	0.96	0.02	-1.66	0.10	0.92	1.01
	Black or Black British	0.96	0.03	-1.30	0.19	0.91	1.02
	**IMD quintile**						
	2	0.73	0.02	-14.28	<0.001	0.70	0.77
	3	0.64	0.01	-19.54	<0.001	0.61	0.67
	4	0.55	0.01	-22.59	<0.001	0.52	0.58
	5 (least deprived)	0.69	0.02	-11.10	<0.001	0.65	0.74
	**Long term conditions**				<0.001		
	One or more	7.01	0.17	78.05	<0.001	6.67	7.36
	Constant	1.53	0.07	9.31	<0.001	1.40	1.68
**V: Moderate; GP & emergency**	**Gender**				<0.001		
	Male	1.06	0.01	4.13	<0.001	1.03	1.08
	**Age (years)**				<0.001		
	1-2	0.39	0.01	-25.17	<0.001	0.36	0.42
	3-4	0.18	0.01	-46.40	<0.001	0.17	0.20
	5-10	0.06	0.00	-84.94	<0.001	0.06	0.06

For peer review only

	11-15	0.04	0.00	-92.34	<0.001	0.04	0.04
	**Ethnicity**				<0.001		
	White	0.66	0.01	-24.96	<0.001	0.63	0.68
	Mixed	0.97	0.02	-1.70	0.09	0.93	1.01
	Other ethnic groups	0.81	0.02	-9.64	<0.001	0.78	0.85
	Black or Black British	0.73	0.02	-12.05	<0.001	0.70	0.77
	**IMD quintile**				<0.001		
	2	0.80	0.02	-11.67	<0.001	0.77	0.83
	3	0.69	0.01	-17.75	<0.001	0.67	0.72
	4	0.56	0.01	-24.17	<0.001	0.54	0.59
	5 (least deprived)	0.59	0.02	-16.85	<0.001	0.56	0.63
	**Long term conditions**				<0.001		
	One or more	4.84	0.12	61.75	<0.001	4.61	5.09
	Constant	7.59	0.28	54.18	<0.001	7.06	8.17
**VI: High; all services**	**Gender**						
	Male	1.17	0.02	7.55	<0.001	1.12	1.21
	**Age (years)**				<0.001		
	1-2	0.34	0.02	-23.77	<0.001	0.31	0.37
	3-4	0.13	0.01	-42.88	<0.001	0.12	0.15
	5-10	0.04	0.00	-76.73	<0.001	0.04	0.05
	11-15	0.04	0.00	-77.55	<0.001	0.03	0.04
	**Ethnicity**				<0.001		
	White	0.69	0.02	-14.05	<0.001	0.65	0.73
	Mixed	0.97	0.03	-0.89	0.38	0.91	1.03
	Other ethnic groups	0.91	0.03	-2.87	<0.001	0.86	0.97
	Black or Black British	0.85	0.03	-4.20	<0.001	0.79	0.92
	**IMD quintile**				<0.001		
	2	0.72	0.02	-11.83	<0.001	0.68	0.76
	3	0.58	0.02	-17.84	<0.001	0.55	0.62
	4	0.43	0.02	-23.07	<0.001	0.40	0.46
	5 (least deprived)	0.50	0.02	-14.11	<0.001	0.46	0.55
	**Long term conditions**				<0.001		
	One or more	14.23	0.42	90.16	<0.001	13.44	15.08
	Constant	2.55	0.12	19.84	<0.001	2.32	2.79

For peer review only

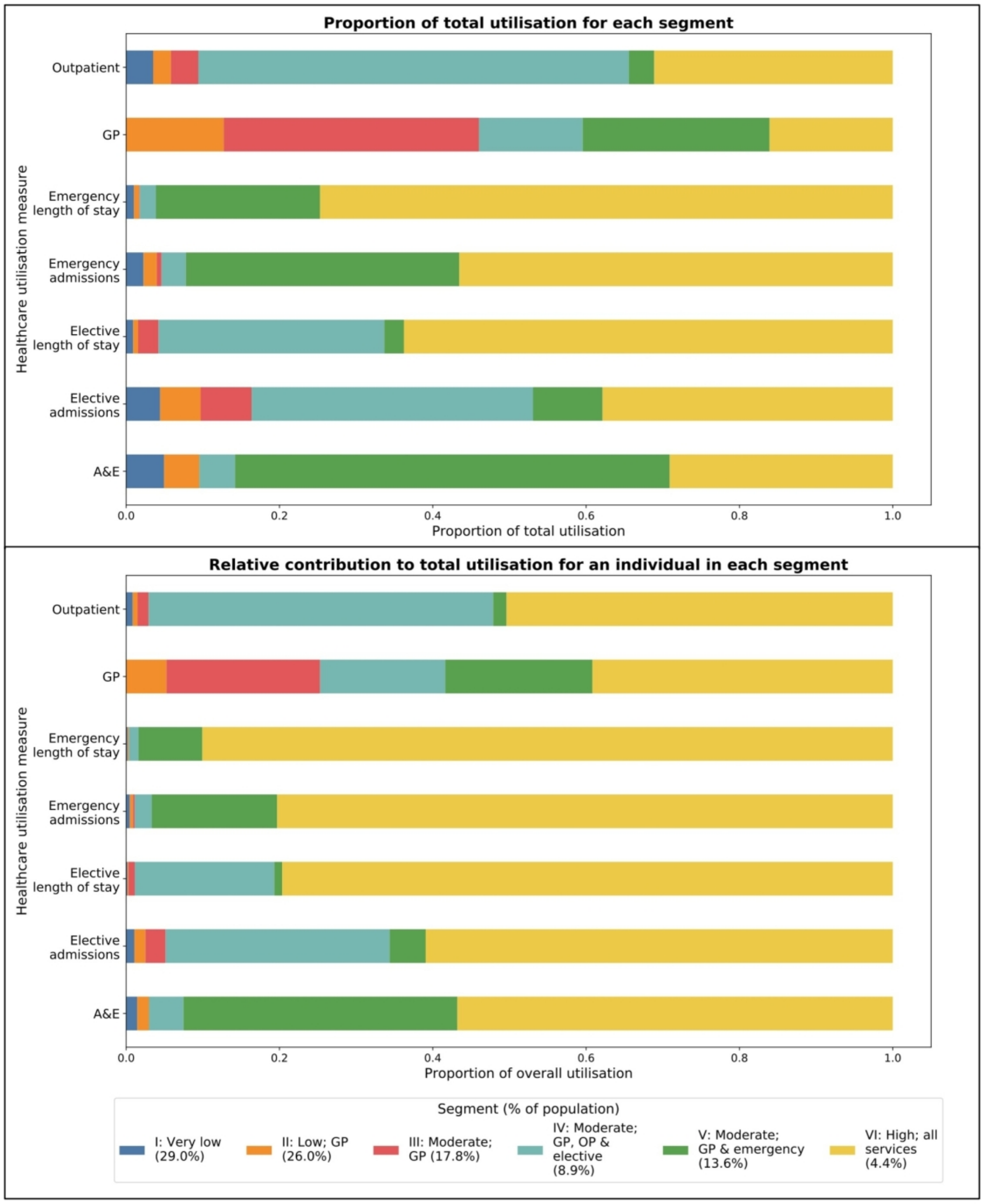

Figure 1: a) Proportion of total activity for each healthcare utilisation variables accounted for by each segment: b) Relative contribution to total activity for each healthcare utilisation variable accounted for by a single individual in each segment assuming equal segment size

160x195mm (300 x 300 DPI)

Patterns of healthcare utilisation in children and young people: a retrospective cohort study using routinely collected healthcare data in Northwest London

Thomas Beaney[1,2]

Jonathan M Clarke[3]

Thomas Woodcock[1,2]

Rachel McCarthy[4]

Kavitha Saravanakumar[4]

Mauricio Barahona[3]

Mitch Blair[1,2]

Dougal Hargreaves[1,2]

1. Department of Primary Care and Public Health, Imperial College London, London, W6 8RP, UK.
2. National Institute for Health Research Applied Research Collaboration Northwest London, Imperial College, London, W6 8RP, UK.
3. Department of Mathematics and Centre for Mathematics of Precision Healthcare, Imperial College London, London, SW7 2AZ, UK.
4. North West London Collaboration of Clinical Commissioning Groups, London, NW1 5JD, UK

Corresponding author:

Thomas Beaney

Department of Primary Care and Public Health, Imperial College London, London, W6 8RP, UK.

Email: Thomas.beaney@imperial.ac.uk

For peer review only

Appendix

Patterns of healthcare utilisation in children and young people: a retrospective cohort study using routinely collected healthcare data in Northwest London

Note: some of the text in the appendix is repeated from the manuscript to provide further detail.

Healthcare utilisation

Seven healthcare utilisation measures were selected for inclusion:

1. GP attendances
2. A&E attendances
3. Outpatient attendances
4. Elective hospital admissions
5. Total length of stay from elective admissions in days
6. Emergency hospital admissions
7. Total length of stay from emergency admissions in days

Numbers of attendances or admissions were summed across the year. GP attendances included all consultation types collectively (eg. face to face, telephone, home visits) and staff types (eg. GP, nurse) due to variable recording of these factors in the Discover dataset, with exclusion of events linked only to administrative Read codes, and includes visits for preventive reasons. Where more than one event of the same type occurred on a single date, only one attendance was counted. Total length of stay was calculated as the total number of inpatient days across the year, separately for elective and emergency admissions. Where a patient was admitted and discharged on the same day, the length of stay for that admission is recorded as zero days.

For peer review only

Long-term conditions

Discover includes a long-term condition (LTC) table, listing 41 chronic conditions based on Read V2 codes. A sub-set of 17 of these were selected for inclusion based on Aitchison *et al* (2020), who identified relevant LTCs from Discover applicable to children (Table A1).[1] Certain important diagnostic categories, such as congenital heart disease are not included in the Discover LTC list and so additional diagnosis codes were sourced. Raw Read codes are translated into SNOMED in the GP events table on Discover. Additional SNOMED code clusters were extracted for asthma, obesity, congenital heart disease, chronic kidney disease, diabetes, immunosuppression, learning disability, liver disease, neurological disease and chronic respiratory disease based on available code clusters listed by NHS as risk factors for influenza vaccination.[2] Chronic heart disease codes were combined with coronary heart disease and heart failure, and chronic neurological disease was combined with cerebral palsy, epilepsy and spina bifida (Table A2). Number of LTCs were counted as the number of categories of LTC from Table A3. Relevant codes were identified from the entirety of the patient primary care record and secondary care diagnostic codes were not included in order not to bias number of conditions in favour of those presenting to secondary care.

For peer review only

Table S1: 41 Long-term conditions identified in Discover (included conditions highlighted)

**Asthma ***	**Hypothyroidism ***
Atrial Fibrillation	**Anxiety ***
**Cancer ***	Charcot Marie Tooth Disease
**CKD ***	Dystonia Primary Idiopathic
COPD	Essential Tremor
Dementia	Huntingtons Disease
**Depression ***	Motor Neurone Disease
**Diabetes ***	Multiple Sclerosis
**Epilepsy ***	Multiple System Atrophy
**Heart Failure ***	Muscular Dystrophy
Hypertension	Myalgic Encephalopmyelitis
**Learning Disability ***	Myasthenia Gravis
**Mental Health ***	Parkinsons Disease
**Obesity ***	Progressive Supranuclear Palsy
Osteoporosis	Transverse Myelitis
Peripheral Arterial Disease	**Cerebal Palsy ***
**Palliative care ***	**Spina Bifida and Hydrocephalus ***
**Rheumatoid Arthritis ***	Ataxia
**Coronary Heart Disease ***	Ataxia-Telangiectasia
Stroke/TIA	Encephalitis
Ischaemic Heart Disease	

* Included in analysis

Table S2: Defined long-term conditions and source of codes from Discover and SNOMED

**Long-term condition (LTC)**	**Discover LTC table**	**Additional SNOMED clusters**[2]
Asthma	Asthma	Asthma
Obesity	Obesity	Obesity
Mental health condition	Anxiety; depression; mental health	-
Learning Disability	Learning disability	Learning disability
Chronic heart disease	Coronary heart disease; heart failure	Chronic heart disease
Chronic neurological disease & epilepsy	Cerebral palsy; epilepsy; spina bifida	Chronic neurological disease
Hypothyroidism	Hypothyroidism	-
Cancer & immunosuppression	Cancer	Immunosuppression
Diabetes	Diabetes	Diabetes
Chronic respiratory disease (non-asthma)	-	Chronic respiratory disease
Chronic kidney disease	Chronic kidney disease	Chronic kidney disease
Palliative care	Palliative care	-
Rheumatoid arthritis	Rheumatoid arthritis	-

For peer review only

Selection of number of clusters

Calinski-Harabasz and Davies-Bouldin scores were calculated for *k* in the range 2-14.[3, 4] The former calculates the ratio of between-cluster sum of squared distances to the within-cluster sum of squared distances, with larger values indicating better dispersion. The latter calculates the average similarity of clusters, based on the average distance of each point in the cluster to the centroid, and the distance between cluster centroids, with smaller values indicating better separation. Table S3 displays both scores by value of *k*.

Table S3: Calinski-Harabasz and Davies-Bouldin scores for values of *k* clusters

k	Calinski-Harabasz	Davies-Bouldin
2	237582	1.09368
3	221031	1.15472
4	228535	0.99956
5	212661	1.07835
6	211027	0.99648
7	205075	1.00265
8	199811	1.03196
9	193744	1.02996
10	187409	1.07746
11	184038	1.04232
12	180593	1.01880
13	176750	1.02698
14	173582	1.02538

The Calinski-Harabasz score was highest for two clusters and declined gradually from four clusters, indicating better dispersion with a smaller number of clusters. The Davies-Bouldin score was lowest for six clusters, with similarly low scores for four, seven and eight clusters. The range from four to eight were selected as a focus for further description. Figures S1-S5 show the distributions of mean activity across the seven healthcare utilisation variables for each of the five models. Confusion matrices (Figures S6-S9) show the changes in assignment between consecutive increases in *k* clusters.

For peer review only

Utilisation within each cluster was assigned a descriptor based on total utilisation (low if mean attendances below average for all attendance types; medium if above average for at least one and fewer than four attendance types; and high if above average for four or more attendance types) and predominant attendance types accounting for utilisation.

In a 4-cluster model (Figures S1a and S1b), a low utilisation group and a moderate, GP predominant segment were formed. Two high utilisation segments were also formed, one utilising predominantly GP and emergency care, and the other using all services. The 5-cluster model (Figures S2a and S2b) retained the first two clusters, but split the remaining higher utilisation clusters into a moderate; GP, outpatient and elective care segment, and a moderate utilisation; GP and emergency care group, retaining in addition a smaller high utilisation cluster. The 6-cluster (Figures S3a and S3b) model split the very low and the moderate; GP clusters into a new low utilisation; GP cluster.

The 7-cluster model (Figures S4a and S4b) split three segments (very low; low utilisation, GP; and moderate utilisation, GP and emergency care) into an additional low utilisation group characterised by lower than average GP use, but higher emergency care use. The 8-cluster model (Figures S5a and S5b) split the moderate utilisation GP, outpatient and elective care group into an additional moderate utilisation cluster characterised by lower than average GP use, but higher elective care use. Participants in other segments from the 7-cluster model were also reassigned on increasing to 8-clusters, suggesting inherently less stable clusters. The additional clusters produced by the 7- and 8-cluster models were decided to be less useful clinically as descriptors given the added complexity of the model, and the 6-cluster model was selected as the optimal number of segments.

Hierarchical clustering

Hierarchical agglomerative clustering using Ward's method was computationally feasible on sub-samples of up to 50,000 participants (13.2% of the total sample). In order to compare the similarity of cluster assignment between different random draws, a fixed sub-sample of 5,000 participants was randomly selected (R_F). The clustering algorithm was then iterated 10 times. On each iteration, a new

For peer review only - http://bmjopen.bmj.com/site/about/guidelines.xhtml

sub-sample of 45,000 participants (R_V) was randomly selected, added to R_F to create a total sub-sample of 50,000 participants (R_F + R_V). Pair-wise comparisons of cluster membership for participants in R_F from each iteration compared to the preceding iteration were made using V-measure scores, the harmonic mean of homogeneity and completeness scores.[5] A range of V-measure scores from 0.765 – 0.918 were calculated, with a mean of 0.847, suggesting that cluster assignment was sensitive the sub-sample of data used.

For peer review only

Figure S1a: Mean number of attendances to outpatients, GP and A&E from the 4 cluster model

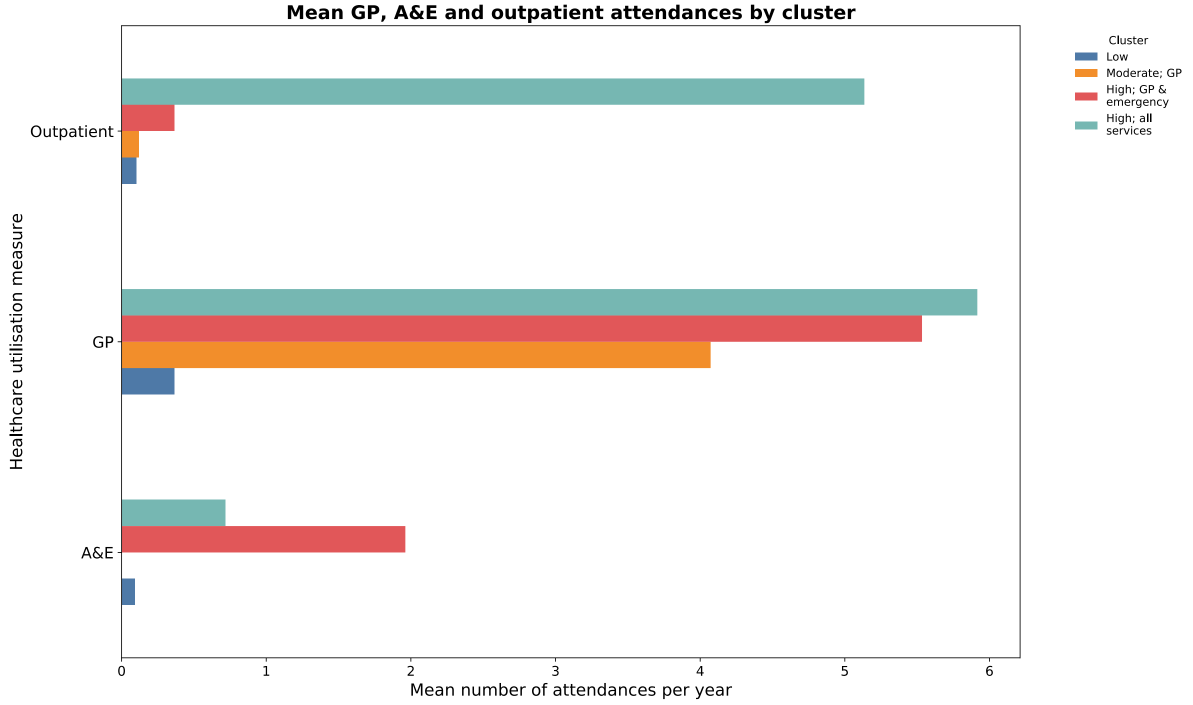

Figure S1b: Mean number of elective and emergency admission and yearly length of stay from the 4 cluster model

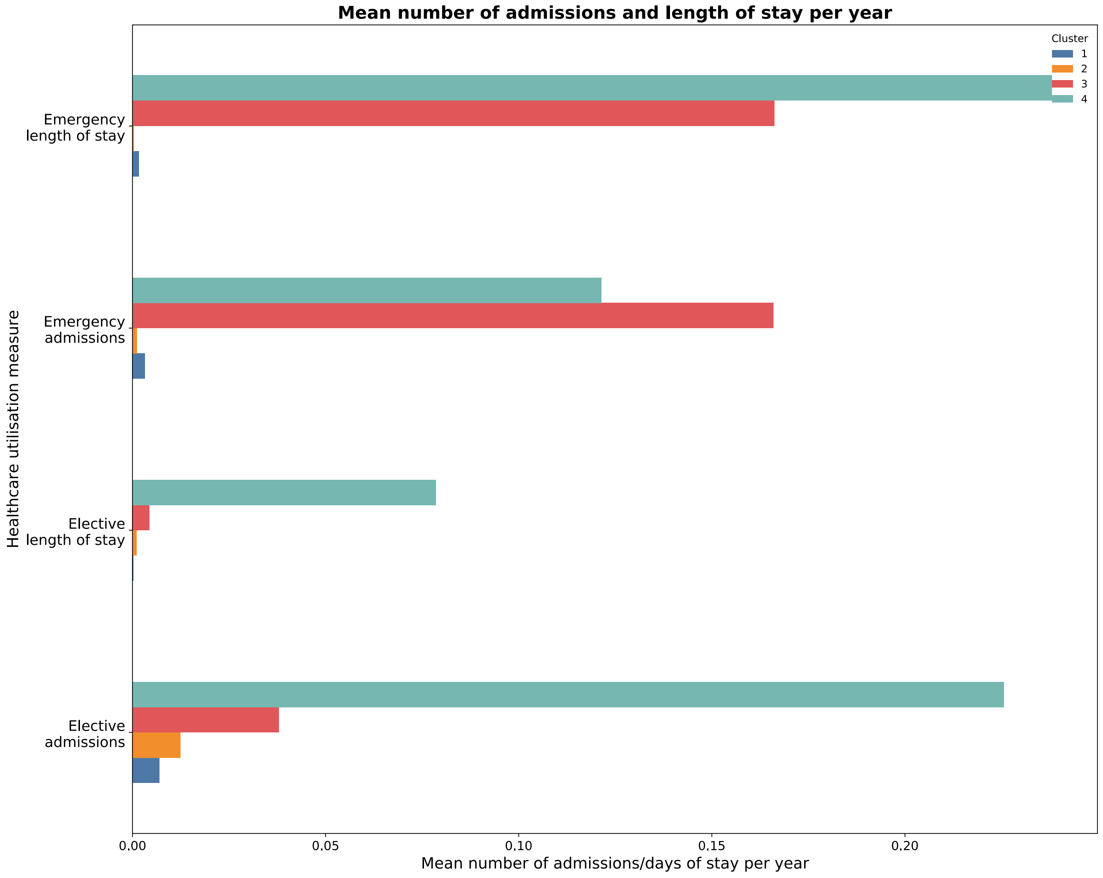

For peer review only - http://bmjopen.bmj.com/site/about/guidelines.xhtml

Figure S2a: Mean number of attendances to outpatients, GP and A&E from the 5 cluster model

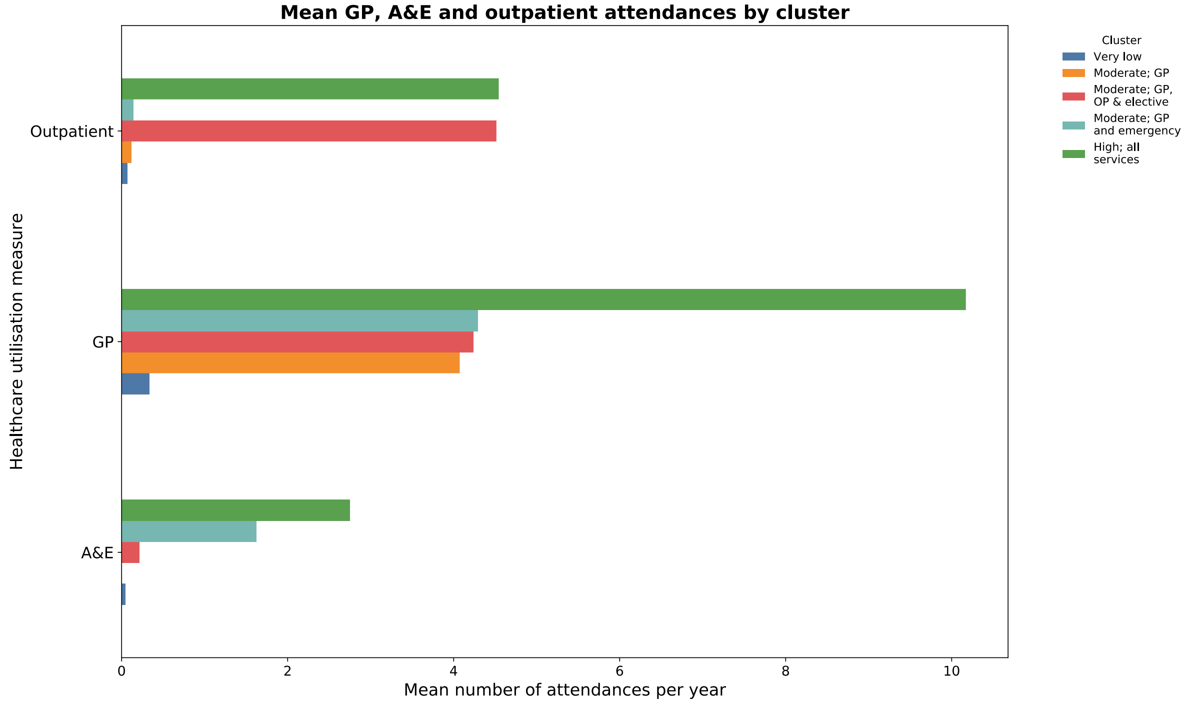

Figure S2b: Mean number of elective and emergency admission and yearly length of stay from the 5 cluster model

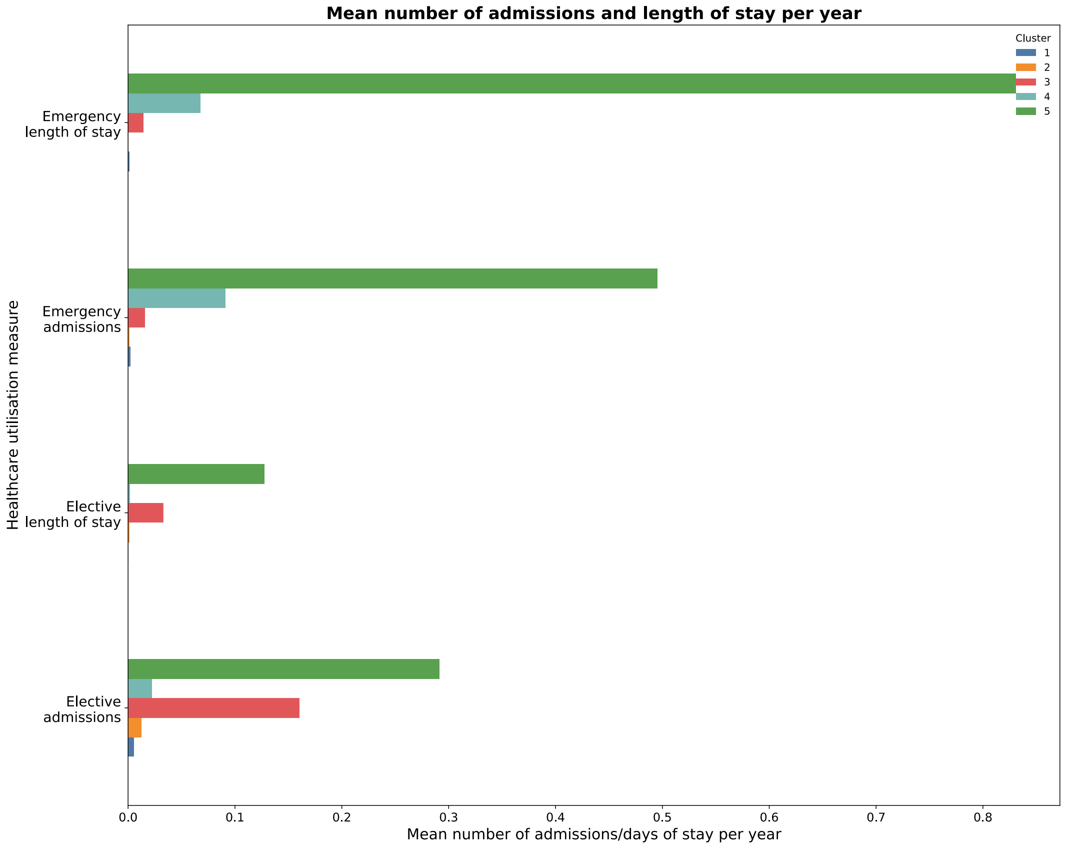

For peer review only - http://bmjopen.bmj.com/site/about/guidelines.xhtml

Figure S3a: Mean number of attendances to outpatients, GP and A&E from the 6 cluster model

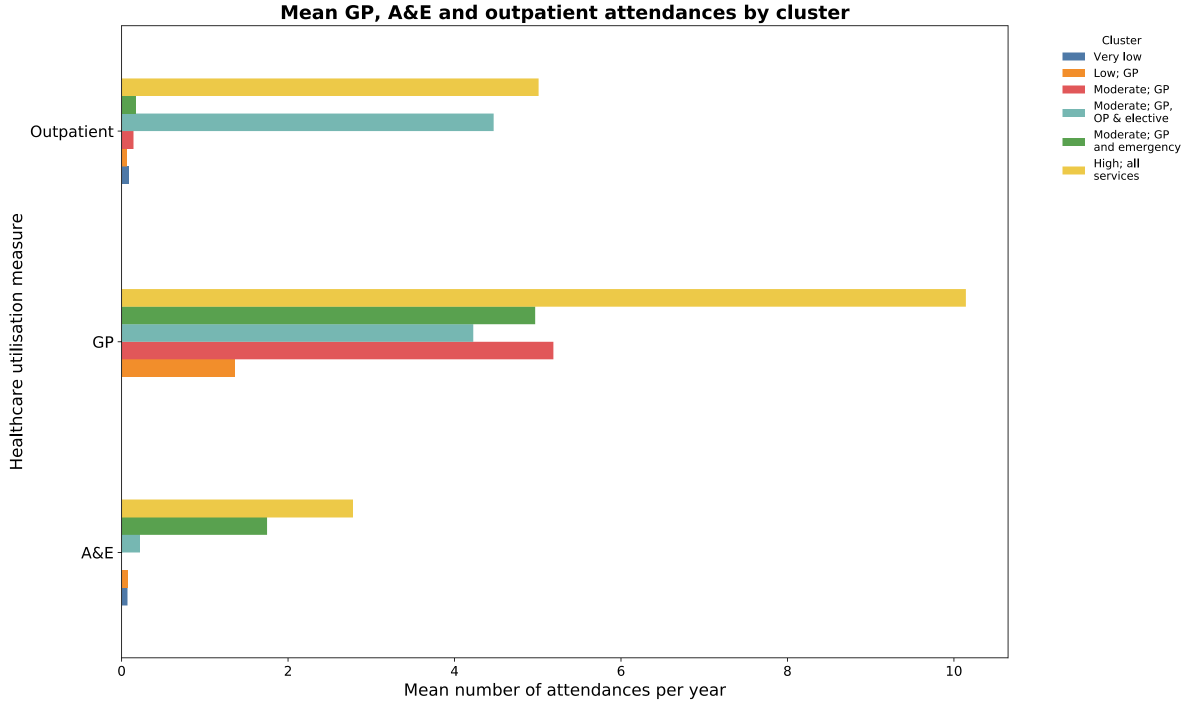

Figure S3b: Mean number of elective and emergency admission and yearly length of stay from the 6 cluster model

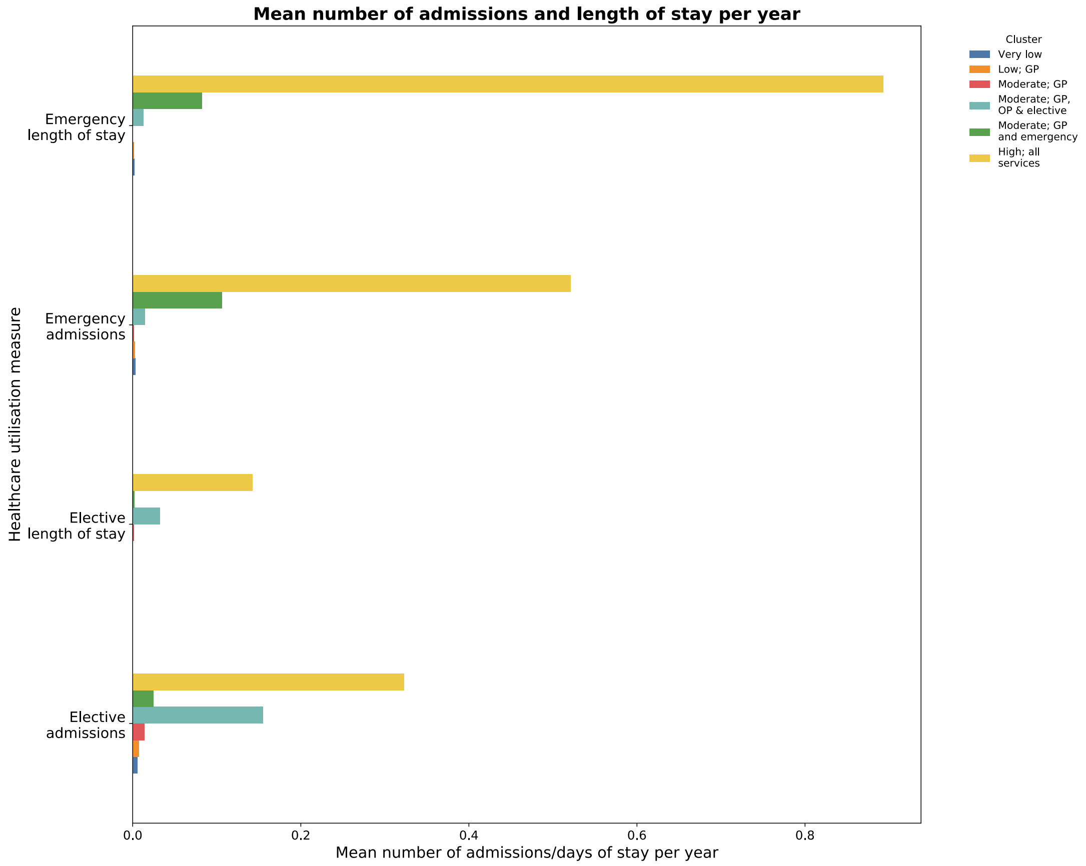

For peer review only - http://bmjopen.bmj.com/site/about/guidelines.xhtml

Figure S4a: Mean number of attendances to outpatients, GP and A&E from the 7 cluster model

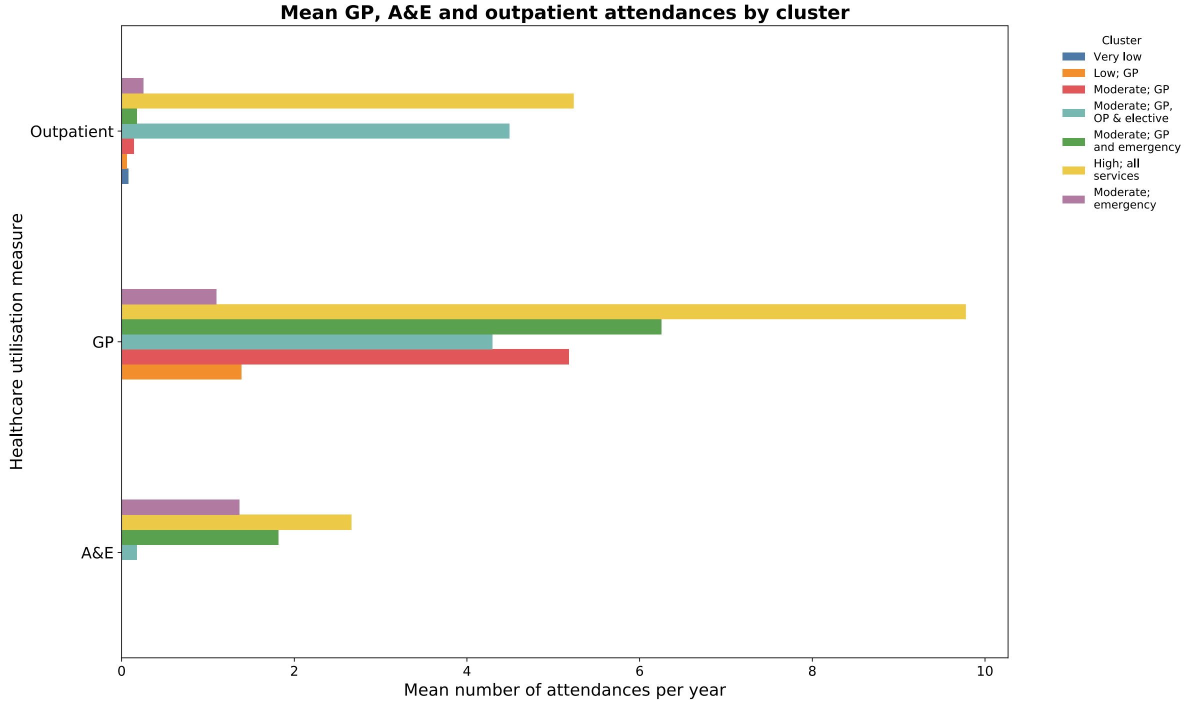

Figure S4b: Mean number of elective and emergency admission and yearly length of stay from the 7 cluster model

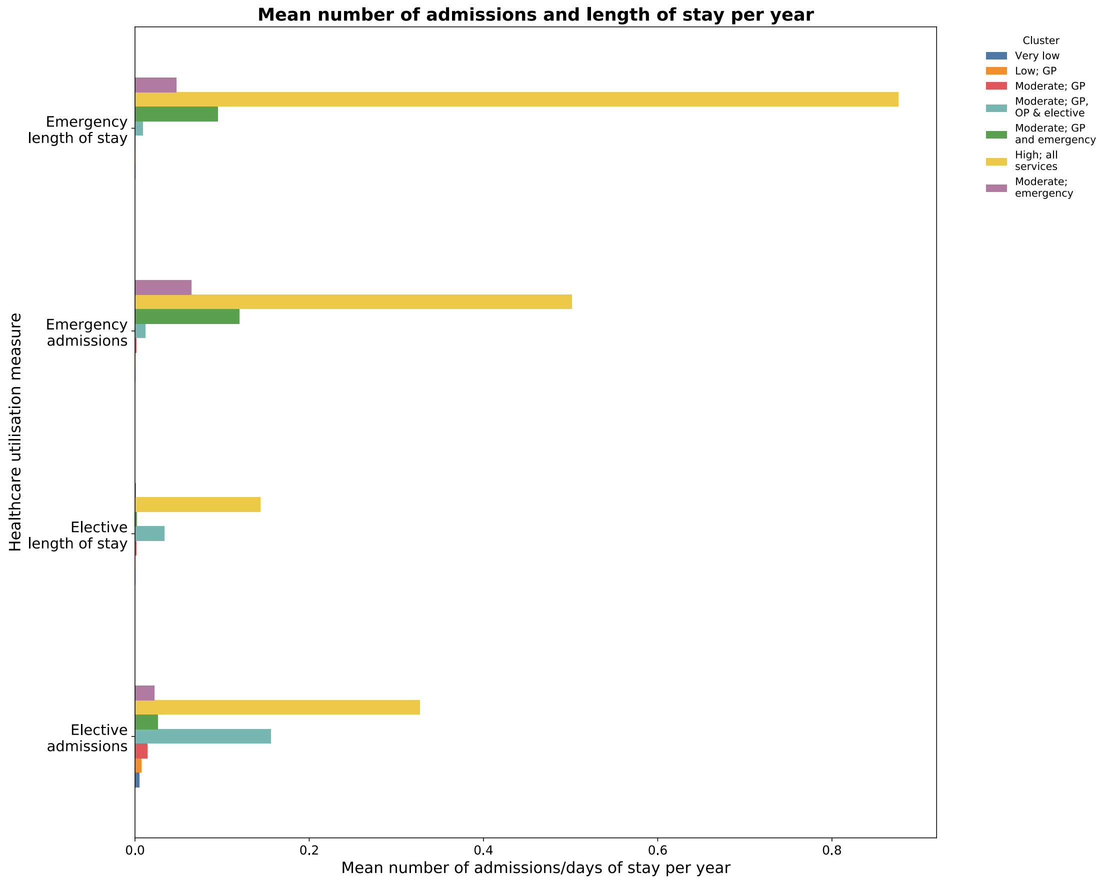

Figure S5a: Mean number of attendances to outpatients, GP and A&E from the 8 cluster model

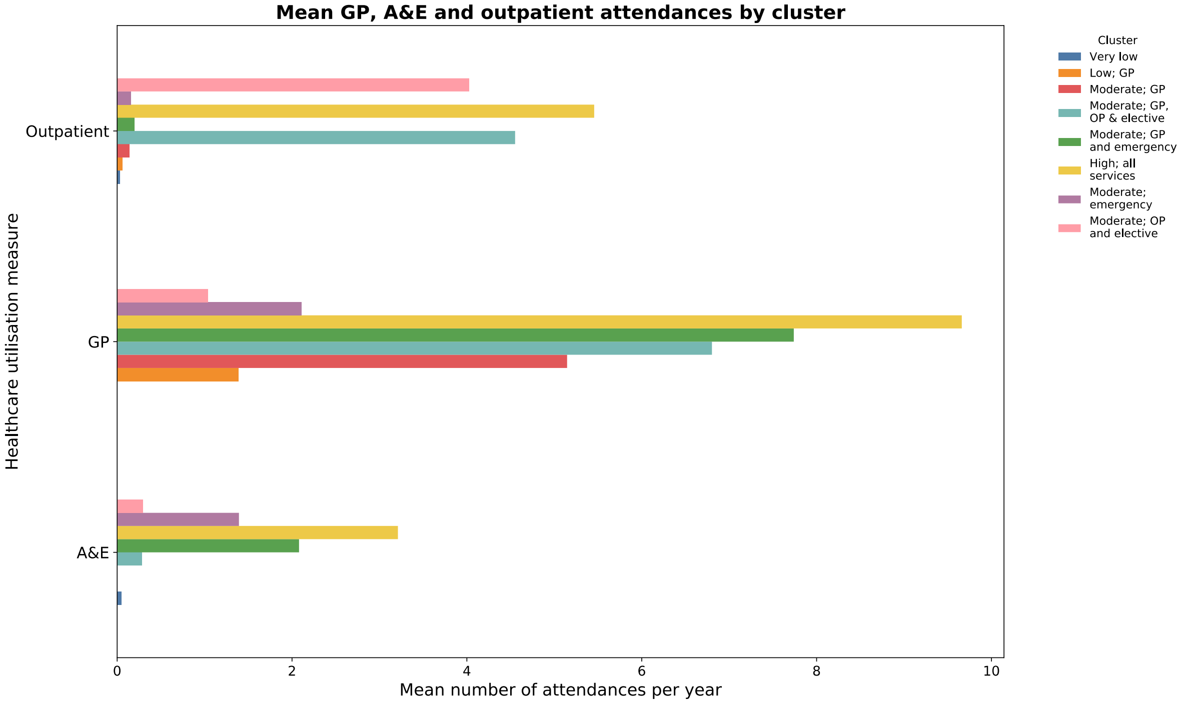

Figure S5b: Mean number of elective and emergency admission and yearly length of stay from the 8 cluster model

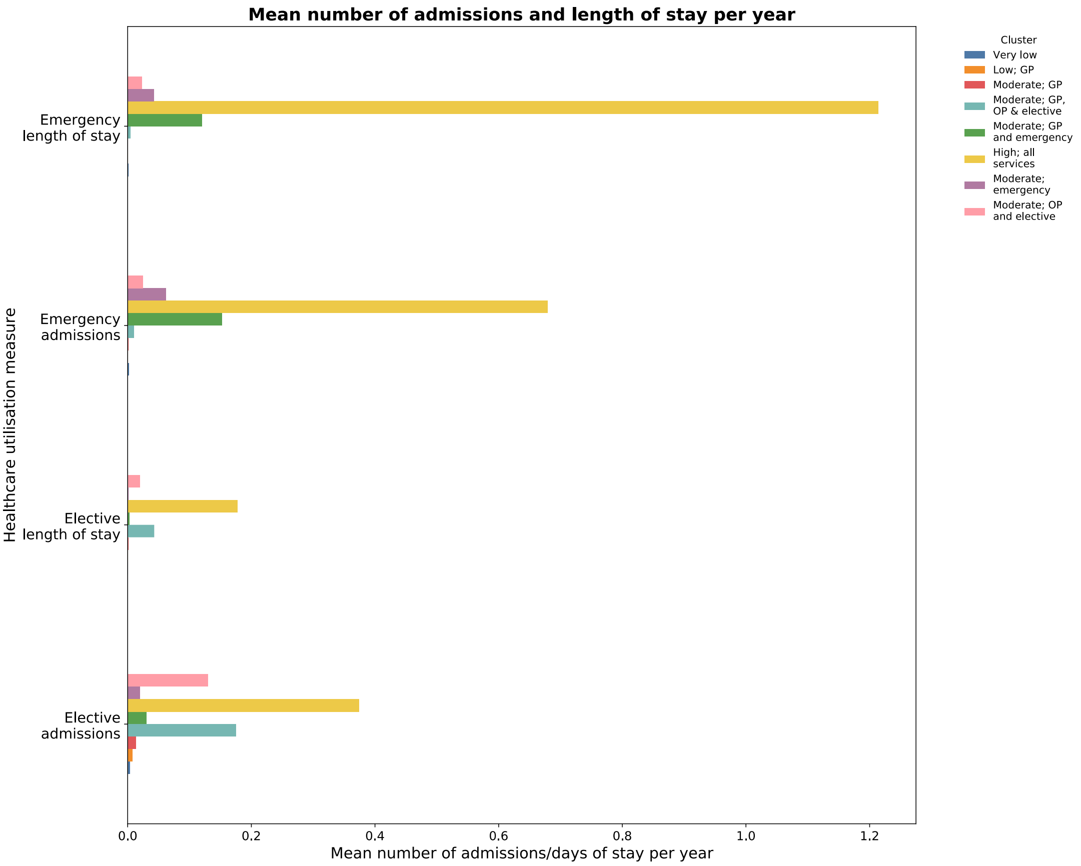

For peer review only - http://bmjopen.bmj.com/site/about/guidelines.xhtml

Figure S6: Confusion matrix for cluster assignment comparing 4 vs 5 clusters from k means

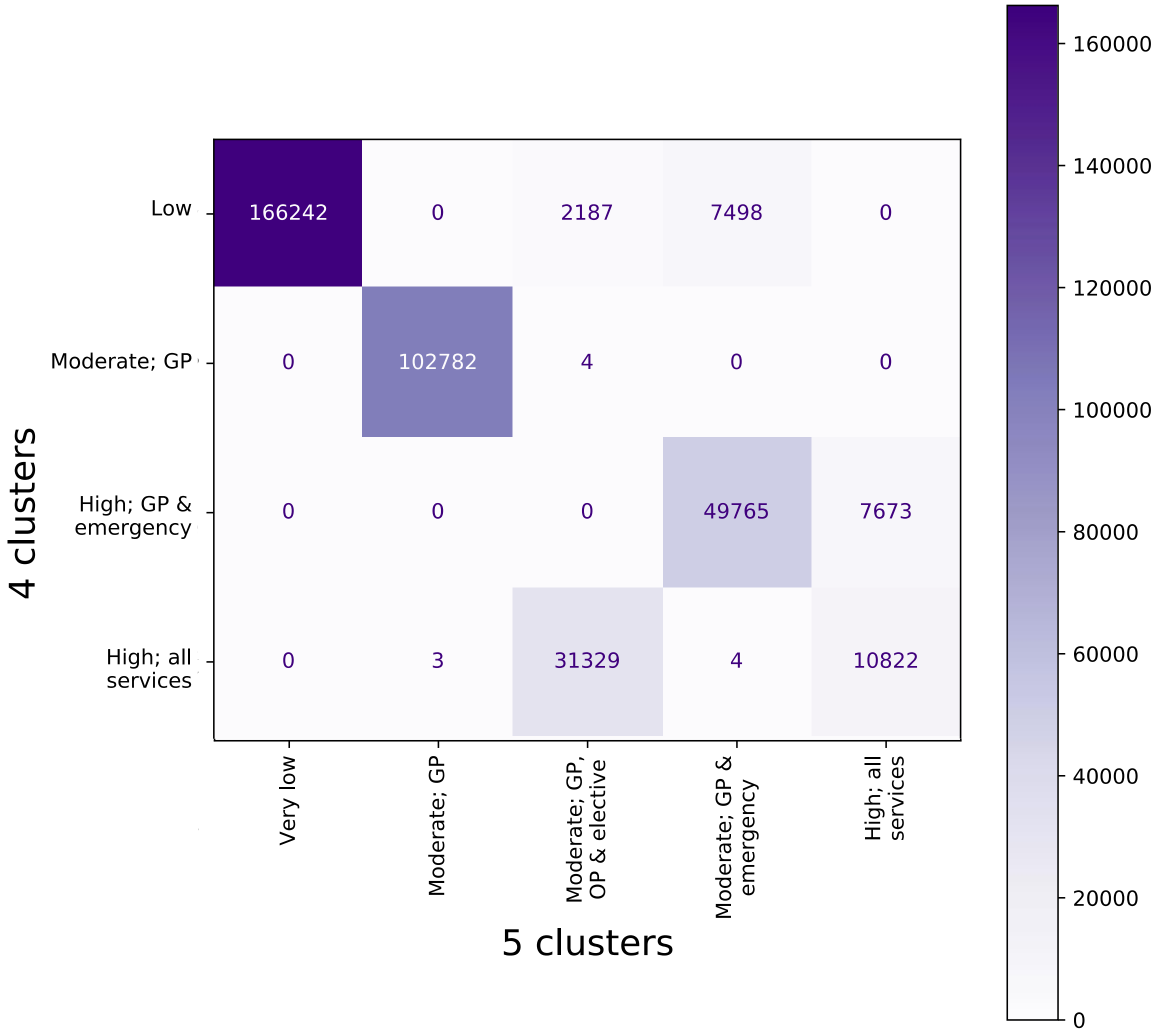

only

For peer review only - http://bmjopen.bmj.com/site/about/guidelines.xhtml

Figure S7: Confusion matrix for cluster assignment comparing 5 vs 6 clusters from k means

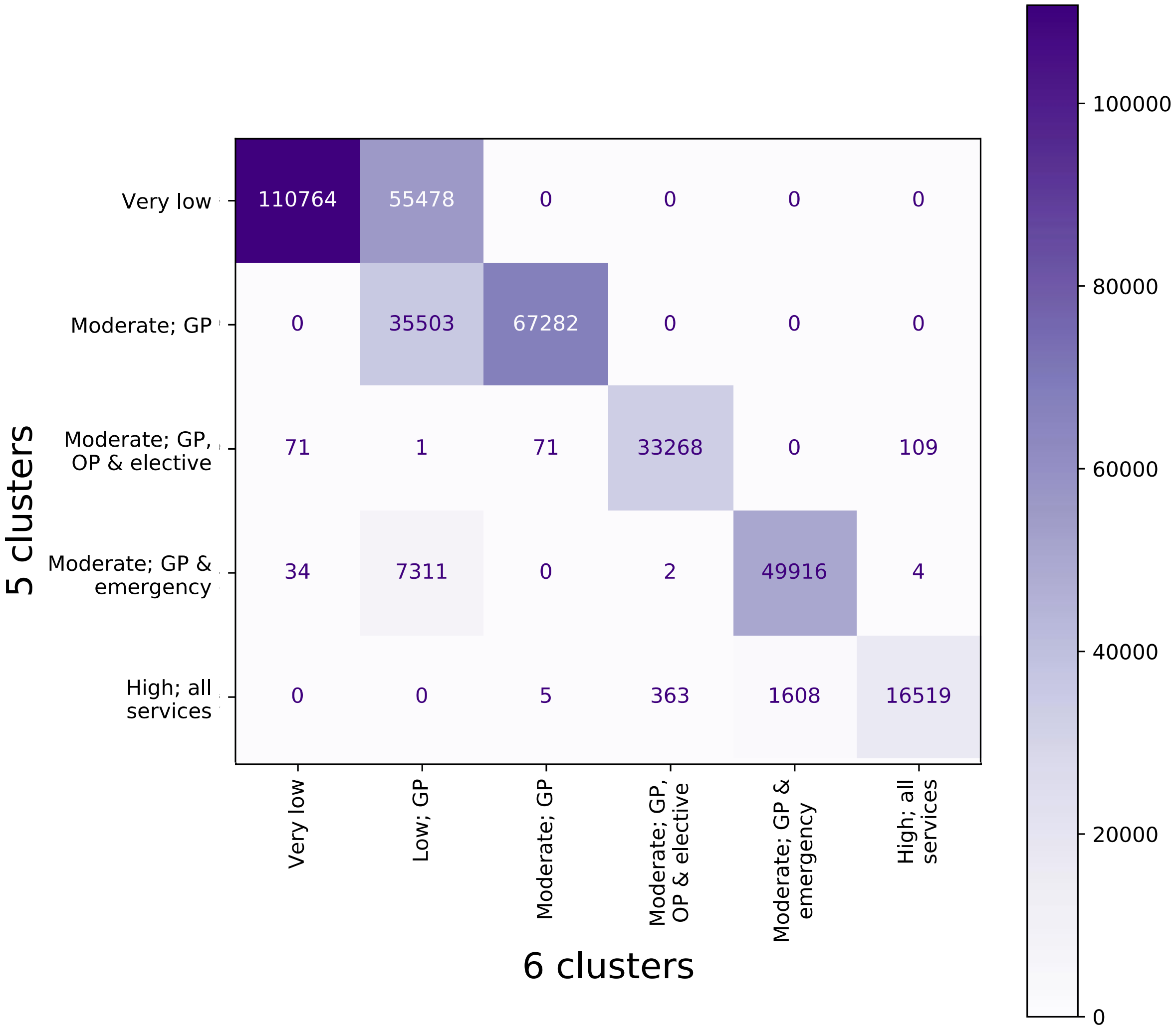

only

Figure S8: Confusion matrix for cluster assignment comparing 6 vs 7 clusters from k means

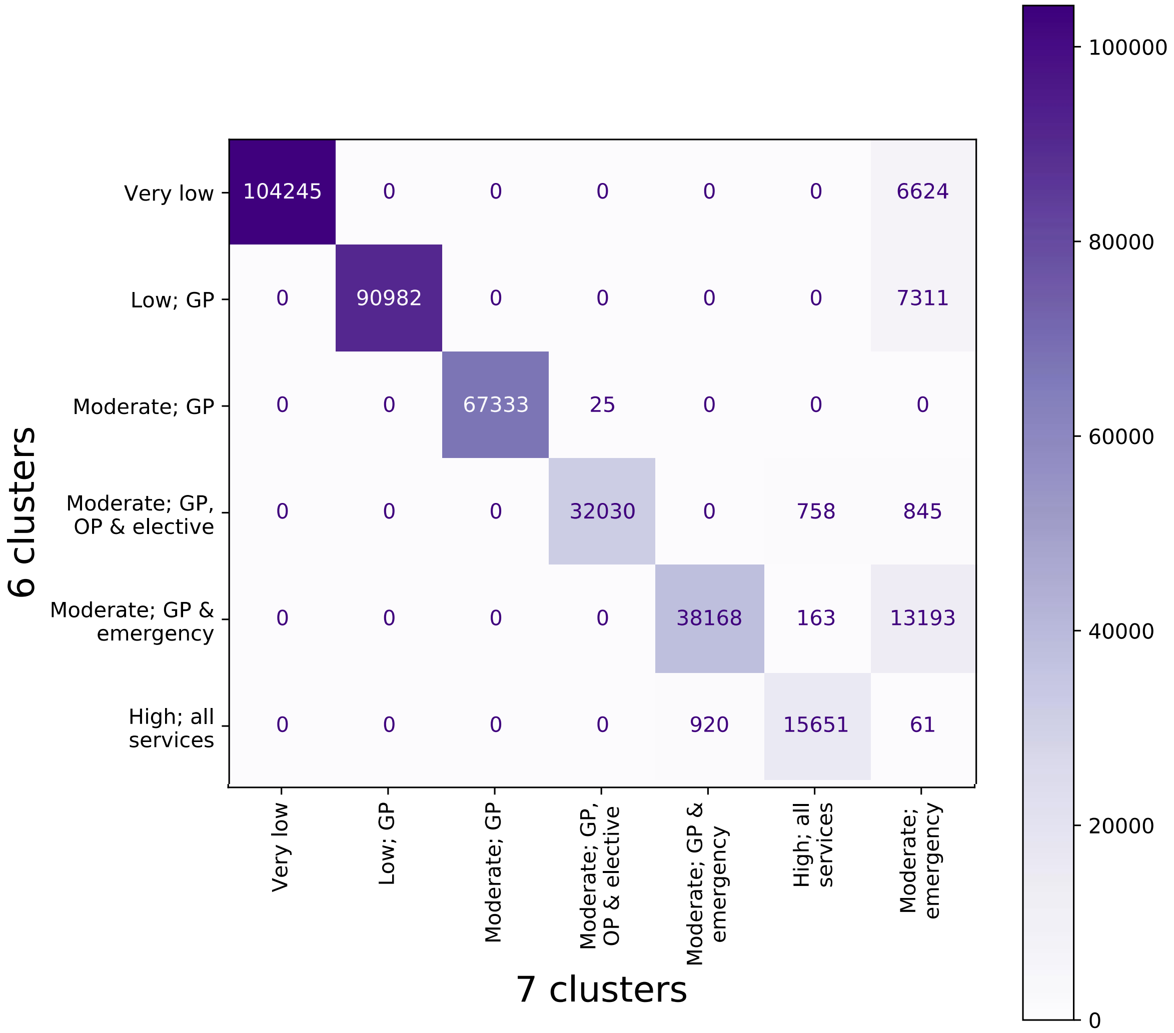

For peer review only - http://bmjopen.bmj.com/site/about/guidelines.xhtml

Figure S9: Confusion matrix for cluster assignment comparing 7 vs 8 clusters from k means

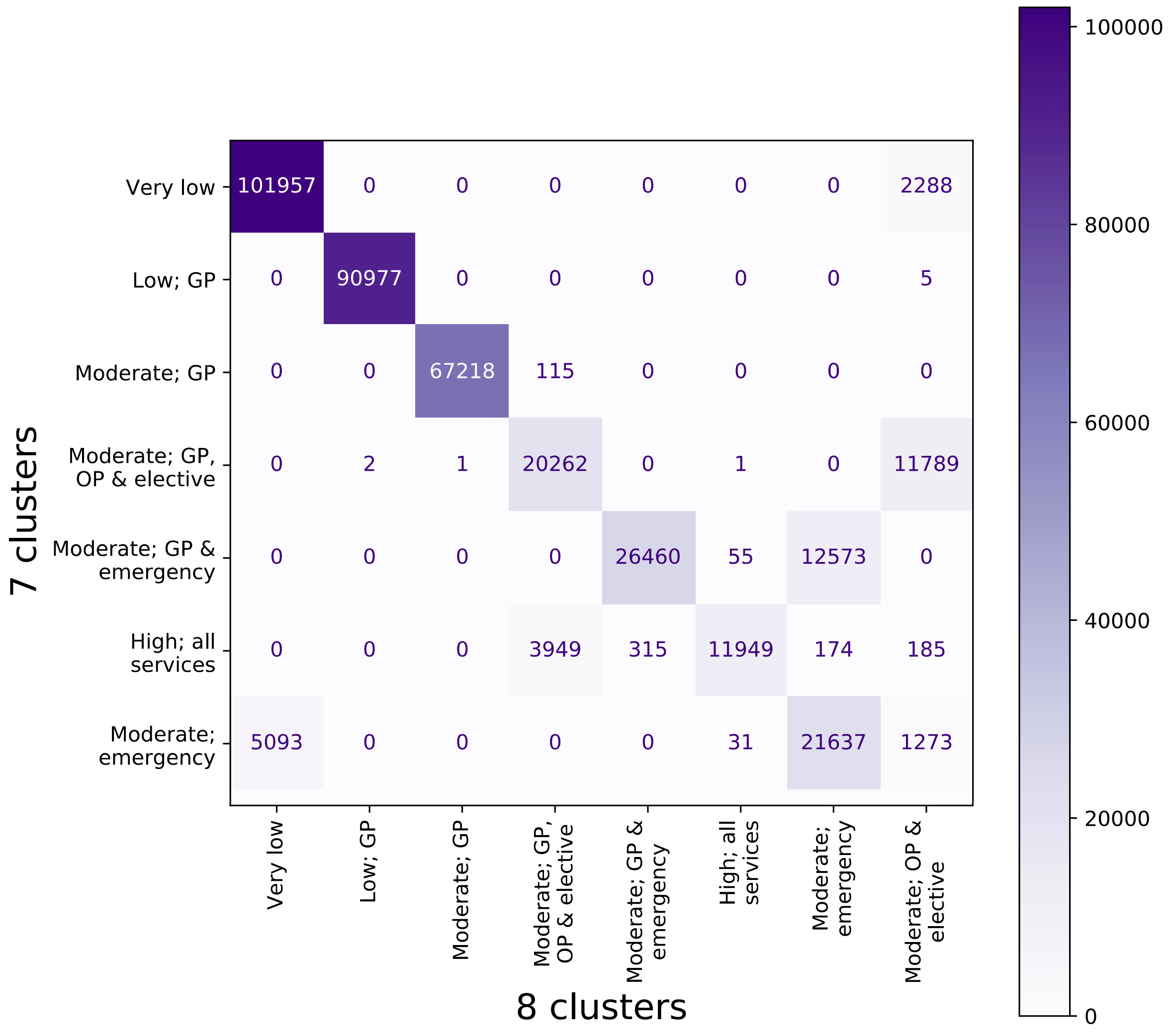

Figure S10: Confusion matrix for cluster assignment 6 clusters comparing models from k means and equivalent hierarchical clustering using Ward's method

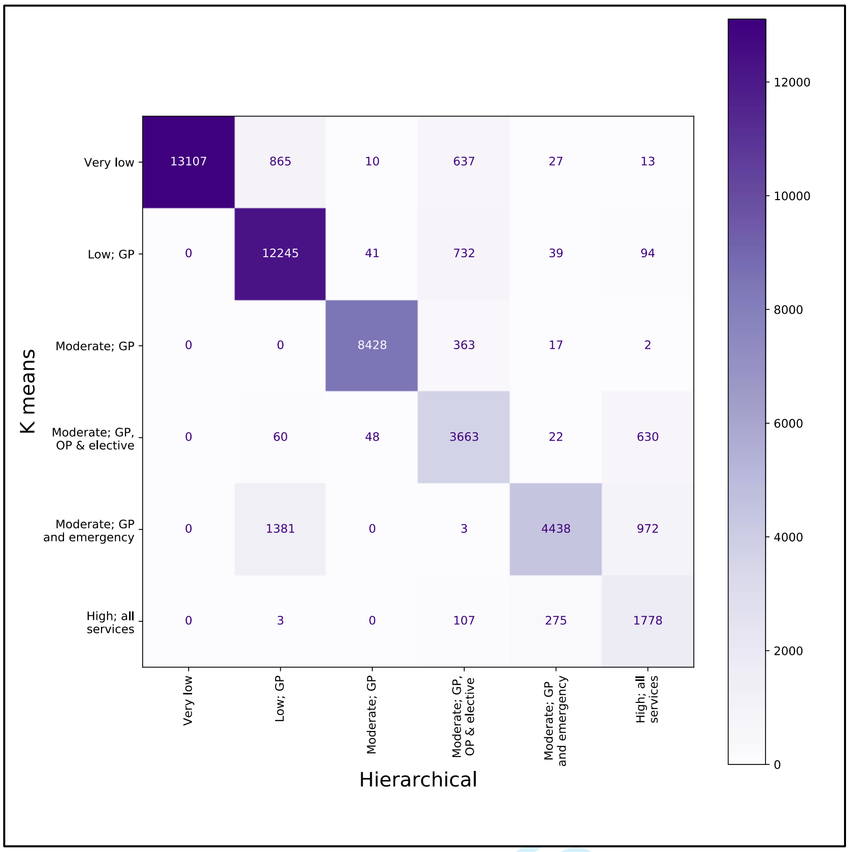

Table S4: Mean utilisation across each segment by utilisation variable from 6 cluster hierarchical model on subsample of 50,000 participants (green indicates lower than average utilisation, red indicates higher than average utilisation)

Segment	Size	Percentage	GP	Outpatient	A&E	Elective admissions	Elective length of stay	Emergency admissions	Emergency length of stay
I: No health service utilisation	12893	25.8%	0.00	0.00	0.00	0.00	0.00	0.00	0.00
II: Low; GP	13860	27.7%	1.70	0.00	0.00	0.00	0.00	0.00	0.00
III: Moderate; GP	5056	10.1%	6.27	0.01	0.00	0.02	0.00	0.00	0.00
IV: Moderate; GP, OP & elective	7497	15.0%	3.61	3.05	0.26	0.11	0.03	0.01	0.01
V: Moderate; GP & emergency care	7648	15.3%	4.20	0.03	1.71	0.02	0.00	0.11	0.08
VI: High; all services	3046	6.1%	8.61	4.15	2.22	0.24	0.07	0.38	0.67
**Total**	**50000**		**2.81**	**0.72**	**0.43**	**0.04**	**0.01**	**0.04**	**0.06**

Table S5: Median and interquartile range of presentations/length of stay over one year for each segment from 6 cluster k-means model (green indicates lower than average utilisation, red indicates higher than average utilisation)

k	Segment	Size	Percentage	GP	Outpatient	A&E	Elective admissions	Elective length of stay	Emergency admissions	Emergency length of stay
1	Very low	110869	29.3%	0 (0-0)	0 (0-0)	0 (0-0)	0 (0-0)	0 (0-0)	0 (0-0)	0 (0-0)
2	Low: GP	98293	26.0%	1 (1-2)	0 (0-0)	0 (0-0)	0 (0-0)	0 (0-0)	0 (0-0)	0 (0-0)
3	Moderate: GP	67358	17.8%	4 (3-6)	0 (0-0)	0 (0-0)	0 (0-0)	0 (0-0)	0 (0-0)	0 (0-0)
4	Moderate: GP, OP & elective	33633	8.9%	3 (2-6)	3 (2-5)	0 (0-0)	0 (0-0)	0 (0-0)	0 (0-0)	0 (0-0)
5	Moderate: GP & emergency	51524	13.6%	4 (2-6)	0 (0-0)	1 (1-2)	0 (0-0)	0 (0-0)	0 (0-0)	0 (0-0)
6	High: all services	16632	4.4%	8 (6-12)	3 (2-6)	2 (1-3)	0 (0-0)	0 (0-0)	0 (0-1)	0 (0-0)
	**Total**	**378309**		**2 (0-4)**	**0 (0-0)**	**0 (0-0)**	**0 (0-0)**	**0 (0-0)**	**0 (0-0)**	**0 (0-0)**

For peer review only - http://bmjopen.bmj.com/site/about/guidelines.xhtml

Table S6: Total healthcare associated costs for each segment from the 6-cluster k-means model

Segment	Size	Percentage	GP		Outpatient		A&E		Elective admissions		Emergency admissions		Total		Mean cost per capita (£)
			Total (£)	Percentage	Total (£)	Percentage	Total (£)	Percentage	Total (£)	Percentage	Total (£)	Percentage	Total (£)	Percentage	
I: Very low	110869	29.3%	0	0.0%	1677749	3.4%	846374	4.6%	700875	3.6%	315633	1.7%	3540631	2.5%	32
II: Low; GP	98293	26.0%	4415301	12.7%	1141363	2.3%	768704	4.2%	807133	4.1%	272191	1.4%	7404692	5.3%	75
III: Moderate; GP	67358	17.8%	11531157	33.3%	1862688	3.8%	0	0.0%	1095476	5.6%	71574	0.4%	14560895	10.4%	216
IV: Moderate; GP, OP & elective	33633	8.9%	4689465	13.5%	27200957	55.3%	823913	4.5%	7729103	39.5%	664487	3.5%	41107925	29.2%	1222
V: Moderate; GP & emergency care	51524	13.6%	8449386	24.4%	1691922	3.4%	10173249	55.4%	1406429	7.2%	5550515	29.6%	27271501	19.4%	529
VI: High; all services	16632	4.4%	5567562	16.1%	15639629	31.8%	5748710	31.3%	7832472	40.0%	11904047	63.4%	46692420	33.2%	2807
**Total**	**378309**		**34652871**		**49214308**		**18360950**		**19571488**		**18778447**		**140578064**		

For peer review only - http://bmjopen.bmj.com/site/about/guidelines.xhtml

For peer review only

Table S7: Population characteristics for each segment from 6-cluster k-means model

Participant characteristic		I: Very low		II: Low; GP		III: Moderate; GP		IV: Moderate; GP, OP & elective		V: Moderate; GP & emergency		VI: High; all services	
		Total	Proportion	Total	Proportion	Total	Proportion	Total	Proportion	Total	Proportion	Total	Proportion
**Gender**	Female	53,785	48.5%	49,476	50.3%	34,224	50.8%	15,485	46.0%	24,128	46.8%	7,310	44.0%
	Male	57,083	51.5%	48,816	49.7%	33,132	49.2%	18,147	54.0%	27,396	53.2%	9,321	56.0%
	Unknown	1	0.0%	1	0.0%	2	0.0%	1	0.0%	0	0.0%	1	0.0%
**Age (years) + female**	Mean (SD)	8.7 (4.0)		7.5 (4.3)		6.3 (4.8)		8.2 (4.6)		5.4 (4.7)		6.3 (5.1)	
	Median (IQR)	9 (6-12)		8 (4-11)		6 (2-10)		9 (4-12)		4 (1-9)		5 (1-11)	
	Under 1	1,064	1.0%	2,304	2.3%	4,106	6.1%	864	2.6%	4,211	8.2%	1,222	7.3%
	1-2	2,997	2.7%	5,422	5.5%	5,958	8.8%	1,386	4.1%	4,708	9.1%	1,198	7.2%
	3-4	4,553	4.1%	6,395	6.5%	5,144	7.6%	1,730	5.1%	3,550	6.9%	873	5.2%
	5-10	25454	23.0%	21398	21.8%	10461	15.5%	5643	16.8%	7176	13.9%	2076	12.5%
	11-15	19,717	17.8%	13,957	14.2%	8,555	12.7%	5,862	17.4%	4,483	8.7%	1,941	11.7%
**Age (years) + male**	Mean (SD)	8.9 (4.0)		7.7 (4.3)		6.1 (4.6)		7.8 (4.6)		5.4 (4.6)		5.5 (4.8)	
	Median (IQR)	9 (6-12)		8 (4-11)		5 (2-10)		8 (4-12)		4 (1-9)		4 (1-10)	
	Under 1	1,021	0.9%	2,036	2.1%	3,975	5.9%	1,084	3.2%	4,723	9.2%	1,694	10.2%
	1-2	3,038	2.7%	5,075	5.2%	5,698	8.5%	1,921	5.7%	5,398	10.5%	1,790	10.8%
	3-4	4,542	4.1%	5,976	6.1%	5,184	7.7%	2,203	6.6%	4,067	7.9%	1,342	8.1%
	5-10	25916	23.4%	21542	21.9%	11012	16.3%	6757	20.1%	8101	15.7%	2486	14.9%
	11-15	22,566	20.4%	14,187	14.4%	7,263	10.8%	6,182	18.4%	5,107	9.9%	2,009	12.1%
**Age (years)**	Mean (SD)	8.8 (4.0)		7.6 (4.3)		6.2 (4.7)		8.0 (4.6)		5.4 (4.6)		5.9 (4.9)	
	Median (IQR)	9 (6-12)		8 (4-11)		5 (2-10)		8 (4-12)		4 (1-9)		5 (1-10)	
	0	2,085	1.9%	4,340	4.4%	8,081	12.0%	1,948	5.8%	8,934	17.3%	2,916	17.5%
	1-2	6,035	5.4%	10,498	10.7%	11,656	17.3%	3,307	9.8%	10,106	19.6%	2,988	18.0%
	3-4	9,095	8.2%	12,371	12.6%	10,328	15.3%	3,933	11.7%	7,617	14.8%	2,215	13.3%

For peer review only

	5-10	51371	46.3%	42940	43.7%	21475	31.9%	12400	36.9%	15277	29.7%	4562	27.4%
	11-15	42,283	38.1%	28,144	28.6%	15,818	23.5%	12,045	35.8%	9,590	18.6%	3,951	23.8%
**Ethnicity**	Asian or Asian British	23,778	21.4%	24,124	24.5%	20,215	30.0%	7,749	23.0%	12,893	25.0%	3,945	23.7%
	White	27,950	25.2%	21,714	22.1%	12,481	18.5%	7,151	21.3%	10,726	20.8%	3,384	20.3%
	Mixed/multiple ethnic groups	10,425	9.4%	11,941	12.1%	7,060	10.5%	3,924	11.7%	6,376	12.4%	2,001	12.0%
	Other ethnic groups	10,566	9.5%	9,566	9.7%	6,889	10.2%	3,674	10.9%	5,581	10.8%	2,003	12.0%
	Black or Black British	7,749	7.0%	6,834	7.0%	4,225	6.3%	2,773	8.2%	3,138	6.1%	1,240	7.5%
	Unknown	30401	27.4%	24114	24.5%	16488	24.5%	8362	24.9%	12810	24.9%	4059	24.4%
**IMD quintile**	1 (most deprived)	15,282	13.8%	15,462	15.7%	10,670	15.8%	6,600	19.6%	9,101	17.7%	3,391	20.4%
	2	34,072	30.7%	31,174	31.7%	22,292	33.1%	10,792	32.1%	17,178	33.3%	5,666	34.1%
	3	29,050	26.2%	25,125	25.6%	17,163	25.5%	7,936	23.6%	13,028	25.3%	3,911	23.5%
	4	19,216	17.3%	15,457	15.7%	9,525	14.1%	4,453	13.2%	6,888	13.4%	1,906	11.5%
	5 (least deprived)	8,362	7.5%	7,054	7.2%	4,940	7.3%	2,385	7.1%	3,041	5.9%	931	5.6%
	Unknown	4,887	4.4%	4,021	4.1%	2,768	4.1%	1,467	4.4%	2,288	4.4%	827	5.0%
**Number of long-term conditions**	0	106,464	96.0%	91,248	92.8%	58,453	86.8%	27,083	80.5%	46,206	89.7%	12,457	74.9%
	One or more	4,015	3.6%	7,026	7.1%	8,890	13.2%	6,544	19.5%	5,308	10.3%	4,163	25.0%
	Missing	390	0.4%	19	0.0%	15	0.0%	6	0.0%	10	0.0%	12	0.1%
**Chronic condition***	Asthma	2,288	2.1%	4,469	4.5%	6,053	9.0%	3,128	9.3%	3,560	6.9%	2,161	13.0%
	Obesity	938	0.8%	1605	1.6%	1726	2.6%	993	3.0%	1139	2.2%	594	3.6%
	Mental health problem	276	0.2%	435	0.4%	631	0.9%	756	2.2%	398	0.8%	397	2.4%
	Learning Disability	84	0.1%	148	0.2%	394	0.6%	527	1.6%	133	0.3%	321	1.9%
	Chronic heart disease	245	0.2%	279	0.3%	237	0.4%	344	1.0%	186	0.4%	232	1.4%
	Chronic neurological disease & epilepsy	45	0.0%	93	0.1%	135	0.2%	508	1.5%	67	0.1%	461	2.8%
	Hypothyroidism	108	0.1%	125	0.1%	229	0.3%	396	1.2%	76	0.1%	154	0.9%

For peer review only - http://bmjopen.bmj.com/site/about/guidelines.xhtml

Cancer & immunosuppression	124	0.1%	152	0.2%	146	0.2%	315	0.9%	95	0.2%	229	1.4%
Diabetes	18	0.0%	16	0.0%	20	0.0%	440	1.3%	14	0.0%	267	1.6%
**Total participants**	**110,869**	**29.3%**	**98,293**	**26.0%**	**67,358**	**17.8%**	**33,633**	**8.9%**	**51,524**	**13.6%**	**16,632**	**4.4%**

* For those with ≥500 with condition. Proportion of those with non-missing data on LTC

For peer review only

References

1. Aitchison K, McGeown H, Holden B, Watson M, Klaber RE, Hargreaves D. Population child health: understanding and addressing complex health needs. Arch Dis Child. 2020. doi:10.1136/archdischild-2019-317373.

2. NHS England, British Medical Association. Seasonal influenza vaccination programme clinical used for payment. 2018. https://www.google.com/url?sa=t&rct=j&q=&esrc=s&source=web&cd=&cad=rja&uact=8&ved=2ahUKEwjBybDg5PXtAhUiVBUIHR34BxcQFjAJegQIAxAC&url=https%3A%2F%2Fwww.england.nhs.uk%2Fwp-content%2Fuploads%2F2019%2F07%2Fqsr-sfl-2019-20-codes-list.xlsm&usg=AOvVaw1Mt9mxgXhT_. Accessed 30 Dec 2020.

3. Davies DL, Bouldin DW. A Cluster Separation Measure. IEEE Trans Pattern Anal Mach Intell. 1979;PAMI-1:224–7.

4. Caliński T, Harabasz J. A dendrite method for cluster analysis. Commun Stat. 1974;3:1–27. doi:10.1080/03610927408827101.

5. Rosenberg A, Hirschberg J. V-Measure: A conditional entropy-based external cluster evaluation measure. EMNLP-CoNLL 2007 - Proc 2007 Jt Conf Empir Methods Nat Lang Process Comput Nat Lang Learn. 2007; June:410–20.

For peer review only - http://bmjopen.bmj.com/site/about/guidelines.xhtml

STROBE Statement—Checklist of items that should be included in reports of ***cohort studies***

	Item No	Recommendation	Page No
**Title and abstract**	1	(*a*) Indicate the study's design with a commonly used term in the title or the abstract<br>(*b*) Provide in the abstract an informative and balanced summary of what was done and what was found	2
**Introduction**			
Background/rationale	2	Explain the scientific background and rationale for the investigation being reported	4-5
Objectives	3	State specific objectives, including any prespecified hypotheses	5
**Methods**			
Study design	4	Present key elements of study design early in the paper	5-6
Setting	5	Describe the setting, locations, and relevant dates, including periods of recruitment, exposure, follow-up, and data collection	5
Participants	6	(*a*) Give the eligibility criteria, and the sources and methods of selection of participants. Describe methods of follow-up<br>(*b*) For matched studies, give matching criteria and number of exposed and unexposed	5-6
Variables	7	Clearly define all outcomes, exposures, predictors, potential confounders, and effect modifiers. Give diagnostic criteria, if applicable	6-8
Data sources/ measurement	8*	For each variable of interest, give sources of data and details of methods of assessment (measurement). Describe comparability of assessment methods if there is more than one group	6-8
Bias	9	Describe any efforts to address potential sources of bias	8
Study size	10	Explain how the study size was arrived at	5-6
Quantitative variables	11	Explain how quantitative variables were handled in the analyses. If applicable, describe which groupings were chosen and why	5-7
Statistical methods	12	(*a*) Describe all statistical methods, including those used to control for confounding<br>(*b*) Describe any methods used to examine subgroups and interactions<br>(*c*) Explain how missing data were addressed<br>(*d*) If applicable, explain how loss to follow-up was addressed<br>(*e*) Describe any sensitivity analyses	7-8
**Results**			
Participants	13*	(a) Report numbers of individuals at each stage of study—eg numbers potentially eligible, examined for eligibility, confirmed eligible, included in the study, completing follow-up, and analysed<br>(b) Give reasons for non-participation at each stage<br>(c) Consider use of a flow diagram	8
Descriptive data	14*	(a) Give characteristics of study participants (eg demographic, clinical, social) and information on exposures and potential confounders<br>(b) Indicate number of participants with missing data for each variable of interest<br>(c) Summarise follow-up time (eg, average and total amount)	9
Outcome data	15*	Report numbers of outcome events or summary measures over time	9

For peer review only

Main results	16	(*a*) Give unadjusted estimates and, if applicable, confounder-adjusted estimates and their precision (eg, 95% confidence interval). Make clear which confounders were adjusted for and why they were included<br>(*b*) Report category boundaries when continuous variables were categorized<br>(*c*) If relevant, consider translating estimates of relative risk into absolute risk for a meaningful time period	10-14
Other analyses	17	Report other analyses done—eg analyses of subgroups and interactions, and sensitivity analyses	14
**Discussion**			
Key results	18	Summarise key results with reference to study objectives	14-15
Limitations	19	Discuss limitations of the study, taking into account sources of potential bias or imprecision. Discuss both direction and magnitude of any potential bias	17-19
Interpretation	20	Give a cautious overall interpretation of results considering objectives, limitations, multiplicity of analyses, results from similar studies, and other relevant evidence	15-17
Generalisability	21	Discuss the generalisability (external validity) of the study results	17-18
**Other information**			
Funding	22	Give the source of funding and the role of the funders for the present study and, if applicable, for the original study on which the present article is based	19

*Give information separately for exposed and unexposed groups.

Note: An Explanation and Elaboration article discusses each checklist item and gives methodological background and published examples of transparent reporting. The STROBE checklist is best used in conjunction with this article (freely available on the Web sites of PLoS Medicine at http://www.plosmedicine.org/, Annals of Internal Medicine at http://www.annals.org/, and Epidemiology at http://www.epidem.com/). Information on the STROBE Initiative is available at http://www.strobe-statement.org.

For peer review only
